# Exploring Equilibria between Aluminium(I) and Aluminium(III): The Formation of Dihydroalanes, Masked Dialumenes and Aluminium(I) Species

**DOI:** 10.1002/anie.202205901

**Published:** 2022-05-17

**Authors:** Clare Bakewell, Katie Hobson, Claire J. Carmalt

**Affiliations:** ^1^ Department of Chemistry King's College London 7 Trinity Street London SE1 1DB UK; ^2^ Department of Chemistry University College London 20 Gordon Street London WC1H 0AJ UK

**Keywords:** Aluminium, Low Oxidation State, Masked Dialumene, Redox, Reversible

## Abstract

The design of new reductive routes to low oxidation state aluminium (Al) compounds offers the opportunity to better understand redox processes at the metal centre and develop reactivity accordingly. Here, a monomeric Al^I^ compound acts as a stoichiometric reducing agent towards a series of Al^III^ dihydrides, leading to the formation of new low oxidation state species including symmetric and asymmetric dihydrodialanes, and a masked dialumene. These compounds are formed by a series of equilibrium processes involving Al^I^, Al^II^ and Al^III^ species and product formation can be manipulated by fine‐tuning the reaction conditions. The transient formation of monomeric Al^I^ compounds is proposed: this is shown to be energetically viable by computational (DFT) investigations and reactivity studies show support for the formation of Al^I^ species. Importantly, despite the potential for the equilibrium mixtures to lead to ill‐defined reactivity, controlled reactivity of these low oxidation state species is observed.

## Graphical abstract

A monomeric Al^I^ compound is used as a stoichiometric reducing agent towards a series of Al^III^ dihydrides. This leads to the formation of new low oxidation state species including symmetric and asymmetric dihydrodialanes, and a masked dialumene. The transient formation of new, previously inaccessible Al^I^ species is demonstrated through reactivity studies and theory.

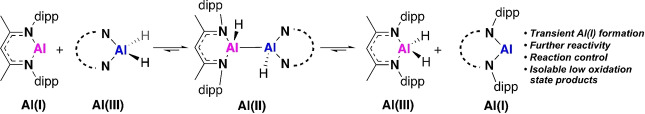

Low oxidation state aluminium compounds are becoming established in synthetic methodology for controlled chemical reductions, having been shown to cleave a wide range of strong chemical bonds.[^1^, ^2^] Until recently, only a handful of low oxidation state aluminium compounds were known, but work from several research groups has seen a rise in the number of stable and isolable monomeric and dimeric Al^I^ and Al^II^ compounds.[^3^, ^4^] However, the scope of these reactions remains limited to a fairly select group of supporting ligands that are able to tolerate the harsh reductive conditions required to form the low oxidation state Al centre.

The use of milder, more targeted reducing agents is one strategy that could allow access to a more varied pool of low oxidation state aluminium compounds. Jones’ Mg^I^ dimer has already been employed as a stoichiometric reducing agent to great effect for a range of metal compounds.[^5^, ^6^, ^7^] In 2014, Nikonov and co‐workers reported the reduction of an Al^III^ dihydride, **B**, with the Al^I^ monomer, **A**, to form the dihydrodialane, **C** (Figure 1a).^[8]^ Species **A**–**C** were all found to exist in equilibrium, with **C** forming in approximately 50 % yield at room temperature. Cowley and co‐workers subsequently proposed the reverse reaction to occur from the dihydrodialane, **D**, which was itself formed by the reduction of the Al^III^ dihydride with a Mg^I^ source at high temperature (Figure 1b). Compound **D** was shown to interconvert between a number of different diastereomers and undergo crossover reactions with different Al^III^ dihydrides, which strongly supports the formation of **F**, though it was not directly observed.^[9]^ Subsequent work reported isolation of the dialumene analogue of **F**, which upon treatment with an alkyne formed an aluminium cyclopropene, supporting disproportionation to monomeric **F** followed by a 2+1 cycloaddition.^[10]^ These reactions are rare examples of reversible redox behaviour at a main‐group centre, with Al switching between the Al^I^/Al^III^ and Al^II^/Al^II^ oxidation states. Other notable examples include C−H and C−C bond breaking reactions, reversible alkene coordination and [4+1] cycloadditions.[^11^, ^12^, ^13^, ^14^, ^15^, ^16^, ^17^]

**Figure 1:**
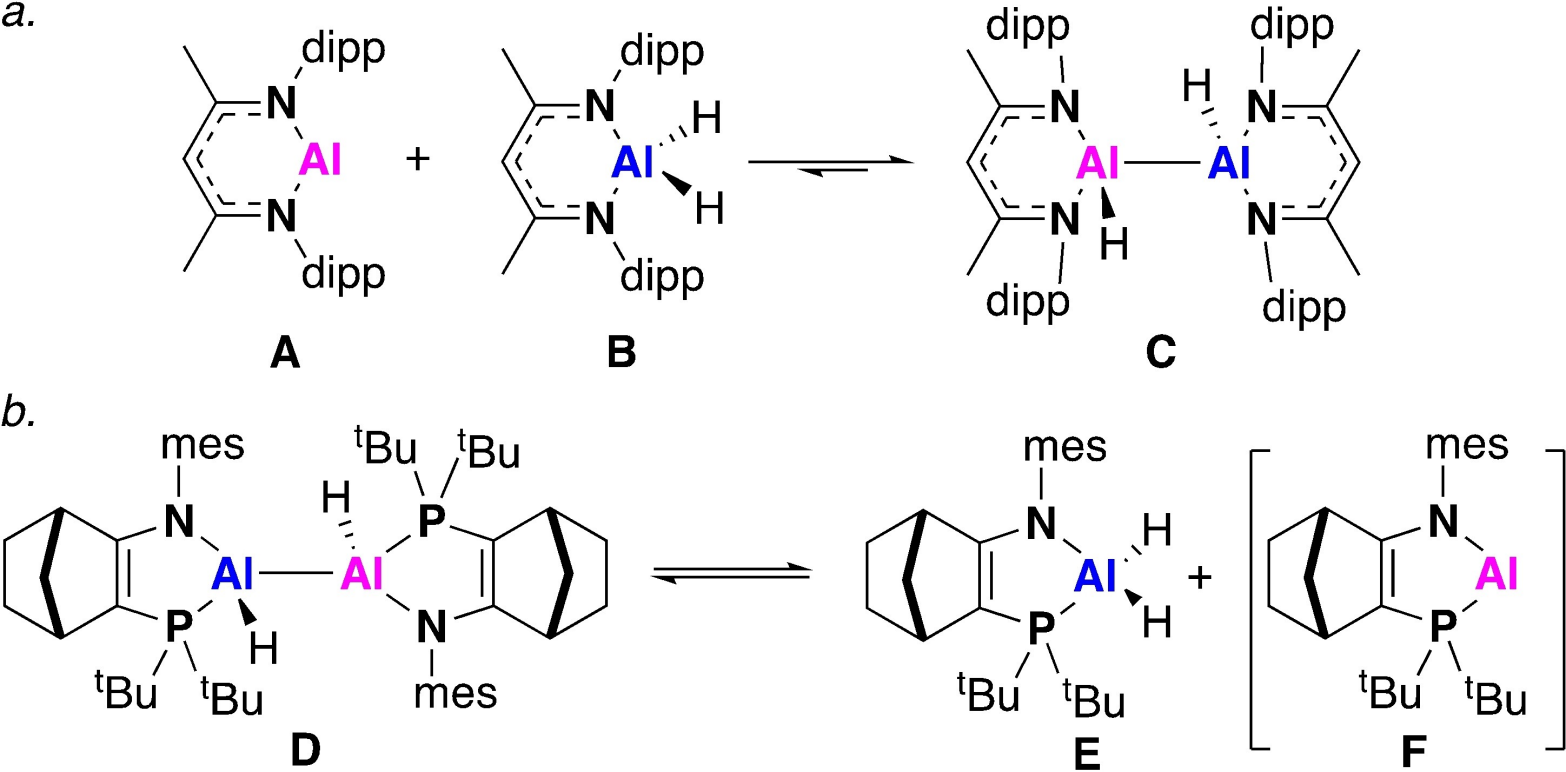
Examples of reversible reactions involving Al^I^ and Al^III^ centres (dipp=2,6‐diisopropylphenyl, mes=2,4,6‐trimethylphenyl, tBu=*tert*‐butyl).

In terms of reactivity the equilibrium between **A**, **B** and **C** (Figure 1a) is limiting due to the mix of species present, however, the masked reactivity of **D** provides some indication of how such equilibria may be harnessed. Manipulation of the equilibrium between Al^I^, Al^II^ and Al^III^ provides the opportunity to create synthetic routes to well‐defined low oxidation state species that cannot be accessed by traditional reductive methods. Targeting asymmetric systems is one such methodology which could allow these equilibria to be exploited. Herein, we report the use of the monomeric Al^I^ compound, **A**, as a controlled and selective reducing agent for a series of Al^III^ dihydrides. This reveals a series of equilibrium processes leading to the formation of new low oxidation state compounds, including symmetric and asymmetric dihydrodialanes and a masked dialumene. These compounds are proposed to form via a monomeric Al^I^ species; this has been probed through DFT and experiment both of which support transient Al^I^ formation.

The 1 : 1 reaction of **A** and the Al^III^ dihydride (**1**) in benzene‐*d*_6_ at 25 °C saw the immediate formation of a new asymmetric product, proposed to be the dihydrodialane, **2** (Scheme 1a).^[18]^ This contained two characteristic Al−*H* resonances (4.54 and 5.05 ppm) in the ^1^H NMR spectrum integrating to one proton each, with the two ligands present in a 1 : 1 ratio (Figure S1). The product formed with concomitant formation of two known literature compounds, namely the Al^III^ dihydride, **B**, and the dihydrodialane, **3**.[^5^, ^19^] Manipulation of the reaction stoichiometries allowed product formation to be controlled. Conducting the reaction with a 1 : 2 stoichiometry of **A** to **1** first led to the aforementioned mixture of compounds, but after 24 h at room temperature this mixture had equilibrated, with **3** and **B** being the sole products of the reaction (Scheme 1b and Figure S2). Similarly, conducting the reaction with a slight excess of **A** at 25 °C, led to the exclusive formation of the dihydrodialane **2**, with no evidence of products **3** and **B** forming when the reaction was monitored over several days. Whilst it was not possible to obtain crystals suitable for single crystal XRD, DOSY NMR analysis in benzene‐*d*_6_ confirmed all the proton signals assigned to **2** belong to the same species (Figure S3, S33).^[20]^

**Scheme 1:**
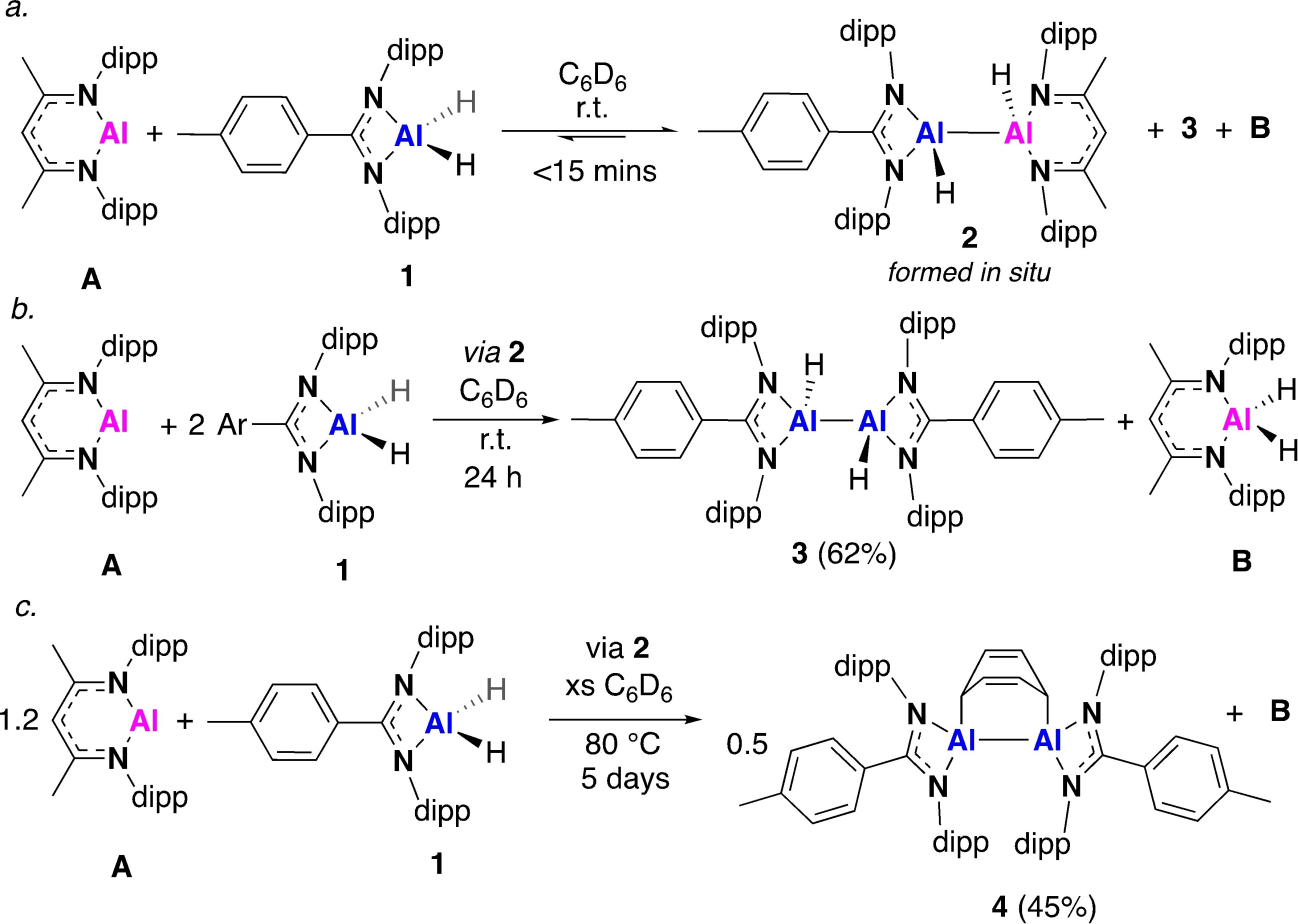
The reaction of **A** and **1** (dipp=2,6‐diisopropylphenyl, Ar=*p*‐tolyl). Isolated yield (%).

The formation of the Al^III^ dihydride, **B**, provides strong evidence for a monomeric Al^I^ intermediate, which in the presence of a second equivalent of **1**, goes on to form the symmetric dihydrodialane **3** (Scheme 2). Further evidence for the formation of an Al^I^ species was obtained by heating a sample of **2**, formed by reaction of **1** with a slight excess of **A** in benene‐*d*_6_ at 80 °C (Scheme 1c). After 1 hour a bright red solution was observed, and continued heating for 5 days led to the clean formation of a new amidinate compound (Figure S4, ^1^H NMR spectrum), in addition to **B**. Bright red crystals suitable for single crystal X‐ray diffraction were isolated from a hexane solution and revealed the formation of the dialumene:benzene adduct, **4** (Figure 2, Table 1). The complex is highly distorted, with the amidinate ligands pinched back, exposing the dialumene:benzene ring system. The Al−Al bond length (2.5419(7) Å) is shorter than that of two previously reported examples of dialumene:arene adducts,[^21^, ^22^] but longer than that of the recently reported dialumene from Cowley and co‐workers (2.5190(14) Å).^[10]^ The bond lengths in the benzene fragment are indicative of a dearomatized cycloaddition product with two double bonds (C2−C3, 1.337(3) Å; C5−C6, 1.338(4) Å) and four single bonds (1.497–1.501 Å). The Al−C bond lengths (Al1−C1, 2.017(2) Å; Al2‐C4, 2.018(2) Å) are comparable to a previously reported dialumene:benzene adduct,^[22]^ but slightly longer than in a related dialumene:toluene adduct.^[21]^ Compound **4** is proposed to form via a cycloaddition reaction with a dialumene intermediate, which itself forms from a monomeric Al^I^ species generated by the disproportionation of **2** (**AmAl(I)‐d**, Scheme 2).^[23]^ A related adduct was reported by Power and co‐workers, following treatment of an aluminium diiodide with potassium graphite in toluene.^[21]^ This was also proposed to occur via the transient formation of a dialumene, but only the partially reduced 1,2‐diiodoalane could be isolated. Analysis of **4** by ^13^C{^1^H} NMR did not show any signals for the C_6_D_6_ fragment, and only two broad resonances at 2.68 and 5.35 ppm were observed in the ^2^H NMR spectrum. This indicates that the C_6_D_6_ fragment is undergoing dynamic exchange processes, a phenomenon that was also observed by Tokitoh and co‐workers in a dialumene:benzene adduct.^[22]^ However, attempts to exchange the benzene for alternative arenes or alkynes were unsuccessful suggesting exchange is intramolecular.^[24]^ Similarly, there was no reaction with dihydrogen which has been observed for other dialumene:benzene adducts, perhaps due to the comparative steric bulk of the amidinate ligand.^[25]^

**Scheme 2:**
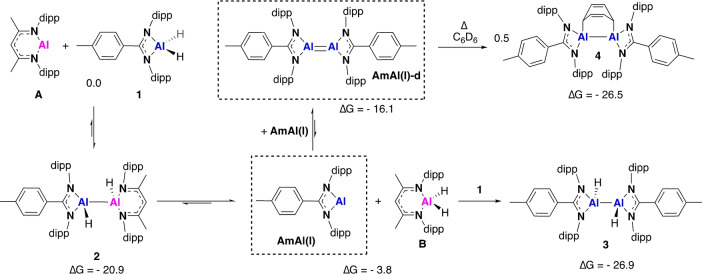
Proposed reaction pathway for the formation of **2**–**4** and **B**. Species in dashed boxes are not directly observed, but inferred based of product formation (M06L; Al (SDDAll), C H N (6‐31G**)+Δ*E*_solv_ (PCM, benzene)). Gibbs free energies relative to starting reagents, normalised versus **A+1** (kcal mol^−1^).

**Figure 2:**
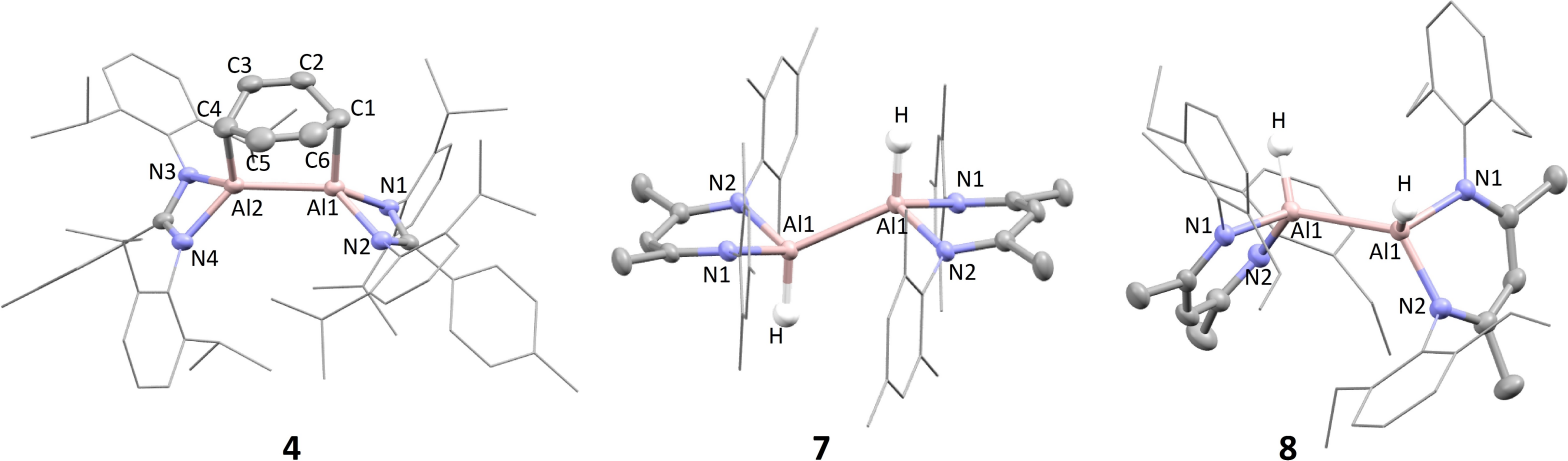
Solid‐state structures of compounds **4**, **7** and **8**.

**Table 1:** Table of bond lengths [Å] and bond angles [°] for compounds **4**, **7** and **8**.

	Al1−Al2	Al1−C1	Al2−C4	C1−C2	C3−C4
**4**	2.5419(7)	2.017(2)	2.018(2)	1.501(3)	1.337(3)
	Al−Al	Al1−H	Al1−N1	Al1−N2	N1−Al1−N2
**7**	2.6353(8)	1.51(3)	1.940(1)	1.947(1)	93.4
**8**	2.6315(5)	1.51(2)	1.942(1)	1.935(1)	92.1

The reaction of **1** and excess **A** (Scheme 1c), as well as a 1 : 1 reaction of **3** and **A**, were conducted in cyclohexane‐d_12_, in an attempt to isolate the arene‐free dialumene. In both cases, peaks corresponding to a new amidinate compound were observed in the ^1^H NMR spectra (Figures S8–S10 and S12). However, neither reaction could be driven to completion indicating the species exist in equilibrium and all attempts to isolate crystals have thus far been unsuccessful.^[26]^

In order to further investigate the formation of monomeric Al^I^ and dialumene intermediates, asymmetric reactivity was expanded to include a series of β‐diketiminate Al^III^ dihydride compounds. Reaction of two equivalents of **5** with **A** in benzene‐*d*_6_ at 25 °C led to the facile formation (<3 h) of the aluminium dihydride **B** and the symmetric dihydrodialane, **7** (Scheme 3a). During the course of the reaction two characteristic peaks corresponding to the backbone methine proton of the β‐diketiminate ligands were observed to form in a 1 : 1 ratio at 4.57 and 4.73 ppm in the ^1^H NMR spectrum (Figure S5). This is indicative of an asymmetric dihydrodialane akin to **2**, however, its formation was short lived and manipulation of the reaction stoichiometries consistently led to a mixture of products. Compound **7** displayed a single characteristic Al−*H* resonance at 4.70 ppm in the ^1^H NMR spectrum, and single crystal XRD analysis confirmed the formation of a dihydrodialane with terminal hydrides, which were freely located (Figure 2, Table 1). This had a typical structure involving a distorted tetrahedral geometry at aluminium, where the two aluminium hydrides face in opposing directions.^[5]^ The Al−Al bond length (2.6353(8) Å) was similar to that observed for the amidinate analogue, **5**, and within the range of bond lengths observed for the series of dihydrodialanes (**D**) reported by Cowley and co‐workers (2.6586(16)–2.886(2) Å).[^5^, ^9^] Near identical reactivity was observed for the 2,6‐diethylphenyl (dep) substituted Al^III^ dihydride (**6**), leading to formation of the symmetric dihydrodialane, **8**, in less than 1 hour (Scheme 3a). Orange crystals of **8** were grown from hexane and the structure was shown to be significantly more distorted (Figure 2, Table 1), with the terminal aluminium hydride planes just 73° apart (**7**: 180°). That said, bond lengths and angles were similar to that of **7** and no significant difference in the Al−Al bond was observed. As in the amidinate system (Scheme 2), both **7** and **8** are proposed to form via a monomeric Al^I^ intermediate. In contrast, the stoichiometric reaction of **A** with the less sterically hindered Al^III^ dihydride **9** led to the immediate formation of the asymmetric dihydrodialane, **10** (Scheme 3b). This species was persistent in solution, and only after 7 days in benzene‐*d*_6_ was there any evidence for the formation of **B**. No further reaction was observed after addition of a second equivalent of **9** and heating the reaction for 1 h at 80 °C only resulted in formation of **B**, along with various unidentified degradation products (Figure S6). This indicates that in this instance the proposed monomeric Al^I^ intermediate is highly unstable, which exposes the limits of β‐diketiminate stabilised low oxidation state aluminium species. Compound **10** was found to co‐crystallise with a small amount of a hydroxide decomposition product (10 %, Figure S22). Nevertheless, it can be determined that the hydrides lie in alternate planes of the molecule and that the Al−Al bond length is comparable to **7** and **8**. The N−Al−N bond lengths of **7**, **8** and **10** (91.6–93.8°), which are formally Al^II^, sit in between the those observed for **A** (89.9°) and related Al^III^ compounds (≈96°).^[27]^

**Scheme 3:**
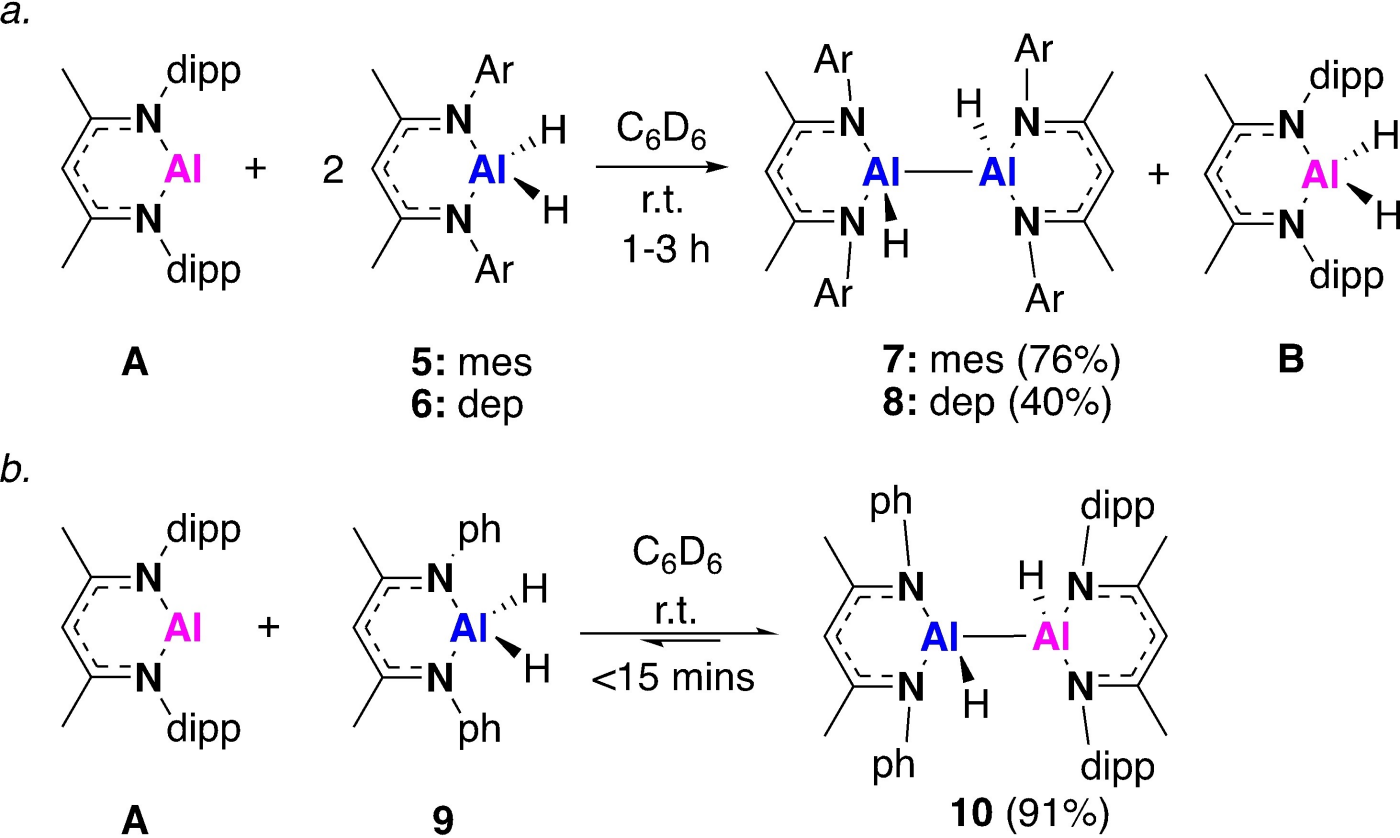
The reaction of **A** with β‐diketiminate Al^III^ dihydrides **5**, **6** and **9** (dipp=2,6‐diisopropylphenyl, mes=2,4,6‐trimethylphenyl, dep=2,6‐diethylphenyl). Isolated yield (%).

To further explore the feasibility of a monomeric Al^I^ intermediate in the formation of **3**, **4**, **7** and **8**, we sought to investigate the equilibrium processes using DFT. Geometries were optimised in the gas phase, using the M06 L functional and a split 6‐31G**(C, H, N)/SDDAll (Al) basis set; a single point benzene solvent correction (PCM solvent model) was applied. The formation of the asymmetric dimer, **2**, was found to be exergonic relative to **A** and **1** (Δ*G*=−20.9 kcal mol^−1^). However, from here disproportionation of **2** to the alternate Al^I^/Al^III^ species (**AmAl(I)** and **B**) was endergonic, Δ*G*=17.1 kcal mol^−1^, although overall this combination is slightly more favourable than **A** and **1** (Δ*G*=−3.8 kcal mol^−1^). Cao and co‐workers have previously proposed that the related dihydrodialane, **C**, forms via a low energy transition state (≈8 kcal mol^−1^), as estimated by a scan of the potential energy surface. However, in both their work and here it was not possible to locate a precise transition state.^[28]^ Dimerisation of **AmAl(I)**, to form **AmAl(I)‐d**, was found to be slightly more energetically favourable (−12.3 kcal mol^−1^) suggesting a transient low oxidation state species is more likely to exist as a dialumene. Initial DFT calculations indicate **AmAl(I)‐d** has a *trans*‐bent structure similar to that observed for the dialumene reported by Cowley and co‐workers, which was found to have small but significant multiple bond character.^[10]^ The formation of **3** and **4** are both equally favourable, with just 0.4 kcal mol^−1^ separating the energies of the products. However, experimentally, we only observe **4** forming at elevated temperature, indicating this pathway has a higher energy transition state(s). Compounds **3** and **4** are both reaction sinks and their formation appears to be non‐reversible. Experimentally, heating a 1 : 1 mixture of **3** and **B** does not result in the formation of **4**, however, **3** can react with one equivalent of **A** at 80 °C to yield **4** and **B** (Figure S7, S11).^[29]^

Formation of the asymmetric dihydrodialane in the reaction of **A** with **5** was less favourable than for **2** ((Δ*G*=−12.0 kcal mol^−1^), which may explain the short‐lived nature of this intermediate (Figure S27). Experimentally, the facile formation of the symmetric dihydrodialane, **7**, was observed, a process which was much slower with **1** and **A**. This can be rationalised by the monomer dimer equilibrium, ^mes^BDIAl(I) to ^mes^BDIAl(I)‐dimer, being slightly endergonic (Δ*G*=1.8 kcal mol^−1^) meaning reaction with a second equivalent of **5** to form the dihydrodialane is more favourable than in the amidinate system (Figure S27).

DFT indicates monomeric Al^I^ species are viable intermediates in the formation of the dihydrodialanes **3**, **7** and **8**, as well as the masked dialumene, **4**. The formation of **3**, however, appears to be non‐reversible;^[30]^ in contrast, the formation of compound **7** is less thermodynamically favourable (Δ*G*=−5.2 kcal mol^−1^, Figure S27), indicating reversibility reactivity might be observed. Conducting a variable temperature ^1^H NMR experiment on the products of a 1 : 1 reaction of **A** and **5** showed consumption of the proposed asymmetric dihydrodialane intermediate and reformation of **A** at high temperature (Figure S13). However, there was no change in the spectrum of a 1 : 1 mix of **7** and **B** (Figure S14). We therefore sought to trap the proposed low oxidation state intermediates using reagents known to oxidatively add to Al^I^ centres.[^31^, ^32^] However, reaction of **7** with one equivalent of diphenylacetylene instead saw alkyne addition directly to the Al−Al bond, suggesting this process is faster than disproportionation of the Al^II^ dimer. No reaction was observed at room temperature, but after 1 h at 80 °C clean formation of the 1,2‐dialumination product (**11**) was observed (Scheme 4a). Single crystal XRD showed compound **11** to exists with *Z*‐stereochemistry at the alkene with a C−C bond length of 1.368(3) Å (Figure 3). Related insertions into Al−Al single bonds have previously been reported, but this is the first example from a dihydrodialane.[^33^, ^34^]

**Scheme 4:**
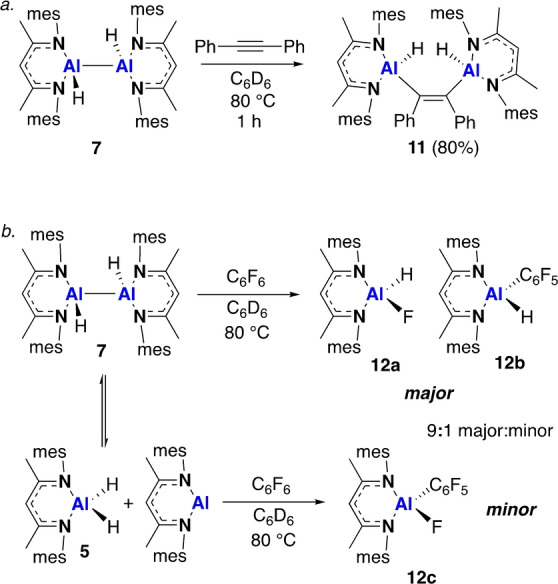
The reaction of **7** with a) diphenylacetylene and b) hexafluorobenzene.

**Figure 3:**
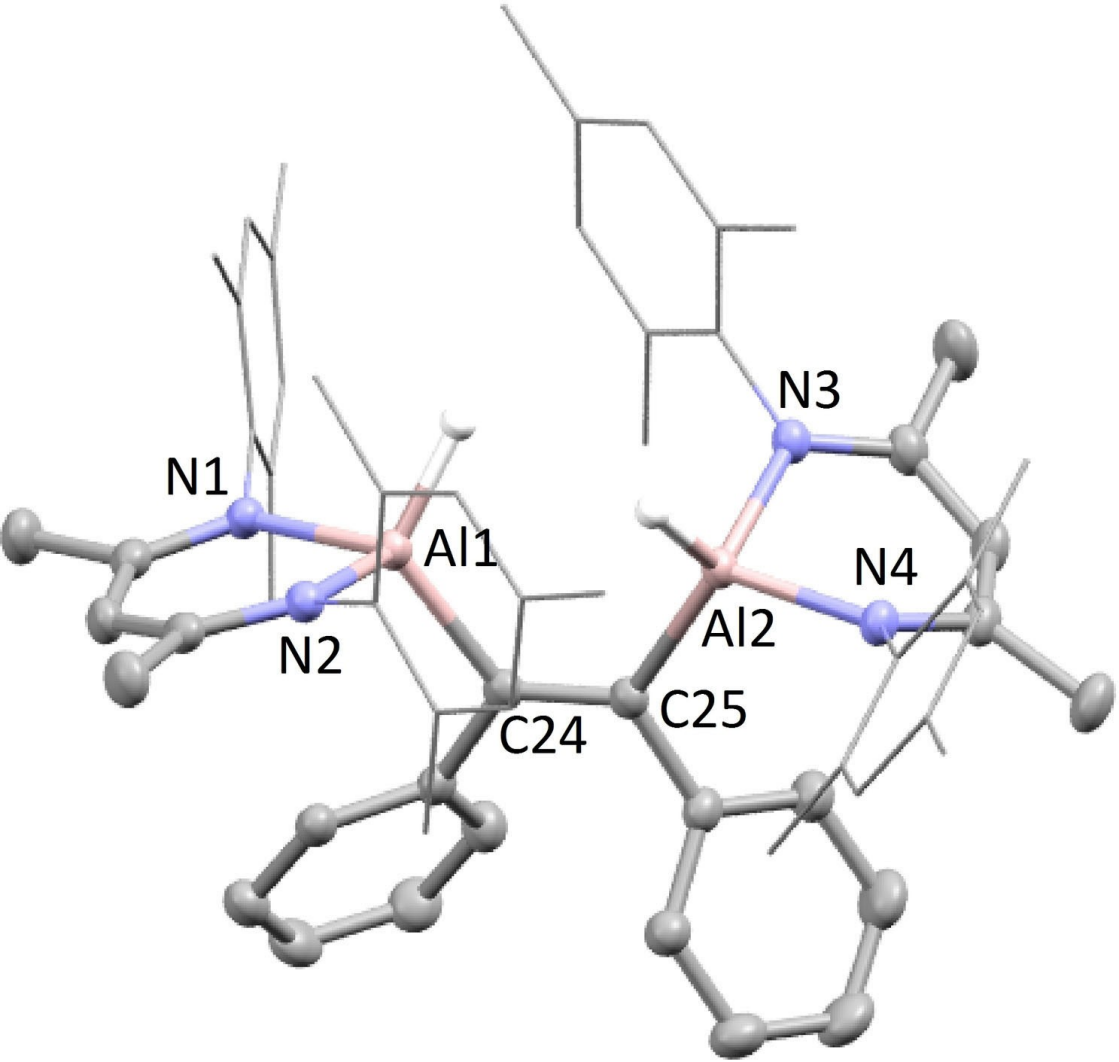
Solid‐state structure of compound **11**. Selected bond lengths [Å]: C24−C24 1.368(3), Al1−C24 2.005(2), Al2−C25 2.017(2), Al1−H 1.55(2), Al2−HA 1.52(2).

Reaction of **7** with an excess of C_6_F_6_ at 25 °C saw the slow formation of a new C−F bond activated product, as identified by characteristic ^19^F NMR signals (Scheme 4b). Heating the reaction for 30 minutes at 80 °C led to the complete consumption of **7**, and the formation of a mixture of two major products (**12 a**, **b**), in addition to a minor product (**12 c**) present in ≈10 % (Figure S16). ^19^F NMR peaks at −119.6, −153.6 and −161.8 ppm are proposed to correspond to **12 b** and exist alongside a broad doublet at −157.6 ppm, corresponding to **12 a**.^[35]^ A set of smaller peaks at −118.7, −152.6 ppm and a broad singlet at 162.3 ppm are consistent with the formation of **12 c** and match previously reported data for this compound.^[35]^ Whilst the major products **12 a**, **b** are likely to form from oxidative addition across the Al−Al bond of **7**, the formation for **12 c** provides evidence that competitive disproportionation also occurs, being consistent with C−F bond activation at a monomeric Al^I^ centre.[^32^, ^36^] Although H/F scrambling has previously been reported,^[35]^ in an independent sample containing **12 a** and **12 b**, the formation of **12 c** was not observed.

To conclude, we have been able to access a range of new low oxidation state Al compounds using **A** as a stoichiometric reducing agent. Monomeric Al^I^ species are proposed to be key intermediates in the equilibrium processes that determine product formation, with both DFT and reactivity studies providing early evidence supporting this theory and work to provide further support ongoing. These reactions highlight how careful tuning of the ligand environment can have a drastic effect on product formation, and have allowed us to access low oxidation state compounds not accessible through traditional reductive routes. Future efforts will focus on making such routes to low oxidation state Al compounds more scalable, with the aim of making Al reagents more synthetically viable.

## Conflict of interest

The authors declare no conflict of interest.

## Supporting information

As a service to our authors and readers, this journal provides supporting information supplied by the authors. Such materials are peer reviewed and may be re‐organized for online delivery, but are not copy‐edited or typeset. Technical support issues arising from supporting information (other than missing files) should be addressed to the authors.

Supporting Information

Supporting Information

## Data Availability

SCXRD data is available from https://www.ccdc.cam.ac.uk/products/csd/ with deposition numbers 2155210–2155214.
