# Supplementary material for: Exploring Equilibria between Aluminium(I) and Aluminium(III): The Formation of Dihydroalanes, Masked Dialumenes and Aluminium(I) Species
Source: Angew Chem Int Ed Engl. 2022 May 17;61(31):e202205901. doi: 10.1002/anie.202205901 (PMC9401008; doi:10.1002/anie.202205901)
Supplement: Supplementary file 1 — Supporting Information [file ANIE-61-0-s001.xyz]

1 C                  4.05864100    0.11640000   -1.76397400 H                  4.31977200    0.43289600   -2.77219700 C                 -5.03996400    0.25216200    0.73737900 N                 -1.09862400   -1.07042100    0.11097100 C                 -3.03786800   -1.72661000   -1.84015600 H                 -1.97475300   -1.54755000   -2.05290700 N                  1.10193800   -1.06502300    0.15654700 C                 -1.73593100    0.42799600    2.60506400 H                 -0.74240200    0.09591900    2.28305300 C                 -2.07703500   -0.35890600    3.87048100 C                 -4.70538800   -0.47347300   -0.40097400 H                 -5.47275200   -0.70353700   -1.13592700 C                  2.73540300   -0.24452500   -1.49346800 C                  3.04500200   -1.21411700    2.20674500 H                  1.98108800   -0.99172200    2.36824600 C                  3.40236500   -0.72080800    0.81993700 C                 -4.06106300    0.54184300    1.67666000 H                 -4.32493800    1.09567300    2.57583500 C                 -2.73681600    0.12738000    1.50619600 C                  1.73035900   -0.22485800   -2.62875400 H                  0.73780700   -0.46316200   -2.23002200 C                  3.83603600   -0.53475000    3.31846000 C                 -1.65198900    1.92501600    2.89897500 C                  4.70875900   -0.35622300    0.49831500 H                  5.47792700   -0.39752700    1.26556400 C                  1.64655400    1.15174500   -3.28603000 C                  2.41197800   -0.65645500   -0.18507400 C                 -3.39817900   -0.90493500   -0.61982300 C                  2.06361100   -1.30448700   -3.65838200 C                  5.03994600    0.06674600   -0.78454400 C                 -2.40960600   -0.59353600    0.34005400 C                  0.00176900   -0.30404500    0.04235000 C                  3.21201800   -2.73283600    2.28233100 C                 -3.83072700   -1.34970100   -3.08605400 C                 -3.19928900   -3.21675700   -1.53389400 C                  0.00114600    1.15749400   -0.13693500 C                  0.96245600    1.95477500    0.49684200 C                 -0.95842200    1.77399800   -0.94981100 C                  0.95616800    3.33214000    0.32438800 H                  1.70922700    1.49001800    1.13554700 C                 -0.95087100    3.15113100   -1.12380800 H                 -1.69985000    1.16550000   -1.46137300 H                  1.70626200    3.93893600    0.82634700 H                 -1.69457500    3.61448900   -1.76799300 C                  0.00097900    3.95543800   -0.48681500 C                 -0.01744800    5.44352700   -0.64820600 H                 -0.64098400    5.91526000    0.12000600 H                  0.98319100    5.87338100   -0.55428000 H                 -0.42687700    5.74074700   -1.61714000 Al                 0.00185900   -2.69986900    0.30802500 H                  0.11243000   -3.58888000   -0.99429000 H                 -0.10415600   -3.31257300    1.76095700 H                 -3.44936400   -1.88879100   -3.95836500 H                 -4.89092700   -1.60864600   -2.99113800 H                 -3.76996800   -0.27744400   -3.29824900 H                 -6.06250900    0.58500700    0.89242200 H                 -2.63632800   -3.51390800   -0.64236800 H                 -4.25184300   -3.45755700   -1.34566400 H                 -2.84998400   -3.83631500   -2.36593100 H                 -0.85728400    2.13539500    3.62247200 H                 -1.44746100    2.50394700    1.99287000 H                 -2.58926900    2.29750500    3.32746000 H                 -1.34484100   -0.16402600    4.66099300 H                 -3.06380200   -0.07649800    4.25476700 H                 -2.09075600   -1.43581200    3.67911700 H                  3.77149000    0.55632300    3.25611400 H                  3.45639300   -0.84013400    4.29807000 H                  4.89718100   -0.80545800    3.29070000 H                  2.64512300   -3.24509600    1.49764600 H                  4.26480300   -3.00969400    2.15483600 H                  2.86977200   -3.12505200    3.24529900 H                  0.84927400    1.17624800   -4.03637200 H                  1.44519300    1.93812900   -2.55181900 H                  2.58222200    1.40493400   -3.79711800 H                  1.32863100   -1.31006200   -4.47006700 H                  3.04976600   -1.13152900   -4.10417900 H                  2.07334700   -2.29918100   -3.20344800 H                  6.06179400    0.35163300   -1.019282002 C                 -3.49880800   -4.59394100    0.03332900 H                 -4.33527800   -5.10516300    0.50683400 C                 -3.20273700    4.86672000    0.25599900 N                 -2.15804200    0.81930300    0.63815600 C                 -3.60990700    2.03132700    2.78312500 H                 -3.66912400    0.97159500    2.49652300 N                 -1.80580000   -1.28621100    0.09756300 C                 -1.69727900    2.14970700   -1.94125400 H                 -1.53957100    1.09705600   -1.68597300 C                 -0.32591800    2.72608200   -2.27440700 C                 -3.51096300    4.16531400    1.41764600 H                 -3.99165100    4.68289100    2.24491800 C                 -3.22955500   -3.26933100    0.39028300 C                 -0.09991100   -2.61419000   -1.73898900 H                  0.37744200   -2.05425200   -0.91902200 C                 -1.31273500   -3.30504500   -1.14557300 C                 -2.62649800    4.19886100   -0.81712400 H                 -2.40345000    4.73964500   -1.73575100 C                 -2.31804100    2.83806800   -0.74417000 C                 -4.07924400   -2.62234900    1.46726000 H                 -3.79281700   -1.56964700    1.56222300 C                 -0.47298800   -1.58621600   -2.80682100 C                 -2.62460600    2.19562800   -3.15443500 C                 -1.62355000   -4.62587900   -1.46541500 H                 -0.99532300   -5.16506700   -2.16877200 C                 -5.56733900   -2.67085900    1.12477100 C                 -2.14388600   -2.61678100   -0.22963200 C                 -3.22356300    2.80528500    1.53667800 C                 -3.81029600   -3.27356700    2.82356100 C                 -2.71720200   -5.26814900   -0.89270300 C                 -2.59114900    2.15224200    0.45646000 C                 -2.63402800   -0.24216300   -0.03130300 C                  0.94389000   -3.57721900   -2.28274200 C                 -4.97480400    2.44487600    3.32712900 C                 -2.54703800    2.13226100    3.87511000 C                 -3.90029800   -0.24541600   -0.79157300 C                 -4.04251700   -0.98784600   -1.97178600 C                 -5.00095200    0.48752800   -0.32889900 C                 -5.24119400   -0.97506300   -2.67221400 H                 -3.20916100   -1.57637700   -2.34314900 C                 -6.19951000    0.48527300   -1.02922600 H                 -4.92179300    1.04295400    0.60054900 H                 -5.32986200   -1.55358900   -3.58906000 H                 -7.04529500    1.05136100   -0.64499000 C                 -6.34028200   -0.23738100   -2.21826700 C                 -7.62111600   -0.20879300   -2.99275600 H                 -7.60104100    0.57330100   -3.76057300 H                 -7.80309600   -1.15524800   -3.50908100 H                 -8.47950500   -0.00169000   -2.34845800 Al                -0.53387400   -0.20561800    1.25504000 H                 -0.82195900   -0.59780300    2.78061900 H                 -5.28337800    1.77408200    4.13483500 H                 -4.95913700    3.45840300    3.74288000 H                 -5.74900800    2.42082200    2.55268900 H                 -3.42834200    5.92730400    0.18303300 H                 -1.58930400    1.72476300    3.53959800 H                 -2.39205900    3.17696900    4.17169200 H                 -2.84861300    1.57076500    4.76563200 H                 -2.20900900    1.60755900   -3.98056500 H                 -3.61666400    1.79668200   -2.92017200 H                 -2.75458800    3.22326100   -3.51369800 H                  0.14820000    2.16123800   -3.08541500 H                 -0.39292900    3.77392300   -2.58936400 H                  0.34256600    2.69135600   -1.40776600 H                 -1.14995300   -0.82049200   -2.41719300 H                  0.42363800   -1.07345800   -3.17336800 H                 -0.96260000   -2.07262200   -3.65985500 H                  1.21440300   -4.34135600   -1.54742500 H                  0.59777000   -4.08445700   -3.19127600 H                  1.85642200   -3.03127600   -2.54229600 H                 -6.15238700   -2.09965000    1.85361300 H                 -5.76758500   -2.25740100    0.13136500 H                 -5.94491100   -3.69968000    1.13918800 H                 -4.41200100   -2.80404100    3.60905000 H                 -4.05912700   -4.34096500    2.80525500 H                 -2.75707000   -3.18054600    3.10498700 N                  3.05045600    1.70386600    0.50820400 C                  4.02215400    1.74290100    1.42803600 C                  4.43702500    0.62511600    2.16821100 H                  5.19513700    0.81001000    2.92027200 C                  4.14403600   -0.71906000    1.90840800 N                  3.22686000   -1.11234300    1.01680200 C                  4.76587800    3.02525300    1.68021100 H                  5.03159000    3.52079900    0.74216800 H                  5.67520900    2.84020200    2.25306000 H                  4.15300800    3.73859000    2.24039400 C                  4.95863500   -1.75090900    2.63389300 H                  4.30892300   -2.43402700    3.19078400 H                  5.65715200   -1.28552700    3.32988000 H                  5.52418000   -2.37646200    1.93582800 C                  2.76276400    2.91018500   -0.21863700 C                  3.18251300    3.02099400   -1.56305900 C                  2.86144900    4.18740000   -2.26114400 H                  3.17510200    4.28984000   -3.29589700 C                  2.14083800    5.21427900   -1.66477100 C                  1.73414100    5.08949800   -0.34533000 H                  1.15597700    5.88563800    0.11860100 C                  2.04359700    3.95144300    0.40463200 C                  3.99332800    1.92610000   -2.22973700 H                  3.61986000    0.96914100   -1.84785300 C                  1.54424700    3.86454900    1.83351300 H                  2.05282700    3.03213800    2.33389900 C                  3.31001000   -2.46981100    0.55483300 C                  2.64163800   -3.51271200    1.22297200 C                  2.81363700   -4.81899700    0.75565500 H                  2.29853100   -5.63170100    1.26403200 C                  3.62996900   -5.09567500   -0.33288100 C                  4.27407600   -4.05404300   -0.98794700 H                  4.91362500   -4.26674600   -1.84282600 C                  4.12569800   -2.73127300   -0.56606600 C                  1.73352100   -3.26468900    2.40960300 H                  1.84176900   -2.21282000    2.70736500 C                  4.88675300   -1.63285500   -1.28379700 H                  4.57760800   -0.67035800   -0.85938000 H                  1.89264000    6.10735800   -2.23198600 H                  3.75897800   -6.11941700   -0.67338200 C                  4.56151500   -1.59844400   -2.77417800 H                  5.15867800   -0.82924500   -3.27702700 H                  4.78361300   -2.55360700   -3.26359500 H                  3.50373400   -1.36640500   -2.94121700 C                  6.39227200   -1.77270100   -1.05726200 H                  6.64942700   -1.72552100    0.00610300 H                  6.76581000   -2.72728500   -1.44563500 H                  6.93902500   -0.97127900   -1.56561900 C                  0.28000000   -3.48359800    1.99976200 H                  0.09974200   -4.52183400    1.69365800 H                 -0.40828500   -3.23959000    2.81464800 H                  0.02045500   -2.85994200    1.13890800 C                  2.08106200   -4.13947800    3.61239200 H                  1.90967300   -5.19995100    3.39746800 H                  3.12773000   -4.03570100    3.91641700 H                  1.45434000   -3.87767600    4.47018400 C                  1.82844100    5.13551600    2.63263000 H                  2.88090900    5.43417000    2.58157000 H                  1.23424100    5.98063600    2.26962000 H                  1.56837500    4.99082100    3.68559300 C                  0.05293900    3.55172100    1.84956600 H                 -0.32931900    3.49786700    2.87337600 H                 -0.52852000    4.31023400    1.31178300 H                 -0.14688200    2.59248300    1.35968200 C                  5.47396500    2.01811600   -1.85712800 H                  5.89445100    2.99220100   -2.13420300 H                  5.63724200    1.87032200   -0.78425300 H                  6.04958000    1.24674800   -2.38259000 C                  3.82328500    1.89374400   -3.74375900 H                  4.26878200    2.76751300   -4.23240700 H                  4.31834100    1.01195900   -4.16040400 H                  2.76704200    1.84899000   -4.02842800 Al                 1.90561700    0.10333700    0.19903100 H                  1.94366100   -0.18860500   -1.37614700 H                 -2.94598800   -6.29710800   -1.156798003 Al                -1.07309300    0.36596900   -1.22318000 N                 -2.68100900    1.26890600   -0.44895200 C                 -3.26435300    0.08429800   -0.19729200 H                 -0.98197200    0.56161600   -2.80276900 N                 -2.53032100   -0.91120000   -0.71850900 C                 -4.49554900   -0.08791500    0.59821800 C                 -4.73948100    0.75376500    1.69266900 H                 -4.01888300    1.52750300    1.94391100 C                 -5.87727400    0.58980300    2.46998800 H                 -6.04176800    1.24452700    3.32281900 C                 -6.81551400   -0.40714900    2.17878300 C                 -6.56533900   -1.24635600    1.08840200 H                 -7.27650300   -2.03327400    0.84761200 C                 -5.42361700   -1.09744800    0.31162600 H                 -5.25331100   -1.76938700   -0.52416200 C                 -8.05912300   -0.55583900    2.99874600 H                 -7.87985500   -0.32249800    4.05186500 H                 -8.46497000   -1.56887800    2.93868200 H                 -8.84431200    0.12612500    2.65242800 C                 -3.32199100    2.52247500   -0.33617400 C                 -2.72958100    3.50737400    0.48659100 C                 -3.34209500    4.75394300    0.59864500 H                 -2.89296600    5.51077800    1.23734400 C                 -4.51828000    5.03943500   -0.08638500 H                 -4.98836000    6.01375000    0.01454000 C                 -5.07938400    4.07325300   -0.90911100 H                 -5.98699000    4.30055100   -1.46563200 C                 -4.50035000    2.80994800   -1.05737100 C                 -1.42225800    3.21030300    1.18327500 H                 -1.39115500    2.12740900    1.37013400 C                 -0.25721900    3.55472300    0.25310800 H                 -0.37702800    3.11513800   -0.74507800 H                  0.69930000    3.21087800    0.66263100 H                 -0.17775600    4.63950400    0.11086300 C                 -1.25029900    3.90416400    2.52789600 H                 -1.14315600    4.98996600    2.42231900 H                 -0.34580200    3.53871300    3.02536000 H                 -2.09963500    3.71647000    3.19240600 C                 -5.12270600    1.83320500   -2.03362400 H                 -4.60413100    0.87482100   -1.94253100 C                 -6.60111100    1.58466600   -1.74525900 H                 -6.76398000    1.28495500   -0.70520600 H                 -6.99169200    0.79054900   -2.39159700 H                 -7.20227200    2.48161400   -1.93265400 C                 -4.91216600    2.30466400   -3.47206500 H                 -5.38681400    3.27789900   -3.64232200 H                 -5.34663100    1.59338900   -4.18309700 H                 -3.84732200    2.40712800   -3.70288900 C                 -2.73490800   -2.28622100   -0.46496000 C                 -2.59170200   -2.82547300    0.82795700 C                 -2.81765800   -4.19377000    1.00401000 H                 -2.71143400   -4.62030900    1.99980100 C                 -3.15278700   -5.01460300   -0.06309000 H                 -3.32917600   -6.07484100    0.09660400 C                 -3.23162300   -4.47818000   -1.34479300 H                 -3.45665700   -5.13020000   -2.18462000 C                 -3.01366400   -3.11982200   -1.57195600 C                 -2.12037700   -2.00417400    2.01140700 H                 -2.06270900   -0.94868800    1.71435900 C                 -3.07860100   -2.09790400    3.19665600 H                 -4.09966100   -1.82530500    2.91118000 H                 -2.76180100   -1.43032200    4.00447600 H                 -3.10692300   -3.11417100    3.60597300 C                 -0.70771900   -2.43293300    2.41318600 H                 -0.68002700   -3.48433300    2.72336400 H                 -0.33532000   -1.82261900    3.24218700 H                  0.00082800   -2.33295300    1.58086700 C                 -3.07144000   -2.53665300   -2.97101700 H                 -2.31532600   -1.74044500   -3.01903500 C                 -4.42398200   -1.87939000   -3.24782800 H                 -5.23339100   -2.61801400   -3.19745000 H                 -4.44466100   -1.42622600   -4.24473400 H                 -4.64351900   -1.09019000   -2.52257300 C                 -2.74540500   -3.55066800   -4.06091700 H                 -1.80940100   -4.08190300   -3.85800800 H                 -2.64715100   -3.04905300   -5.02811800 H                 -3.53566400   -4.30206300   -4.17052600 Al                 0.87786700    0.32711700    0.53658800 N                  2.35700900   -0.98296100    0.28450200 C                  3.24423700   -0.00173900    0.04065900 H                  0.58366500    0.54192100    2.09678500 N                  2.63704600    1.19177800    0.12161900 C                  4.66128600   -0.22657200   -0.30236400 C                  5.03301000   -1.39198900   -0.98938500 H                  4.27299200   -2.11444700   -1.26720200 C                  6.35614800   -1.62269000   -1.33369300 H                  6.61785600   -2.53160600   -1.87119900 C                  7.36051700   -0.70612300   -1.00162600 C                  6.98689600    0.45550800   -0.31972300 H                  7.74926000    1.18323300   -0.05126300 C                  5.66193700    0.70119600    0.01827700 H                  5.40788200    1.61826600    0.53881100 C                  8.78513000   -0.95605100   -1.38710300 H                  8.93406600   -0.81991500   -2.46413300 H                  9.46851300   -0.27469200   -0.87481200 H                  9.09141700   -1.98059700   -1.15561400 C                  2.66399500   -2.28986300    0.73171000 C                  2.34143500   -3.38377600   -0.10101600 C                  2.51819100   -4.67623300    0.39114600 H                  2.26697900   -5.52564800   -0.23943800 C                  3.01814600   -4.89451300    1.67052100 H                  3.14985200   -5.90795800    2.03958400 C                  3.35675000   -3.81083000    2.46869900 H                  3.75968200   -3.98016400    3.46589400 C                  3.18607600   -2.49675300    2.02455900 C                  1.79475700   -3.14382200   -1.49333700 H                  2.16049100   -2.15901700   -1.81986200 C                  0.27195300   -3.06337500   -1.45767600 H                 -0.06333000   -2.30369500   -0.74326700 H                 -0.14128100   -2.80625000   -2.44114200 H                 -0.17312200   -4.01503500   -1.14181200 C                  2.25632800   -4.17771000   -2.51406800 H                  1.83179000   -5.16720200   -2.31267800 H                  1.93221900   -3.89179500   -3.51945800 H                  3.34637100   -4.28290500   -2.52689900 C                  3.56748900   -1.35158900    2.94050200 H                  3.28958300   -0.41107300    2.45168000 C                  5.07658200   -1.32854300    3.18588400 H                  5.63916600   -1.28331800    2.24745400 H                  5.36091300   -0.46281300    3.79450000 H                  5.39931800   -2.22889300    3.72166500 C                  2.80299500   -1.39975400    4.26110000 H                  3.03111700   -2.31078200    4.82599800 H                  3.07054100   -0.54596500    4.89277400 H                  1.72220600   -1.36933900    4.09512700 C                  3.16988800    2.41657300   -0.33377400 C                  3.47917500    2.62008800   -1.69323400 C                  4.00055500    3.85963300   -2.07482100 H                  4.24554700    4.02654200   -3.12233000 C                  4.19307400    4.87808400   -1.15306600 H                  4.59889700    5.83441400   -1.47137600 C                  3.84419900    4.67647800    0.17890200 H                  3.97617900    5.48471800    0.89314800 C                  3.32776600    3.45605100    0.61295800 C                  3.22108000    1.57449000   -2.76062000 H                  2.85390300    0.66052700   -2.27839100 C                  4.49234700    1.21712700   -3.52921100 H                  5.29796400    0.90268300   -2.85836800 H                  4.30209400    0.40320000   -4.23662900 H                  4.85720900    2.07336600   -4.10814200 C                  2.12470500    2.04953700   -3.71427700 H                  2.43823700    2.94971900   -4.25538900 H                  1.88816000    1.27935600   -4.45560600 H                  1.20124000    2.28853800   -3.17808300 C                  2.96914200    3.22868600    2.07157300 H                  2.09548200    2.56178200    2.09814000 C                  4.08784500    2.49796600    2.81519800 H                  5.01314900    3.08706700    2.80950200 H                  3.81020200    2.31950100    3.86004800 H                  4.30383700    1.52547900    2.36309600 C                  2.59377900    4.51000200    2.80583300 H                  1.82785500    5.07853500    2.26771800 H                  2.20341500    4.27663200    3.80117800 H                  3.45817900    5.16813400    2.949245004Al                -1.27828400    0.14987100   -1.37588500 Al                 1.25788100   -0.15233400   -1.35151500 N                 -2.44780000    1.21118900   -0.17860400 N                 -2.72724500   -0.93496300   -0.59079400 N                  2.43494900   -1.22124200   -0.16260900 N                  2.69388400    0.93317600   -0.54565200 C                 -3.16415800    0.11595600    0.13038600 C                 -4.23121400    0.04542100    1.14005700 C                 -2.49794300    2.44848800    0.50070800 C                 -2.14826800    2.57372200    1.86031900 C                  2.50234500   -2.46895300    0.49610300 C                  3.14638200   -0.12494500    0.15557400 C                 -1.59566900    1.42943900    2.68882700 H                 -1.59990200    0.51674800    2.07903600 C                 -3.47512100   -2.11363800   -0.82831600 C                  3.44369600    2.10666300   -0.80604900 C                 -6.22790100   -0.13063500    3.12713400 C                 -5.11198200    1.11239300    1.36287900 H                 -5.02875900    2.01755300    0.76730900 C                 -6.09625500    1.01742000    2.33823300 H                 -6.77579600    1.85201200    2.49524400 C                  2.88834500    3.36284900   -0.48192700 C                 -2.86916000    3.58680400   -0.25520400 C                 -0.14333700    1.71691000    3.07916200 H                  0.51234900    1.75453600    2.20209300 H                  0.24480500    0.94729700    3.75425300 H                 -0.05818000    2.67882600    3.59744600 C                  2.88287600   -3.59126600   -0.27965200 C                  4.22052700   -0.05921300    1.15766600 C                 -2.97908900    4.81611600    0.39246500 H                 -3.27219300    5.69370500   -0.17761100 C                  1.41076000   -0.27983800   -3.38127600 H                  2.42185800   -0.50784000   -3.73905300 C                 -5.33512200   -1.18678900    2.90892800 H                 -5.40944100   -2.08274400    3.52109700 C                  2.16375000   -2.61994700    1.85594200 C                 -4.35617300   -1.10666300    1.92942100 H                 -3.66367400   -1.93161300    1.78062300 C                 -4.75979600   -2.05303800   -1.41469400 C                  5.10002500   -1.12791500    1.37964200 H                  5.01421800   -2.03426900    0.78678200 C                 -2.89278000   -3.36373100   -0.52678700 C                  4.70314400    2.03508400   -1.44541300 C                  3.00439500   -4.83045300    0.34678100 H                  3.30283800   -5.69582300   -0.23902300 C                  1.49702500    3.50075400    0.09570400 H                  1.14588200    2.49791000    0.37203000 C                  3.62543000    4.51743900   -0.74967600 H                  3.19446200    5.48462700   -0.49866700 C                  4.35139300    1.09314600    1.94595100 H                  3.66053000    1.91931600    1.79794200 C                 -2.43850000    1.16816400    3.93694300 H                 -2.37158300    2.00668600    4.63972500 H                 -2.08419300    0.27427500    4.46260800 H                 -3.49505600    1.02042600    3.69461300 C                 -2.27375400    3.83018100    2.46308800 H                 -2.01479100    3.93424600    3.51543200 C                 -2.70280900    4.93993100    1.75038300 H                 -2.79792400    5.90386200    2.24248800 C                  1.59538400   -1.49749300    2.70278200 H                  1.58123300   -0.57681500    2.10570500 C                  0.97067300    1.11156700   -3.72798100 H                  1.72438000    1.86404800   -3.95627200 C                  6.08797700   -1.03345400    2.35120100 H                  6.76610800   -1.86946000    2.50678500 C                 -1.37655800    0.39709000   -3.39683100 H                 -2.37179300    0.66597200   -3.77043600 C                 -0.34133800    1.42816100   -3.72821100 H                 -0.66496800    2.43964600   -3.97181700 C                  2.31179100   -3.88303000    2.43944100 H                  2.06421400   -4.00561700    3.49248300 C                  6.22514600    0.11515200    3.13833700 C                  4.88614500    4.45542000   -1.32709100 H                  5.44943100    5.36450400   -1.51870800 C                 -1.47396400   -3.47069600   -0.01440600 H                 -1.16977800   -2.47570000    0.33790500 C                  0.39110100   -1.26539400   -3.86245700 H                  0.72699900   -2.24495700   -4.20039000 C                  5.40700000    3.21838000   -1.67867800 H                  6.37869500    3.16064900   -2.16621000 C                  5.33379100    1.17277600    2.92182900 H                  5.41193100    2.06952700    3.53239400 C                 -5.38301800   -0.76096300   -1.89966600 H                 -4.74103600    0.06937200   -1.59297100 C                 -0.91875900   -0.94841800   -3.87735200 H                 -1.66281500   -1.66741600   -4.21650600 C                  2.74333200   -4.97769800    1.70538500 H                  2.85229400   -5.94820000    2.18144100 C                  5.29432200    0.73958100   -1.96062200 H                  4.62969800   -0.08118900   -1.67498900 C                  2.43664800   -1.23700500    3.95187000 H                  2.39273400   -2.08782800    4.64155000 H                  2.06347300   -0.36037000    4.49327000 H                  3.48832100   -1.06002000    3.70775600 C                 -5.45676700   -3.24434900   -1.62775800 H                 -6.44690200   -3.19723100   -2.07774500 C                  1.45790500    4.37462600    1.34658800 H                  2.12548400    3.99929200    2.13017100 H                  0.44149300    4.40807500    1.75483900 H                  1.75474400    5.40634600    1.12752900 C                 -3.13762200    3.45986900   -1.74223500 H                 -2.45852700    2.68524900   -2.12448200 C                  0.14929800   -1.81832800    3.09124000 H                 -0.50502900   -1.86186700    2.21355100 H                 -0.25531600   -1.06325700    3.77330300 H                  0.08432700   -2.78702200    3.59991400 C                 -7.30556600   -0.23652600    4.16072100 H                 -7.61869000    0.74647500    4.52174200 H                 -6.98390100   -0.82910500    5.02144200 H                 -8.19679200   -0.72748200    3.75300600 C                  3.16528200   -3.43500800   -1.76170800 H                  2.49951600   -2.64494600   -2.13585400 C                 -3.62549400   -4.52616500   -0.76969400 H                 -3.17662200   -5.48934200   -0.53707300 C                  0.54513700    4.04432700   -0.96846400 H                  0.90394700    5.00491900   -1.35783300 H                 -0.45157000    4.20492300   -0.54763600 H                  0.45635500    3.35357200   -1.81614900 C                 -6.76977900   -0.52206700   -1.30671000 H                 -7.49276500   -1.25676800   -1.67881000 H                 -7.14370400    0.47019200   -1.58367500 H                 -6.76047800   -0.58809900   -0.21453100 C                 -4.90739500   -4.47586500   -1.30116600 H                 -5.46655200   -5.39097400   -1.47552700 C                  6.67605900    0.45783900   -1.37316600 H                  7.41140000    1.18802500   -1.72993400 H                  7.03147200   -0.53448500   -1.67363600 H                  6.67059000    0.49990400   -0.27994100 C                 -5.43344800   -0.73378200   -3.42741500 H                 -4.43753200   -0.86288000   -3.86302000 H                 -5.84224700    0.21640000   -3.78852100 H                 -6.06962500   -1.53798000   -3.81430800 C                  5.35236200    0.74645900   -3.48854800 H                  4.36522600    0.92028300   -3.92804100 H                  5.73076600   -0.20861600   -3.86924900 H                  6.01942600    1.53559900   -3.85371300 C                 -1.31985400   -4.44523200    1.14889500 H                 -1.99225800   -4.20092400    1.97866500 H                 -0.29186800   -4.42226800    1.52751900 H                 -1.52894300   -5.47727400    0.84641500 C                 -2.85464700    4.73439100   -2.52791000 H                 -3.57632900    5.52698000   -2.30025100 H                 -2.92861700    4.53661800   -3.60181500 H                 -1.85228000    5.12506200   -2.32620400 C                 -0.54220900   -3.85268500   -1.16371600 H                 -0.84130500   -4.81309000   -1.60143700 H                  0.48702000   -3.95284700   -0.80653200 H                 -0.55902400   -3.10414700   -1.96566000 C                  7.30656900    0.21984000    4.16803000 H                  6.98970600    0.81537000    5.02849500 H                  8.19795700    0.70704600    3.75618600 H                  7.61782000   -0.76335900    4.53014500 C                 -4.56580500    2.98229500   -2.00273400 H                 -4.78077900    2.05147200   -1.47015700 H                 -4.73564100    2.80332300   -3.07028900 H                 -5.29135500    3.73358100   -1.66803300 C                  2.88064600   -4.69021800   -2.57733900 H                  3.58929100   -5.49549900   -2.35344600 H                  2.97523200   -4.47278300   -3.64569300 H                  1.87075800   -5.07316000   -2.40017000 C                  4.60184800   -2.96712800   -1.99309700 H                  4.82456300   -2.05844000   -1.42660600 H                  4.78363800   -2.75365700   -3.05248900 H                  5.31453400   -3.73893500   -1.67800300AmAl(I) C                  3.92595600    1.68606600    1.17160100 H                  4.11844300    2.43299400    1.93681600 C                 -5.05479200   -0.59288100   -0.28647000 N                 -1.06398700    0.53543200   -0.95303600 C                 -2.83203200    2.50531900   -0.01665500 H                 -1.80317100    2.43392500    0.36832900 N                  1.14441200    0.46244100   -0.94390200 C                 -1.84238100   -2.30909100   -1.37466600 H                 -0.82969700   -1.89094700   -1.43386400 C                 -2.21162400   -2.79786700   -2.77475400 C                 -4.63607200    0.71607900   -0.06473800 H                 -5.35427200    1.45785900    0.27516500 C                  2.64012200    1.56095600    0.64416700 C                  3.21373000   -1.28317200   -1.87562700 H                  2.14620600   -1.54642600   -1.82879000 C                  3.46482500   -0.25024100   -0.79713700 C                 -4.13607900   -1.54577600   -0.70094500 H                 -4.46268300   -2.57037000   -0.87117100 C                 -2.79182800   -1.22229000   -0.91068100 C                  1.52382500    2.50044700    1.06467000 H                  0.60260800    1.91292200    1.17480000 C                  4.01756600   -2.56487500   -1.68411000 C                 -1.79790900   -3.47156800   -0.38371300 C                  4.73602700   -0.07773900   -0.24735800 H                  5.55358100   -0.70968400   -0.58625300 C                  1.76192100    3.17802200    2.40824400 C                  2.41475000    0.57487200   -0.34526500 C                 -3.30771900    1.08722900   -0.26087100 C                  1.26041200    3.55275100   -0.01410700 C                  4.96946000    0.87845000    0.73235600 C                 -2.38333900    0.10818500   -0.69343800 C                  0.02298200    0.12565900   -0.28981800 C                  3.47484000   -0.68784900   -3.25973700 C                 -3.66016100    3.25880900    1.01654200 C                 -2.76947000    3.29343300   -1.32554900 C                  0.00065400   -0.64989700    0.96658100 C                  0.92809900   -1.67883800    1.16980900 C                 -0.94626700   -0.38720300    1.96435800 C                  0.89623000   -2.43460300    2.33352300 H                  1.66344400   -1.89327400    0.39874600 C                 -0.95995900   -1.13701300    3.13241000 H                 -1.66525100    0.41634600    1.82493000 H                  1.61717100   -3.23706700    2.47307100 H                 -1.69361100   -0.91497100    3.90404000 C                 -0.04687600   -2.17845200    3.33483100 C                 -0.09496500   -3.01149300    4.57776600 H                 -0.76701400   -3.86833900    4.45248500 H                  0.88882800   -3.41286100    4.83449500 H                 -0.46426300   -2.44075000    5.43385600 Al                 0.08984000    1.09251400   -2.61808400 H                 -3.19176700    4.21967400    1.25123900 H                 -4.66925400    3.47832500    0.65058700 H                 -3.76161300    2.69506500    1.94961600 H                 -6.09457000   -0.86698200   -0.13114700 H                 -2.13213400    2.80008200   -2.06888300 H                 -3.76841400    3.38956300   -1.76551100 H                 -2.37473600    4.30223000   -1.16280700 H                 -1.04249000   -4.20743900   -0.67913000 H                 -1.55942700   -3.12785400    0.62770800 H                 -2.76178400   -3.99109000   -0.34060300 H                 -1.51066900   -3.56456400   -3.12098500 H                 -3.21500300   -3.23825300   -2.78496000 H                 -2.20576900   -1.97917400   -3.50099200 H                  3.88710000   -2.98367600   -0.68083000 H                  3.70867200   -3.32288400   -2.41026200 H                  5.08988200   -2.39952100   -1.83448100 H                  2.86351500    0.20376300   -3.43891800 H                  4.52516500   -0.39064200   -3.35846900 H                  3.25301300   -1.41077800   -4.05205000 H                  0.87478200    3.74417400    2.70754200 H                  1.98062500    2.45102000    3.19614900 H                  2.59512400    3.88808300    2.36565300 H                  0.43226700    4.21088800    0.27199500 H                  2.14753900    4.17495400   -0.17462000 H                  1.00565200    3.09882700   -0.98076200 H                  5.96352600    0.99648700    1.15460200AmAl(I)-d C                 -3.43694200   -3.47363700   -2.87676000 H                 -3.75865700   -3.57514400   -3.91201100 C                 -3.99737400    4.42919800    2.57306600 N                 -2.77155600    1.31552600    0.02193900 C                 -3.41740900    3.83795100   -1.15299900 H                 -3.60333400    2.87020300   -1.64299100 N                 -2.52547100   -0.82149300   -0.44189000 C                 -2.88123200    0.80934800    2.92156900 H                 -2.48915100    0.14230200    2.14347100 C                 -1.76980200    0.97430600    3.95731000 C                 -3.91940000    4.63575700    1.20001600 H                 -4.17580900    5.60899100    0.78865200 C                 -3.26968000   -2.19159900   -2.34270900 C                 -2.24810000   -3.05768400    1.24715200 H                 -2.76144100   -2.15501200    1.61196600 C                 -2.63913000   -3.22528600   -0.20732300 C                 -3.66831400    3.18754600    3.09866600 H                 -3.72044000    3.02694100    4.17421600 C                 -3.25824300    2.13247300    2.27946900 C                 -3.56152400   -0.98451500   -3.21147300 H                 -3.37618600   -0.07596700   -2.62306200 C                 -2.68134600   -4.22027700    2.13119500 C                 -4.09812900    0.13546000    3.55793700 C                 -2.81920100   -4.48125600   -0.78282900 H                 -2.66494300   -5.37299100   -0.18070800 C                 -5.02958200   -0.95872400   -3.63656600 C                 -2.84630000   -2.07855500   -1.00619800 C                 -3.52913300    3.61062900    0.33952100 C                 -2.63549100   -0.93141900   -4.42476300 C                 -3.20391800   -4.60938700   -2.11499100 C                 -3.20530600    2.34939600    0.88517700 C                 -3.40493200    0.13161800   -0.10224000 C                 -0.75025400   -2.80520200    1.39580500 C                 -4.42559100    4.84183000   -1.69864600 C                 -1.99150700    4.25126500   -1.51201800 C                 -4.85820100   -0.05094800    0.02529200 C                 -5.41625700   -1.26381500    0.45529500 C                 -5.72481600    0.99833700   -0.31325400 C                 -6.79276400   -1.41316100    0.54751400 H                 -4.76759500   -2.08869800    0.73194000 C                 -7.09981200    0.83632500   -0.22804400 H                 -5.31066300    1.93775700   -0.66628000 H                 -7.20631000   -2.35989200    0.88780900 H                 -7.75471700    1.65846400   -0.50841000 C                 -7.66047600   -0.36882500    0.20902300 C                 -9.14328900   -0.52831900    0.33542000 H                 -9.48619900   -0.24664900    1.33778800 H                 -9.45474800   -1.56346200    0.17214100 H                 -9.68081800    0.10572700   -0.37453100 Al                -1.07268600    0.58207100   -0.75483900 H                 -4.38203700    4.87243500   -2.79156000 H                 -4.22208000    5.85816200   -1.34412500 H                 -5.45093700    4.59055500   -1.40751600 H                 -4.31040900    5.23609400    3.22995300 H                 -1.26347600    3.51880500   -1.14754300 H                 -1.73872100    5.21555300   -1.05591200 H                 -1.85827000    4.34268300   -2.59571800 H                 -3.83975100   -0.86030300    3.93724100 H                 -4.92502200    0.02565600    2.84980600 H                 -4.46709900    0.72468300    4.40575700 H                 -1.48144800   -0.00221300    4.36387000 H                 -2.09394800    1.59637100    4.79944000 H                 -0.87700900    1.43227900    3.51924100 H                 -3.74498100   -4.45230400    2.01165900 H                 -2.50381400   -3.98117800    3.18431000 H                 -2.11542200   -5.13242200    1.91167900 H                 -0.43026400   -1.92403000    0.82618600 H                 -0.16547200   -3.65905200    1.03346900 H                 -0.48324000   -2.63650500    2.44496000 H                 -5.25494100   -0.05105700   -4.20626200 H                 -5.69867000   -0.99149800   -2.77072100 H                 -5.27084500   -1.81651300   -4.27477200 H                 -2.84319000   -0.04726900   -5.03648300 H                 -2.76561700   -1.81304900   -5.06271200 H                 -1.58460000   -0.89257100   -4.12090700 C                  2.29942400   -4.36588400   -0.17379300 H                  2.15366400   -5.03899300   -1.01703100 C                  5.32944700    4.67077000   -0.57548600 N                  2.83771500    1.29925600   -0.18931100 C                  2.65682700    2.82755800   -2.58257100 H                  2.50853400    1.73900800   -2.53339000 N                  2.36799500   -0.72723400    0.54673100 C                  4.71360600    1.93824800    1.99631800 H                  4.14457800    1.01154200    1.87310900 C                  4.08291900    2.68047200    3.17394600 C                  4.44978200    4.32270200   -1.59484700 H                  4.39990300    4.93366300   -2.49286300 C                  2.26895200   -2.98633900   -0.39787300 C                  2.92978300   -1.70192400    3.16177100 H                  3.37887200   -0.78926400    2.74278700 C                  2.67988300   -2.64351600    1.99873300 C                  5.40334400    3.88309200    0.56466500 H                  6.09142900    4.15370800    1.36360300 C                  4.60739800    2.74438200    0.71629700 C                  2.01148700   -2.46815000   -1.79839100 H                  2.04480400   -1.37121900   -1.77518000 C                  3.90967200   -2.25515000    4.19076000 C                  6.15367900    1.53620900    2.30639200 C                  2.68947300   -4.02672400    2.17356100 H                  2.86360100   -4.43816000    3.16494900 C                  3.08430500   -2.94707300   -2.77524800 C                  2.45386000   -2.12752600    0.70158300 C                  3.63474800    3.19652900   -1.48842200 C                  0.61551300   -2.86847600   -2.27477100 C                  2.49876100   -4.88804900    1.09706500 C                  3.71070200    2.40857700   -0.32067300 C                  3.29276000    0.04139700   -0.03962100 C                  1.62477600   -1.28229900    3.83723300 C                  3.14425900    3.17560500   -3.98372100 C                  1.30143400    3.47232600   -2.30357000 C                  4.62552900   -0.40964400   -0.47406500 C                  5.37797800   -1.31792800    0.28031600 C                  5.15558000    0.06193400   -1.68310000 C                  6.62732100   -1.73386900   -0.16252600 H                  4.99025900   -1.68533200    1.22614800 C                  6.39810100   -0.36847700   -2.12329200 H                  4.57236200    0.75464900   -2.28441800 H                  7.20310400   -2.43367200    0.43903300 H                  6.78879300   -0.00139800   -3.06983200 C                  7.15955700   -1.27003100   -1.36935600 C                  8.51349600   -1.70618500   -1.83637100 H                  9.26330800   -0.92624200   -1.66127800 H                  8.85207100   -2.60429800   -1.31402500 H                  8.52257100   -1.91478700   -2.91015700 Al                 1.10416300    0.85200600    0.77439500 H                  2.45659100    2.77795900   -4.73616600 H                  3.19695700    4.25852100   -4.14170300 H                  4.13939100    2.76485300   -4.18419900 H                  5.95731700    5.55224600   -0.67270000 H                  0.94461000    3.21459100   -1.30043800 H                  1.36916100    4.56584500   -2.35681400 H                  0.54370900    3.14015700   -3.02130800 H                  6.18636300    0.84831600    3.15931500 H                  6.62317900    1.03834300    1.45190400 H                  6.76844400    2.40525500    2.56616000 H                  4.11267100    2.06532500    4.08121700 H                  4.61585900    3.61497600    3.38419600 H                  3.03638700    2.93343100    2.97392800 H                  4.84301000   -2.59255500    3.72771700 H                  4.15808900   -1.48547800    4.92861300 H                  3.49000000   -3.10259900    4.74385900 H                  0.92553200   -0.83421700    3.12202500 H                  1.12707000   -2.14730300    4.29314500 H                  1.80775200   -0.54248900    4.62524400 H                  2.93344100   -2.50537700   -3.76579700 H                  4.08934200   -2.67968700   -2.43334100 H                  3.05398100   -4.03623100   -2.89498100 H                  0.41760400   -2.48568100   -3.28183100 H                  0.49873900   -3.95846000   -2.30409200 H                 -0.16501400   -2.47907800   -1.61107800 H                  2.51334700   -5.96371100    1.25026900 H                 -3.33452100   -5.59574300   -2.55178100B N                 -1.41090200   -0.39411100    0.41319000 C                 -1.23358700   -0.51241000    1.73176500 C                  0.03151100   -0.62291800    2.32798700 H                  0.04366200   -0.75531700    3.40360600 C                  1.27911000   -0.42040800    1.71549700 N                  1.42669800   -0.26194400    0.39702800 C                 -2.43322300   -0.48456000    2.63059800 H                 -2.94578300    0.48101800    2.56273000 H                 -2.15828800   -0.65896500    3.67112000 H                 -3.16823100   -1.23586700    2.32305700 C                  2.48604500   -0.35906300    2.60486700 H                  3.11276500   -1.24606300    2.46043700 H                  2.20269800   -0.30892700    3.65671300 H                  3.11577100    0.50204900    2.36238000 C                 -2.70696800   -0.05955400   -0.09822700 C                 -3.05393000    1.30049600   -0.22434500 C                 -4.29961900    1.61822000   -0.76898100 H                 -4.57912400    2.66528100   -0.87194700 C                 -5.17832600    0.62607100   -1.18569400 C                 -4.81460300   -0.70835700   -1.06470300 H                 -5.49771600   -1.48711400   -1.39892800 C                 -3.58067500   -1.07796600   -0.52287600 C                 -2.11687700    2.41640800    0.19642500 H                 -1.20965500    1.96222600    0.61554900 C                 -3.21652100   -2.54616400   -0.42682500 H                 -2.24713600   -2.62353100    0.08018800 C                  2.71262000    0.06679100   -0.14433500 C                  3.67430800   -0.93953100   -0.35655500 C                  4.90307200   -0.57163400   -0.91224500 H                  5.65429700   -1.33946200   -1.08608600 C                  5.17324000    0.74509000   -1.25373500 C                  4.20117100    1.72093300   -1.06476600 H                  4.41217500    2.74613200   -1.35525600 C                  2.95517100    1.40635800   -0.51951000 C                  3.40459700   -2.40229100   -0.06054700 H                  2.46037000   -2.47437100    0.49316900 C                  1.89511100    2.47483900   -0.32158400 H                  0.92069500    2.00222800   -0.50690000 H                 -6.14262000    0.89333100   -1.60897500 H                  6.13577000    1.01192500   -1.68139100 C                  2.02765800    3.63129300   -1.30468700 H                  1.17243700    4.30684800   -1.21212200 H                  2.92620800    4.23000900   -1.11816500 H                  2.06539100    3.27797700   -2.33964400 C                  1.87607000    2.99616600    1.11647400 H                  1.62853600    2.20942100    1.83559900 H                  2.84919600    3.41693400    1.39579400 H                  1.12716900    3.78860100    1.22944700 C                  3.23249600   -3.18549900   -1.36283100 H                  4.15047300   -3.14862000   -1.96083200 H                  3.00896200   -4.23747700   -1.15787000 H                  2.41687300   -2.78292600   -1.96943300 C                  4.50497900   -3.03491800    0.79038200 H                  5.45380600   -3.08300800    0.24496100 H                  4.69206900   -2.47652300    1.71353100 H                  4.23902600   -4.06044300    1.06438600 C                 -4.24146000   -3.33238200    0.38923500 H                 -4.37378200   -2.91823200    1.39418900 H                 -5.22424900   -3.32969500   -0.09535200 H                 -3.93346000   -4.37726600    0.49495600 C                 -3.05999300   -3.15827600   -1.81834700 H                 -2.75579700   -4.20756200   -1.75004700 H                 -4.00541600   -3.11976500   -2.37198000 H                 -2.30476900   -2.63049200   -2.40763600 C                 -2.73956000    3.29884400    1.27743800 H                 -3.63155800    3.81499300    0.90477800 H                 -3.04083000    2.72150900    2.15753900 H                 -2.03145200    4.06597400    1.60762900 C                 -1.69588000    3.26006600   -1.00586700 H                 -2.56416100    3.72602000   -1.48565000 H                 -1.02243800    4.06618600   -0.69375900 H                 -1.17931100    2.65502800   -1.75793600 Al                 0.01291500   -0.66037500   -0.87625300 H                  0.08349800   -2.19018800   -1.30113800 H                 -0.05016100    0.45150900   -2.005011005 N                  1.42703800   -0.49005600    0.04574600 C                  1.25862900   -1.80846500    0.17602800 C                  0.00000000   -2.42469300    0.23530000 H                  0.00000000   -3.50314800    0.34207600 C                 -1.25862900   -1.80846500    0.17602700 N                 -1.42703800   -0.49005600    0.04574400 C                  2.47214300   -2.68720000    0.26599800 H                  3.10254800   -2.57691100   -0.62244700 H                  2.19722200   -3.73697700    0.37188900 H                  3.10046000   -2.40083600    1.11576800 C                 -2.47214300   -2.68720100    0.26599700 H                 -3.10045500   -2.40084100    1.11577200 H                 -2.19722100   -3.73697800    0.37188200 H                 -3.10255200   -2.57690700   -0.62244500 C                  2.75452200    0.04796100   -0.00511900 C                  3.38740000    0.20677400   -1.24837400 C                  4.66274000    0.77238400   -1.27637100 H                  5.15993700    0.89403700   -2.23812400 C                  5.31329100    1.18590000   -0.11311400 C                  4.65585500    1.01388600    1.10589000 H                  5.14758900    1.32641400    2.02648700 C                  3.38044300    0.45353000    1.18462700 C                 -2.75452200    0.04796100   -0.00512000 C                 -3.38044300    0.45352800    1.18462700 C                 -4.65585500    1.01388400    1.10589000 H                 -5.14759000    1.32641100    2.02648700 C                 -5.31329100    1.18590000   -0.11311400 C                 -4.66274000    0.77238500   -1.27637100 H                 -5.15993600    0.89404000   -2.23812400 C                 -3.38740000    0.20677500   -1.24837500 Al                 0.00000000    0.82102600   -0.09135100 H                 -0.00000200    1.78405500    1.17213400 H                  0.00000100    1.50120200   -1.52699000 C                  2.70601000   -0.23131800   -2.50815200 H                  2.46995000   -1.30219600   -2.49643500 H                  1.75181600    0.28961300   -2.64694800 H                  3.33152800   -0.03735600   -3.38259800 C                  6.66802300    1.82386600   -0.17362500 H                  7.24411100    1.47056800   -1.03336700 H                  6.59209600    2.91350500   -0.26705500 H                  7.25119500    1.62291600    0.72946300 C                  2.69086500    0.27744300    2.50247700 H                  1.73938900    0.82080400    2.52946000 H                  2.44778100   -0.77316000    2.70174300 H                  3.31354700    0.63777900    3.32464000 C                 -2.69086500    0.27743900    2.50247600 H                 -2.44777900   -0.77316400    2.70174000 H                 -1.73939100    0.82080200    2.52946200 H                 -3.31354900    0.63777100    3.32464000 C                 -6.66802200    1.82386800   -0.17362300 H                 -7.25120000    1.62290400    0.72945800 H                 -6.59209400    2.91350800   -0.26703500 H                 -7.24410500    1.47058300   -1.03337500 C                 -2.70601000   -0.23131600   -2.50815400 H                 -1.75181600    0.28961300   -2.64694800 H                 -2.46995100   -1.30219500   -2.49643800 H                 -3.33152700   -0.03735300   -3.38259900 mesBDIAlH2AlBDIdipp N                 -0.15064900   -2.78712900   -0.58693500 C                  0.10409600   -3.95301300    0.01749600 C                  1.19995500   -4.16551200    0.86354600 H                  1.26838700   -5.14513200    1.32196900 C                  2.28988200   -3.30730700    1.06943400 N                  2.35458300   -2.06704500    0.58133000 C                 -0.78555100   -5.13224600   -0.25811200 H                 -1.83900700   -4.90065000   -0.07611900 H                 -0.50185100   -5.98952300    0.35332000 H                 -0.71985400   -5.42407700   -1.31204500 C                  3.45896300   -3.85650100    1.83626300 H                  4.36057500   -3.87067700    1.21469100 H                  3.26137100   -4.87076700    2.18485500 C                 -1.22407400   -2.75021800   -1.53606400 C                 -2.52500400   -2.46198200   -1.09542700 C                 -3.55618000   -2.42691900   -2.03627000 H                 -4.56662400   -2.19811800   -1.69729000 C                 -3.32665400   -2.67614800   -3.38981600 C                 -2.02487500   -2.97929300   -3.79233200 H                 -1.82673300   -3.18844200   -4.84325700 C                 -0.96055100   -3.01835200   -2.88998000 C                  3.60210100   -1.36651600    0.68574900 C                  4.50953000   -1.46525500   -0.38717100 C                  5.71293000   -0.76779500   -0.30965300 H                  6.41464500   -0.84158600   -1.14018200 C                  6.04052900    0.02057300    0.79619500 C                  5.13121100    0.08246100    1.84903300 H                  5.36700600    0.69025100    2.72226200 C                  3.91101000   -0.59783000    1.81874100 N                  0.01099500    2.63663100   -0.35420800 C                 -0.34796600    3.80290400    0.17264600 C                 -0.95768600    3.89623800    1.44113600 H                 -1.11114500    4.90022700    1.82206900 C                 -1.58283000    2.86807400    2.15388800 N                 -1.46911100    1.56346300    1.83435500 C                 -0.11827700    5.08305700   -0.57829700 H                  0.91550500    5.42078100   -0.44066500 H                 -0.77769900    5.87601700   -0.22165100 H                 -0.26317000    4.95031500   -1.65359900 C                 -2.45289400    3.27490600    3.31064500 H                 -3.51501600    3.25543800    3.04862800 H                 -2.20950900    4.28804700    3.63485600 H                 -2.33245800    2.58749800    4.15329700 C                  0.55595700    2.56707000   -1.67455300 C                  1.90031800    2.91016400   -1.90742100 C                  2.40804100    2.75469000   -3.20046500 H                  3.44937800    3.00753200   -3.39078500 C                  1.61655800    2.27330800   -4.23261600 C                  0.29184300    1.93260100   -3.98371600 H                 -0.32269100    1.54824200   -4.79301400 C                 -0.25970000    2.06727900   -2.70951200 C                  2.82370900    3.37525300   -0.80024100 H                  2.22467900    3.56015100    0.09965200 C                 -1.71407200    1.73581100   -2.44457400 H                 -1.77618600    1.32268000   -1.42497100 C                 -2.46359300    0.68365300    2.35463900 C                 -3.79651000    0.79167400    1.89192900 C                 -4.77655000   -0.01808400    2.46773500 H                 -5.80334500    0.06739800    2.11580300 C                 -4.46361300   -0.94499500    3.45376300 C                 -3.14132100   -1.09280900    3.84626400 H                 -2.88215600   -1.83921500    4.59499600 C                 -2.12553900   -0.29642900    3.30949900 C                 -4.18689100    1.66115900    0.70879900 H                 -3.32579300    2.27720300    0.42198700 C                 -0.70117500   -0.54806500    3.76166400 H                 -0.03786800    0.15422600    3.23802500 H                  2.03091300    2.15643700   -5.23036100 H                 -5.24094200   -1.56246800    3.89534100 C                 -0.28038200   -1.97312300    3.39711600 H                  0.73232700   -2.19579800    3.74917000 H                 -0.29844600   -2.13296700    2.31217300 C                 -0.52005200   -0.28867000    5.25564300 H                 -0.80223900    0.73383800    5.52434200 H                 -1.13281400   -0.97047900    5.85654400 H                  0.52441200   -0.43740200    5.54999100 C                 -4.52408500    0.75386200   -0.47663400 H                 -5.34765900    0.07455000   -0.22343400 H                 -4.83658000    1.33649200   -1.35008800 H                 -3.66825300    0.13761600   -0.77028300 C                 -5.36222100    2.59236400    0.99761700 H                 -6.27799400    2.02949700    1.21070400 H                 -5.17790600    3.25044900    1.85207900 H                 -5.56755900    3.22744600    0.12951700 C                 -2.56061000    3.01126100   -2.47301500 H                 -2.26376400    3.71721600   -1.69100300 H                 -2.45869100    3.51865700   -3.43960200 H                 -3.62257400    2.78571200   -2.32737100 C                 -2.27785000    0.69370900   -3.39986300 H                 -3.28102200    0.39037600   -3.08363700 H                 -2.36684500    1.08117100   -4.42170000 H                 -1.65294400   -0.20527400   -3.43143500 C                  3.55758300    4.66556300   -1.16124100 H                  4.25263800    4.51036800   -1.99396200 H                  2.87066000    5.46465300   -1.45954100 H                  4.14753500    5.02515500   -0.31217700 C                  3.82284100    2.27616000   -0.44928800 H                  4.48187600    2.05404300   -1.29785100 H                  4.45054600    2.57271600    0.39791600 H                  3.31882700    1.34358700   -0.17324700 H                 -0.95301700   -2.71221600    3.84732800 H                  3.69738200   -3.22846500    2.70025100 Al                 0.89718900   -1.12747900   -0.37746400 Al                 0.19511300    1.09282500    0.87665900 H                  1.44338600   -0.76685300   -1.84218900 H                  1.33575800    1.66720800    1.86524300 C                  4.19161900   -2.32256400   -1.57334000 H                  3.95283900   -3.35266400   -1.28150100 H                  3.31189100   -1.94484300   -2.10733300 H                  5.03112400   -2.35477600   -2.27203600 C                  7.31347100    0.80996000    0.82470000 H                  8.14457300    0.25985200    0.37321900 H                  7.21063200    1.74538700    0.26068200 H                  7.60094700    1.08000800    1.84444900 C                  2.98072700   -0.52233200    2.98836800 H                  3.02552600    0.45806800    3.46962000 H                  1.94753300   -0.69194000    2.68200600 H                  3.22514400   -1.27476000    3.75125300 C                  0.42722000   -3.34538200   -3.34748000 H                  1.08214300   -2.47033900   -3.25915200 H                  0.88805500   -4.13142700   -2.73746000 H                  0.43235600   -3.67490400   -4.38930900 C                 -4.43682200   -2.57907300   -4.39110100 H                 -4.46985700   -1.58257800   -4.84892500 H                 -4.31350600   -3.29733500   -5.20688000 H                 -5.41408700   -2.75246700   -3.93217900 C                 -2.78515800   -2.22768600    0.35853400 H                 -2.15587600   -1.41911200    0.75007300 H                 -3.82757000   -1.96069200    0.54880300 H                 -2.54843100   -3.10742500    0.97064100mesBDIAl(I)N                  1.39818300   -0.46943700   -0.03461400 C                  1.25207700   -1.79716400   -0.11370800 C                 -0.00000300   -2.42121400   -0.15152600 H                 -0.00000700   -3.50352900   -0.21664400 C                 -1.25207900   -1.79715500   -0.11371400 N                 -1.39817600   -0.46942700   -0.03462100 C                  2.47097200   -2.67430100   -0.16528800 H                  3.10320600   -2.41755800   -1.02163400 H                  2.20149000   -3.72928000   -0.23344700 H                  3.09701100   -2.52928200    0.72134300 C                 -2.47098000   -2.67428400   -0.16529000 H                 -3.09700700   -2.52927300    0.72135100 H                 -2.20150600   -3.72926400   -0.23346600 H                 -3.10322400   -2.41752700   -1.02162500 C                  2.73051500    0.05788500    0.00121500 C                  3.36501600    0.41655000   -1.19793700 C                  4.64948500    0.95752700   -1.13720600 H                  5.14679100    1.23239800   -2.06682400 C                  5.30824700    1.16030100    0.07621900 C                  4.64514000    0.80517900    1.25171500 H                  5.13895900    0.95973200    2.21054600 C                  3.36061000    0.26132200    1.23860400 C                 -2.73050600    0.05790300    0.00120500 C                 -3.36060300    0.26134200    1.23859200 C                 -4.64513300    0.80520100    1.25169900 H                 -5.13895300    0.95975600    2.21053000 C                 -5.30823600    1.16032300    0.07620200 C                 -4.64947100    0.95754700   -1.13722200 H                 -5.14677400    1.23241800   -2.06684000 C                 -3.36500300    0.41656900   -1.19794900 Al                 0.00000900    0.99234000    0.04883400 C                  2.66503400    0.22427300   -2.50756100 H                  2.38786400   -0.82245300   -2.68091300 H                  1.72684600    0.79412800   -2.54112500 H                  3.28762500    0.55151900   -3.34343000 C                  6.67422300    1.77546200    0.11803900 H                  7.24911300    1.54731300   -0.78387300 H                  6.61812300    2.86781400    0.19188100 H                  7.24884400    1.42808000    0.98128600 C                  2.65575200   -0.09428100    2.51095300 H                  1.72027500    0.47125800    2.61593100 H                  2.37313100   -1.15314300    2.54791600 H                  3.27746000    0.11982500    3.38333600 C                 -2.65574800   -0.09426000    2.51094400 H                 -2.37312000   -1.15312000    2.54790500 H                 -1.72027600    0.47128600    2.61592700 H                 -3.27746200    0.11984000    3.38332500 C                 -2.66502000    0.22428500   -2.50757100 H                 -1.72679600    0.79408100   -2.54111100 H                 -2.38791300   -0.82245500   -2.68094800 H                 -3.28758300    0.55158900   -3.34343700 C                 -6.67421100    1.77548500    0.11801800 H                 -6.61811000    2.86783700    0.19185200 H                 -7.24910100    1.54733000   -0.78389300 H                 -7.24883300    1.42810900    0.98126700mesBDIAl(I)-d N                 -1.46439100   -2.50732000    0.30878100 C                 -1.31637700   -3.54453500    1.14319800 C                 -0.08413700   -3.93803000    1.67114600 H                 -0.10059300   -4.79621400    2.33258100 C                  1.18100400   -3.43686600    1.34405300 N                  1.37836900   -2.37482300    0.55129700 C                 -2.50454900   -4.39656500    1.49768100 H                 -3.30679100   -3.81090000    1.95547800 H                 -2.22091500   -5.19575800    2.18359400 H                 -2.93912200   -4.84749100    0.59910100 C                  2.35856800   -4.20459000    1.88269700 H                  2.97596000   -4.59108200    1.06476200 H                  2.02743500   -5.04485700    2.49404500 C                 -2.77714800   -2.33021000   -0.24000500 C                 -3.75224900   -1.63703000    0.49181000 C                 -5.04806500   -1.57979300   -0.02297800 H                 -5.81493700   -1.06747800    0.55703200 C                 -5.38189400   -2.13435700   -1.25834800 C                 -4.37921500   -2.78640900   -1.97873000 H                 -4.61670300   -3.22982100   -2.94538100 C                 -3.08017900   -2.90843200   -1.48458100 C                  2.75399000   -2.08834000    0.25250100 C                  3.32142100   -2.62546600   -0.91472200 C                  4.69490200   -2.48180000   -1.11559800 H                  5.14106800   -2.91188800   -2.01228800 C                  5.51190900   -1.83925400   -0.18498100 C                  4.91074200   -1.28570600    0.94546600 H                  5.52591600   -0.75350200    1.67129500 C                  3.53760400   -1.37483000    1.17436800 N                  1.37836200    2.37482500   -0.55129300 C                  1.18099500    3.43687000   -1.34404700 C                 -0.08414600    3.93803300   -1.67113900 H                 -0.10060200    4.79621800   -2.33257300 C                 -1.31638600    3.54453500   -1.14319400 N                 -1.46439900    2.50731900   -0.30877800 C                  2.35855900    4.20459600   -1.88269000 H                  2.97595400    4.59108100   -1.06475400 H                  2.02742600    5.04486700   -2.49403100 H                  3.02095100    3.57578500   -2.48403700 C                 -2.50455900    4.39656300   -1.49767800 H                 -3.30679700    3.81089600   -1.95548100 H                 -2.22092500    5.19575900   -2.18358700 H                 -2.93913800    4.84748400   -0.59909800 C                  2.75398300    2.08834300   -0.25250000 C                  3.32141500    2.62546500    0.91472500 C                  4.69489600    2.48180200    1.11559700 H                  5.14106300    2.91188800    2.01228800 C                  5.51190400    1.83926300    0.18497600 C                  4.91073600    1.28571600   -0.94547100 H                  5.52591000    0.75351600   -1.67130300 C                  3.53759700    1.37483800   -1.17437100 C                 -2.77715600    2.33020600    0.24000600 C                 -3.75225600    1.63702400   -0.49181200 C                 -5.04807200    1.57978400    0.02297400 H                 -5.81494200    1.06746800   -0.55703800 C                 -5.38190500    2.13434800    1.25834300 C                 -4.37922800    2.78640200    1.97872700 H                 -4.61671900    3.22981300    2.94537800 C                 -3.08019100    2.90842600    1.48458100 H                  3.02096200   -3.57577600    2.48403800 Al                -0.02874400   -1.29405500   -0.39556000 Al                -0.02875100    1.29405500    0.39556100 C                 -2.03084300    3.65968900    2.24405100 H                 -1.18430000    3.00638000    2.49931300 H                 -1.60838300    4.48277500    1.65451800 H                 -2.43175000    4.07561500    3.17156900 C                 -6.76574000    1.98942600    1.81532000 H                 -6.84329300    1.10987200    2.46737400 H                 -7.05711500    2.85434500    2.41828300 H                 -7.50925400    1.86338800    1.02292900 C                 -3.40886100    0.98396200   -1.79498300 H                 -2.56914000    0.28560200   -1.67952300 H                 -4.25857600    0.41991300   -2.18855700 H                 -3.09499500    1.70648900   -2.55805500 C                  2.91995600    0.76540100   -2.39536600 H                  3.64261200    0.14243200   -2.92924500 H                  2.05512600    0.14071100   -2.13502700 H                  2.54424300    1.52126500   -3.09660900 C                  6.98561300    1.71189300    0.42158600 H                  7.39033700    2.58562300    0.94090900 H                  7.21871700    0.83727600    1.04310000 H                  7.53802800    1.58912900   -0.51486000 C                  2.47562300    3.37574300    1.89692900 H                  1.95747500    4.22328200    1.43110800 H                  1.68650300    2.73272000    2.31247900 H                  3.07521300    3.75760900    2.72699100 C                  6.98561800   -1.71188000   -0.42159400 H                  7.21871800   -0.83726100   -1.04310500 H                  7.53803400   -1.58911900    0.51485200 H                  7.39034200   -2.58560800   -0.94092100 C                  2.47562900   -3.37575000   -1.89692200 H                  1.95748400   -4.22328800   -1.43109600 H                  1.68650800   -2.73273000   -2.31247400 H                  3.07521900   -3.75761800   -2.72698300 C                  2.91996300   -0.76539100    2.39536200 H                  3.64261800   -0.14241900    2.92923800 H                  2.05513200   -0.14070300    2.13502200 H                  2.54425000   -1.52125400    3.09660700 C                 -3.40885900   -0.98396700    1.79498200 H                 -2.56913600   -0.28560900    1.67952500 H                 -4.25857500   -0.41991600    2.18855200 H                 -3.09499800   -1.70649300    2.55805600 C                 -2.03082900   -3.65969300   -2.24404800 H                 -1.18428600   -3.00638300   -2.49931000 H                 -1.60836800   -4.48277800   -1.65451400 H                 -2.43173300   -4.07562100   -3.17156700 C                 -6.76572800   -1.98943600   -1.81532800 H                 -6.84327200   -1.10990200   -2.46741000 H                 -7.05711400   -2.85437100   -2.41826400 H                 -7.50924000   -1.86336400   -1.022940007 N                 -1.47282600   -2.51305600    0.28141600 C                 -1.32865100   -3.52593200    1.14384500 C                 -0.10895200   -3.88626200    1.72497500 H                 -0.13947700   -4.71787200    2.41861100 C                  1.16268800   -3.38752200    1.41893200 N                  1.38446200   -2.36335400    0.58624700 C                 -2.51424600   -4.38694600    1.48521900 H                 -3.33771000   -3.80456200    1.90819300 H                 -2.23692500   -5.16597800    2.19601500 H                 -2.91736600   -4.86526600    0.58619800 C                  2.32679400   -4.11843300    2.03179400 H                  2.94724000   -4.58040000    1.25623600 H                  1.98072100   -4.90095300    2.70776900 C                 -2.78183500   -2.34696300   -0.27675700 C                 -3.75286800   -1.63846100    0.44772600 C                 -5.05050600   -1.58508400   -0.06118000 H                 -5.81406000   -1.06324000    0.51445300 C                 -5.39009900   -2.16168900   -1.28536100 C                 -4.39489800   -2.83885300   -1.99228100 H                 -4.64149100   -3.30904100   -2.94386900 C                 -3.09380500   -2.96123800   -1.50132700 C                  2.76499400   -2.09943700    0.29624100 C                  3.36229600   -2.74363100   -0.80030500 C                  4.73941700   -2.60830600   -0.97994700 H                  5.21001600   -3.11901400   -1.82003400 C                  5.53114400   -1.87706600   -0.09347800 C                  4.90241800   -1.23126100    0.97090700 H                  5.49840700   -0.63567400    1.66254500 C                  3.52406400   -1.30663900    1.17229100 N                  1.38892500    2.37105000   -0.56766800 C                  1.16888000    3.40404200   -1.39039400 C                 -0.10148700    3.90724800   -1.69411100 H                 -0.12934800    4.74537900   -2.37994000 C                 -1.32428500    3.53906500   -1.12464300 N                 -1.47170900    2.51947300   -0.27088300 C                  2.33432500    4.13727100   -1.99816800 H                  2.96558200    4.57833100   -1.21927000 H                  1.98956400    4.93526300   -2.65644500 H                  2.98695700    3.46976100   -2.56777100 C                 -2.51053600    4.39732600   -1.47064900 H                 -3.32654600    3.81363700   -1.90625200 H                 -2.23016000    5.18290500   -2.17299400 H                 -2.92539200    4.86687700   -0.57238700 C                  2.77045600    2.09692700   -0.29049700 C                  3.37909000    2.72352400    0.80964700 C                  4.75768200    2.58270300    0.97497400 H                  5.23678500    3.08029400    1.81815600 C                  5.54007300    1.86463800    0.06986300 C                  4.90027500    1.23573800   -0.99820600 H                  5.48929200    0.65136700   -1.70518600 C                  3.52028100    1.31548700   -1.18506500 C                 -2.78447300    2.34669600    0.27650600 C                 -3.74763300    1.63733900   -0.45746000 C                 -5.04954200    1.58041500    0.04015700 H                 -5.80699600    1.05807100   -0.54302400 C                 -5.40087600    2.15400500    1.26240400 C                 -4.41303400    2.83136300    1.97945100 H                 -4.66864000    3.29884200    2.92999800 C                 -3.10816200    2.95759500    1.49975200 H                  2.99008000   -3.44611200    2.58286200 Al                -0.02526300   -1.29000600   -0.31913000 Al                -0.02457800    1.29768000    0.33368400 H                  0.13620500   -1.56853900   -1.89694700 H                  0.12914400    1.57610300    1.91234700 C                  2.86235300    0.63397500   -2.34552000 H                  3.57583100    0.00867000   -2.88846200 H                  2.03282800   -0.00808700   -2.02660700 H                  2.42880800    1.34822400   -3.05665900 C                  2.57639000    3.57564600    1.74404500 H                  2.05136800    4.37840600    1.21134800 H                  1.79714400    2.99288200    2.24862300 H                  3.21379900    4.03512500    2.50357400 C                  7.02004800    1.74342200    0.26733400 H                  7.44455100    2.64515900    0.71836600 H                  7.26948300    0.90761200    0.93411300 H                  7.54183700    1.55956800   -0.67666600 C                 -2.08956400    3.77253800    2.23554200 H                 -1.20654100    3.17654900    2.49214500 H                 -1.72344300    4.60573500    1.62234800 H                 -2.50814300    4.19107000    3.15406100 C                 -6.78990500    2.00747500    1.80558400 H                 -6.88328800    1.10409700    2.42207000 H                 -7.07230800    2.85268400    2.43958600 H                 -7.53074900    1.92198800    1.00521200 C                 -3.38667000    0.97959200   -1.75398800 H                 -2.52570400    0.30995200   -1.63415700 H                 -4.21828300    0.38365400   -2.13885700 H                 -3.09882700    1.70398800   -2.52565400 C                  7.00913100   -1.75945600   -0.30748300 H                  7.25307900   -0.92569900   -0.97882400 H                  7.54167900   -1.57441200    0.63029800 H                  7.42669400   -2.66323500   -0.76084600 C                  2.54881900   -3.60679600   -1.71514600 H                  2.02663700   -4.40079800   -1.16665600 H                  1.76670800   -3.02873100   -2.22079300 H                  3.17800500   -4.07858900   -2.47395700 C                  2.87939900   -0.61276400    2.33317900 H                  3.56982400    0.09604900    2.79814300 H                  1.98159400   -0.05991100    2.03450400 H                  2.55050900   -1.31816200    3.10729200 C                 -3.40424400   -0.97731300    1.74593100 H                 -2.54738200   -0.30168100    1.63057800 H                 -4.24214400   -0.38682900    2.12541100 H                 -3.11587600   -1.69891700    2.51992900 C                 -2.06667600   -3.77339800   -2.22828900 H                 -1.19332700   -3.16871500   -2.49742100 H                 -1.68559400   -4.59140000   -1.60399600 H                 -2.48254300   -4.21130000   -3.13897300 C                 -6.77461900   -2.01868100   -1.84085700 H                 -6.86594600   -1.11315100   -2.45449500 H                 -7.04762200   -2.86249200   -2.48078300 H                 -7.52329800   -1.93939600   -1.047152009N                 -1.43676700    0.33298000    0.07776500 C                 -1.26237800    1.65761800    0.07162800 C                 -0.00000200    2.26508000   -0.00000200 H                 -0.00000500    3.34922900   -0.00000100 C                  1.26237400    1.65762500   -0.07163200 N                  1.43676700    0.33298400   -0.07776800 C                 -2.44990900    2.57391400    0.16599300 H                 -2.94418900    2.69582600   -0.80248100 H                 -2.14267700    3.56416600    0.50512500 H                 -3.20309200    2.17878100    0.85243100 C                  2.44990500    2.57392200   -0.16598700 H                  2.94417600    2.69583100    0.80249200 H                  2.14267700    3.56417600   -0.50511800 H                  3.20309400    2.17879200   -0.85242100 C                 -2.74281400   -0.23138500    0.06086000 C                 -3.61153800   -0.02834000   -1.01749000 C                 -4.85861400   -0.64202400   -1.03672900 H                 -5.52369200   -0.47811000   -1.88013200 C                 -5.24915100   -1.47547200    0.00942800 C                 -4.37894600   -1.69727100    1.07318300 H                 -4.67141900   -2.35091800    1.89011600 C                 -3.13004900   -1.08335800    1.10090300 C                  2.74281300   -0.23138200   -0.06086400 C                  3.61153800   -0.02834200    1.01748600 C                  4.85861400   -0.64202800    1.03672500 H                  5.52369100   -0.47811400    1.88012900 C                  5.24915000   -1.47547400   -0.00943300 C                  4.37894500   -1.69726900   -1.07318900 H                  4.67141700   -2.35091400   -1.89012400 C                  3.13005000   -1.08335400   -1.10090800 H                 -6.22227600   -1.95706700   -0.01037200 H                  6.22227500   -1.95707000    0.01036700 Al                 0.00000600   -0.99095500    0.00001100 H                  0.09620300   -1.79999600    1.36084900 H                 -0.09620200   -1.80003800   -1.36080000 H                  3.28945300    0.59561400    1.84691900 H                  2.44441200   -1.24994200   -1.92761900 H                 -3.28945000    0.59561700   -1.84692000 H                 -2.44441000   -1.24994800    1.9276130010 N                 -1.51559100    2.42455300    1.06002600 C                 -1.80821800    3.62201200    0.54016100 C                 -0.86919800    4.42161000   -0.12370900 H                 -1.23968900    5.37810200   -0.47419600 C                  0.50730900    4.21183700   -0.29949300 N                  1.13160500    3.09225900    0.07947200 C                 -3.18364000    4.21097300    0.69450700 H                 -3.89621800    3.74700500    0.00512800 H                 -3.16604000    5.28097500    0.48421900 H                 -3.57969200    4.05443300    1.70104600 C                  1.26028700    5.37370800   -0.89251000 H                  2.26815800    5.46272000   -0.48291600 H                  0.71804200    6.30155000   -0.70360900 C                 -2.49989900    1.73881800    1.82782100 C                 -3.70621400    1.31121500    1.26414100 C                 -4.67509800    0.71028600    2.06034000 H                 -5.61151700    0.39274300    1.60778500 C                 -4.43811400    0.48968900    3.41504300 C                 -3.21069700    0.85285100    3.96451200 H                 -3.00467800    0.66425600    5.01488200 C                 -2.24418300    1.47479900    3.17880500 C                  2.53199300    2.94780200   -0.10627100 C                  3.31947300    2.54365900    0.97958200 C                  4.70019400    2.43505300    0.84208000 H                  5.29701000    2.13279100    1.69859800 C                  5.31355100    2.71841400   -0.37459900 C                  4.52817700    3.05887300   -1.47426300 H                  4.99046400    3.23059500   -2.44272300 C                  3.14751800    3.15305200   -1.34912300 N                  1.45124100   -2.02485300   -0.31864400 C                  1.36822300   -3.16727900   -0.99014200 C                  0.39434100   -3.38767100   -1.98705700 H                  0.48020000   -4.31940300   -2.53620200 C                 -0.77189200   -2.64940100   -2.19775300 N                 -1.02359900   -1.47409900   -1.59176100 C                  2.30355300   -4.30879400   -0.70478800 H                  3.04119000   -4.39938100   -1.51000600 H                  1.75600300   -5.25387800   -0.66676900 H                  2.84820400   -4.17373800    0.23169300 C                 -1.80180200   -3.22660200   -3.12862300 H                 -2.73481200   -3.46544300   -2.60976700 H                 -1.43367600   -4.13272900   -3.61171600 H                 -2.06632600   -2.49534300   -3.90048300 C                  2.45443500   -1.79306900    0.67242600 C                  3.79053400   -1.57103700    0.28423500 C                  4.70829100   -1.19256300    1.26871600 H                  5.74002700   -0.99996700    0.97830300 C                  4.32365300   -1.03877600    2.59243600 C                  3.00528600   -1.28515300    2.96272400 H                  2.71167000   -1.17274600    4.00234600 C                  2.05114800   -1.66977600    2.02098500 C                  4.26226300   -1.70412500   -1.15125900 H                  3.43107700   -2.07396200   -1.76109200 C                  0.63314600   -2.02075700    2.42715800 H                 -0.04159900   -1.58352800    1.67291800 C                 -2.38983500   -1.06887400   -1.51655200 C                 -3.22617700   -1.70743400   -0.57665900 C                 -4.58565600   -1.38953900   -0.57394700 H                 -5.24360900   -1.89099000    0.13470800 C                 -5.10427100   -0.43532700   -1.44132300 C                 -4.25419100    0.23332100   -2.31555600 H                 -4.65684900    0.99452400   -2.98083300 C                 -2.89152600   -0.07315700   -2.37688800 C                 -2.68292600   -2.69745600    0.43912600 H                 -1.59598400   -2.75761300    0.30551600 C                 -1.97757900    0.67034800   -3.32986800 H                 -1.04989000    0.09061400   -3.42184900 H                  6.39259200    2.64273400   -0.47609100 H                  5.05024700   -0.73142800    3.34027500 H                 -6.16588500   -0.20127700   -1.42568900 H                 -5.19535700    0.01522600    4.03289200 C                 -1.60922300    2.03466100   -2.74375500 H                 -0.88221200    2.55861800   -3.37463200 H                 -1.17188300    1.93981700   -1.74345000 C                 -2.57037900    0.82651400   -4.72688200 H                 -2.86975400   -0.13598200   -5.15338600 H                 -3.45401900    1.47448500   -4.72464600 H                 -1.84100700    1.28247000   -5.40321800 C                 -2.94118200   -2.19874400    1.85872000 H                 -4.01334700   -2.10886300    2.06620600 H                 -2.52228800   -2.89150500    2.59720400 H                 -2.49659800   -1.21135400    2.02573600 C                 -3.25543100   -4.10128000    0.25388600 H                 -4.34588200   -4.10445200    0.36813500 H                 -3.02452500   -4.51575000   -0.73231400 H                 -2.84460100   -4.78619700    1.00350000 C                  0.43676700   -3.53979100    2.39480400 H                  0.58789100   -3.95538800    1.39416900 H                  1.14156400   -4.03348500    3.07401400 H                 -0.57566800   -3.80982600    2.71458800 C                  0.22305800   -1.47021200    3.78632200 H                 -0.84449300   -1.64594900    3.95496100 H                  0.76454500   -1.96340800    4.60240100 H                  0.40272800   -0.39290200    3.85355400 C                  5.41937900   -2.69616400   -1.26879300 H                  6.31146300   -2.32618600   -0.75105000 H                  5.17460900   -3.67142300   -0.83608200 H                  5.69447600   -2.84866500   -2.31759800 C                  4.66113800   -0.34915900   -1.72543500 H                  5.50845600    0.07596800   -1.17588300 H                  4.95609300   -0.44264400   -2.77630000 H                  3.83703600    0.36739200   -1.66745500 H                  2.53106700    3.37757100   -2.21483400 H                  2.83980200    2.34283600    1.93454900 H                 -2.49729000    2.67305800   -2.64764900 H                  1.36546500    5.27619300   -1.97747200 H                 -3.87506200    1.44950100    0.19847200 H                 -1.29082300    1.78098300    3.60120100 Al                 0.22028900    1.50154700    0.86279900 Al                 0.48422700   -0.40463300   -0.93316700 H                  0.76074800    1.30533300    2.35673000 H                  1.28782500    0.03386200   -2.26437200phBDIAl N                  1.41864000    0.27297800    0.11151900 C                  1.25808700    1.59615200    0.23917400 C                 -0.00000100    2.20401000    0.31872000 H                  0.00000000    3.27904500    0.46036300 C                 -1.25808800    1.59615200    0.23916900 N                 -1.41863800    0.27297600    0.11152500 C                  2.45644200    2.49961500    0.34309700 H                  3.20659400    2.08178700    1.02050000 H                  2.16772700    3.48970700    0.69938300 H                  2.95316100    2.62351700   -0.62367400 C                 -2.45644300    2.49961700    0.34307700 H                 -2.95315200    2.62351700   -0.62369900 H                 -2.16773000    3.48970800    0.69936400 H                 -3.20660200    2.08179100    1.02047300 C                  2.73906200   -0.24632500   -0.01836800 C                  3.25278000   -1.07776700    0.98168200 C                  4.51492500   -1.64802100    0.84128200 H                  4.90305700   -2.28912800    1.62771900 C                  5.27460400   -1.40208700   -0.29868700 C                  4.75995700   -0.58748800   -1.30533300 H                  5.33878300   -0.40236400   -2.20609700 C                  3.49903100   -0.01723000   -1.17047900 C                 -2.73906100   -0.24632700   -0.01836500 C                 -3.49902800   -0.01723400   -1.17047700 C                 -4.75995500   -0.58748900   -1.30533200 H                 -5.33878000   -0.40236400   -2.20609800 C                 -5.27460600   -1.40208400   -0.29868500 C                 -4.51492700   -1.64801900    0.84128400 H                 -4.90306100   -2.28912500    1.62772200 C                 -3.25278000   -1.07776900    0.98168500 H                  6.25797500   -1.84979200   -0.40768300 H                 -6.25797800   -1.84978700   -0.40767900 Al                 0.00000000   -1.20786400    0.05281400 H                 -2.65472100   -1.26345800    1.87007300 H                 -3.08201700    0.59735600   -1.96464100 H                  2.65472100   -1.26345500    1.87007100 H                  3.08202200    0.59736300   -1.96464100phBDIAl_d N                  1.44499100    2.20037800   -0.38707600 C                  1.67486400    2.85737200    0.75486400 C                  0.64991600    3.19040100    1.64973600 H                  0.95168700    3.75306600    2.52615900 C                 -0.73362900    3.03265200    1.47232200 N                 -1.26281800    2.36909200    0.44346600 C                  3.05963600    3.32954400    1.10247700 H                  3.69070000    2.49681000    1.43166000 H                  3.02831100    4.06658000    1.90645500 H                  3.55886000    3.77060300    0.23534300 C                 -1.62517300    3.69499200    2.48694200 H                 -2.45547800    4.21651900    2.00269300 H                 -1.06353100    4.40815700    3.09211600 C                  2.49852200    1.97175100   -1.31277500 C                  3.59731500    1.17125100   -0.99203100 C                  4.57524200    0.90673700   -1.94525600 H                  5.42093000    0.27745300   -1.67707600 C                  4.46697200    1.43083400   -3.23106900 C                  3.36347700    2.21483900   -3.56067900 H                  3.26341000    2.62156700   -4.56331400 C                  2.38077200    2.47941000   -2.61195900 C                 -2.67485600    2.24620400    0.34059500 C                 -3.34188600    2.84448100   -0.73328600 C                 -4.72405400    2.72625300   -0.85248000 H                 -5.23430900    3.20927500   -1.68163300 C                 -5.44983800    2.00087400    0.08845800 C                 -4.78387300    1.37398400    1.13929000 H                 -5.34034900    0.78131700    1.86073000 C                 -3.40284500    1.48451100    1.26207500 N                 -1.32083800   -2.51883700   -0.17072300 C                 -0.99756800   -3.78411400   -0.44936000 C                  0.26479800   -4.32589300   -0.17659300 H                  0.37410000   -5.38631700   -0.37588600 C                  1.39744700   -3.68749500    0.34035000 N                  1.44046400   -2.37179300    0.60791300 C                 -2.01880900   -4.69766400   -1.06489000 H                 -2.83532500   -4.91129700   -0.36859700 H                 -1.56821600   -5.64349800   -1.36782900 H                 -2.48180500   -4.22812700   -1.93789500 C                  2.56641500   -4.59368900    0.63209000 H                  3.24891400   -4.67510300   -0.21896700 H                  2.20362400   -5.60073600    0.84746800 H                  3.15605800   -4.24167900    1.48056500 C                 -2.67156100   -2.10504100   -0.39977300 C                 -3.67607600   -2.45502800    0.50690100 C                 -4.99200300   -2.06827600    0.27258700 H                 -5.76991100   -2.35078500    0.97726200 C                 -5.30893400   -1.31527900   -0.85557100 C                 -4.30372800   -0.94068500   -1.74242500 H                 -4.54108600   -0.33503500   -2.61288100 C                 -2.98611100   -1.33218300   -1.51942600 C                  2.66444700   -1.78049900    1.01588900 C                  3.85535400   -1.96374500    0.29821800 C                  5.04207400   -1.38208300    0.73106100 H                  5.95644000   -1.55372900    0.16891800 C                  5.05703700   -0.56156700    1.85737900 C                  3.86849500   -0.33378700    2.54880200 H                  3.86130500    0.30665500    3.42694200 C                  2.68450800   -0.94137700    2.13969000 H                 -6.52889700    1.91118500   -0.00408300 H                 -6.33508800   -1.00509800   -1.03301200 H                  5.98258700   -0.09938900    2.18796200 H                  5.23125300    1.22231700   -3.97435200 H                 -2.87040800    0.97820100    2.06433500 H                 -2.76556400    3.42083500   -1.45222900 H                 -2.07503400    2.96145000    3.16292100 H                  3.66513800    0.74219500    0.00278800 H                  1.51519100    3.08810800   -2.86024000 Al                -0.24429700    1.21087200   -0.84804000 Al                -0.15665400   -1.10748300    0.60822200 H                 -2.19203100   -1.04410900   -2.20325700 H                 -3.41323000   -3.04122200    1.38467500 H                  1.76323700   -0.78465200    2.69526000 H                  3.84021700   -2.55356500   -0.61334200phAlHAlHph N                 -1.13024400   -1.99062900   -1.05212600 C                 -1.53337700   -3.01818100   -0.30247500 C                 -0.71544400   -3.62283200    0.66801100 H                 -1.15582100   -4.45702900    1.20291300 C                  0.64038500   -3.37237500    0.93427800 N                  1.33081300   -2.40188100    0.33618600 C                 -2.89733600   -3.61695900   -0.50235700 H                 -3.66553900   -3.02409800    0.00645900 H                 -2.93550700   -4.63001700   -0.09873400 H                 -3.17102300   -3.64331100   -1.56010200 C                  1.33808800   -4.27461900    1.91076900 H                  2.21509700   -4.73997300    1.44976600 H                  0.67387400   -5.06026500    2.27209900 C                 -1.99515400   -1.38554700   -2.00381200 C                 -3.19606800   -0.78413500   -1.61345700 C                 -3.98730000   -0.12679600   -2.54884200 H                 -4.91585100    0.33901300   -2.22675500 C                 -3.58782600   -0.05371800   -3.88141600 C                 -2.38310900   -0.63615800   -4.26970400 H                 -2.05912000   -0.57831000   -5.30526500 C                 -1.58443400   -1.29269900   -3.33841800 C                  2.71321000   -2.22066800    0.62498600 C                  3.67622700   -2.68082000   -0.27771400 C                  5.02779400   -2.46117200   -0.02962700 H                  5.76960200   -2.82928300   -0.73327300 C                  5.42949700   -1.76831200    1.11099500 C                  4.46983200   -1.28925300    2.00059600 H                  4.77335100   -0.73548600    2.88513600 C                  3.11590700   -1.51157600    1.76162700 N                  1.03842300    2.71710700    0.23827500 C                  0.56989600    3.92888800    0.52850600 C                 -0.62540100    4.12318500    1.24482200 H                 -0.83690900    5.15080800    1.52142200 C                 -1.62574200    3.19993700    1.57524100 N                 -1.51960300    1.88162200    1.33772900 C                  1.32344200    5.16848100    0.13531100 H                  2.39984500    4.99263300    0.08354700 H                  1.12478300    5.96865100    0.85115400 H                  1.00903800    5.53404100   -0.84742600 C                 -2.82843300    3.78315700    2.27074800 H                 -3.57532900    4.15569300    1.56286500 H                 -2.51473300    4.63653600    2.87546400 H                 -3.32518000    3.05732500    2.91682200 C                  2.18738500    2.51356300   -0.56406000 C                  3.16293700    1.62228700   -0.09746500 C                  4.25576700    1.29389100   -0.89194300 H                  4.99620800    0.59421600   -0.51255000 C                  4.39024300    1.85006500   -2.16185100 C                  3.42054900    2.73257600   -2.63233700 H                  3.50836200    3.15694500   -3.62884900 C                  2.32150800    3.05945600   -1.84499000 C                 -2.59638900    0.99344800    1.51968100 C                 -3.91442200    1.27886700    1.12687200 C                 -4.92749600    0.34278800    1.30178200 H                 -5.93919000    0.58952000    0.99009000 C                 -4.64895600   -0.91289000    1.83990500 C                 -3.33674800   -1.22291800    2.19432900 H                 -3.09330400   -2.20286300    2.59758000 C                 -2.32313300   -0.28423100    2.03811900 H                  6.48508200   -1.59594600    1.30179200 H                  5.23930100    1.58881300   -2.78655700 H                 -5.44133300   -1.64459200    1.96873100 H                 -4.20567800    0.46193900   -4.61107500 H                  2.35772000   -1.11905300    2.43640900 H                  3.34758600   -3.20510000   -1.17090700 H                  1.71207800   -3.71003300    2.77054100 H                 -3.48974800   -0.82391200   -0.56923400 H                 -0.63667400   -1.73772000   -3.62812200 Al                 0.51324000   -0.94901400   -0.71688900 Al                 0.25009500    1.05261300    0.94969800 H                  1.31321700   -0.83475200   -2.09399500 H                  0.90295000    0.82261600    2.41131200 H                  1.54225300    3.70979200   -2.23231800 H                  3.05966000    1.19684200    0.89999300 H                 -1.30820000   -0.52979800    2.34884700 H                 -4.13536600    2.22745000    0.64860400
