# Supplementary material for: Exploring Equilibria between Aluminium(I) and Aluminium(III): The Formation of Dihydroalanes, Masked Dialumenes and Aluminium(I) Species
Source: Angew Chem Int Ed Engl. 2022 May 17;61(31):e202205901. doi: 10.1002/anie.202205901 (PMC9401008; doi:10.1002/anie.202205901)
Supplement: Supplementary file 2 — Supporting Information [file ANIE-61-0-s002.pdf]

Angewandte Chemie
Eine Zeitschrift der Gesellschaft Deutscher Chemiker

Supporting Information

Exploring Equilibria between Aluminium(I) and Aluminium(III): The Formation of Dihydroalanes, Masked Dialumenes and Aluminium(I) Species

C. Bakewell, K. Hobson, C. J. Carmalt*

Table of contents

1. **General Experimental Section** **S3**
2. **Synthetic Procedures** **S4**
3. **Supporting Figures** **S8**
4. **X-ray Crystallographic Data** **S17**
5. **Computational Details** **S21**
6. **Multinuclear NMR Data** **S26**
7. **References** **S32**
8. **XYZ Coordinates** **S33**

1. General Experimental Section

All manipulations were carried out using standard Schlenk-line and glovebox techniques under an inert atmosphere of argon or dinitrogen. A MBraun Labmaster glovebox was employed, operating at < 0.1 ppm O_2 and < 0.1 ppm H_2O. Solvents were dried over activated alumina from an SPS (solvent purification system) based upon the Grubbs design and degassed before use. Glassware was dried for 12 h at 120 °C prior to use. Benzene-d_6 was stored over activated 3Å molecular sieves. NMR-scale reactions were conducted in J. Young's tap tubes and prepared in a glovebox. All heating mentioned was done using a DrySyn NMR tube heating block. ^{1}H (tetramethylsilane; 0 ppm) and ^{13}C (tetramethylsilane; 0 ppm) spectra were obtained on BRUKER 400 MHz or 500 MHz machines unless otherwise stated; all peak intensities are derived from internal standard peaks with values quoted in ppm. Data was processed using the MestReNova or Topsin software. Variable temperature measurements were ran by Dr Abil Aliev. C^{IV} refers to quaternary carbons. Mass spectrometry was ran on a Waters G2-XS Xevo QToF instrument in positive mode by Oscar Ayrton. Elemental analysis was conducted by Elemental Microanalysis Ltd. and have been obtained to the best of our abilities given the extremely air and moisture sensitive nature of the compounds.

Trimethylamine alane and compound **A** were synthesised according to literature procedures.[1,2] The synthesis of **1** was previously reported in our research group.[3] Diphenyl acetylene was purchased from Sigma Aldrich and dried under high vacuum prior to use. Hexafluorobenzene was purchased from Sigma Aldrich and used without purification. Other chemicals were purchased from Sigma Aldrich, Fluorochem or Alfa Aeser.

2. Synthetic procedures

Compounds **5**, **6** and **9** are all known literature compounds which were synthesised by the following general procedure: LH (0.60 mmol) was dissolved in hexane and added dropwise to a solution of trimethylamine alane (0.72 mmol, 63 mg) in hexane at room temperature. The reaction was stirred at 298 K overnight, before the solvent was removed *in vacuo*. The crude mixture was recrystallised in hot hexane. The reaction was filtered, and the resultant solid was dried *in vacuo* and isolated as colourless crystals.

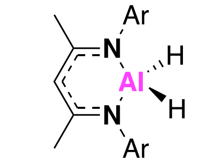

Ar = ph, mes, dep

2: A slight excess of compound **A** (17 mg, 0.038 mmol) in benzene-d_6 (0.6 mL) was added to **1** (15 mg, 0.03 mmol) and the solution was transferred to a JY NMR tube. The reaction was monitored by ^{1}H NMR spectroscopy and after <15 minutes complete consumption **1** was observed, along with the formation of the intermediate **2**. Excess **A** remained present in solution and the sample could not be cleanly isolated due to the complex equilibrium in play. As such, the NMR analysis was conducted of samples formed in situ. Crystals could be grown from the sample, but repeated attempts to collect SCXRD showed severe twinning intrinsic to the crystal, possibly indicting that the sample crystallises as a mixture **2** and alternative reaction products **3** and **B**.

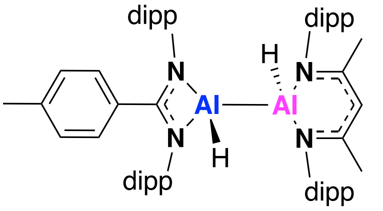

^{1}H NMR (500 MHz, benzene-d_6, 298 K): 0.95 (d, 6H, CH(C*H*$_3$), $^3J_{HH}$ = 6.8 Hz), 1.02 (d, 6H, CH(C*H*$_3$), $^3J_{HH}$ = 6.8 Hz), 1.03 (d, 6H, CH(C*H*$_3$), $^3J_{HH}$ = 6.8 Hz), 1.11 (d, 6H, CH(C*H*$_3$), $^3J_{HH}$ = 6.8 Hz), 1.14 (d, 6H, CH(C*H*$_3$), $^3J_{HH}$ = 6.8 Hz), 1.17 (d, 6H, CH(C*H*$_3$), $^3J_{HH}$ = 6.8 Hz), 1.23 (d, 6H, CH(C*H*$_3$), $^3J_{HH}$ = 6.8 Hz), 1.26 (d, 6H, CH(C*H*$_3$), $^3J_{HH}$ = 6.8 Hz), 1.48 (s, 6H, C(C*H*$_3$)CHC(C*H*$_3$)), 1.61 (s, 3H, *p*-C*H*$_3$), 3.43 (sept, 2H, C*H*(CH$_3$)$_2$, $^3J_{HH}$ = 6.8 Hz), 3.47 (sept, 2H, C*H*(CH$_3$)$_2$, $^3J_{HH}$ = 6.8 Hz), 3.56 (sept, 2H, C*H*(CH$_3$)$_2$, $^3J_{HH}$ = 6.8 Hz), 3.62 (sept, 2H, C*H*(CH$_3$)$_2$, $^3J_{HH}$ = 6.8 Hz), 4.54 (bs, 1H, Al–H), 5.01 (s, 1H, C(CH$_3$)C*H*C(CH$_3$)), 5.09 (bs, 1H, Al–H), 6.36 (d, 2H, Ar*H*, $^3J_{HH}$ = 5.0 Hz), 6.95-7.14 (m, 14H, Ar*H*). ^{13}C{^{1}H} NMR (125 MHz, benzene-d_6, 298 K): 20.7 (*p*-*C*H$_3$), 23.0 (CH(*C*H$_3$)$_2$), 23.2 (*C*H$_3$), 23.5 (CH(*C*H$_3$)$_2$), 23.7 (CH(*C*H$_3$)$_2$), 24.3 (CH(*C*H$_3$)$_2$), 24.7 (CH(*C*H$_3$)$_2$), 25.0 (CH(*C*H$_3$)$_2$), 25.4 (CH(*C*H$_3$)$_2$), 26.6 (CH(*C*H$_3$)$_2$), 28.1 (*C*H(CH$_3$)$_2$), 28.3 (*C*H(CH$_3$)$_2$), 28.7 (*C*H(CH$_3$)$_2$), 29.1 (*C*H(CH$_3$)$_2$), 98.1 (C(CH$_3$)*C*HC(CH$_3$)), 123.6 (*C*H), 123.7 (*C*H), 124.0 (*C*H), 124.5 (*C*H), 125.5 (*C*H), 127.0 (*C*H), 128.0 (*C*H), 130.5 (*C*H), 140.1 (C^{IV}), 140.6 (C^{IV}), 142.6 (C^{IV}), 143.0 (C^{IV}), 143.4 (C^{IV}), 143.9 (C^{IV}), 145.3 (C^{IV}), 170.4 (N*C*(Ar)N), 170.4 (*C*(CH$_3$)CH*C*(CH$_3$)).

3: Compound **A** (15 mg, 0.03 mmol) in benzene-d_6 (0.6 mL) was added to **1** (32.6 mg, 0.66 mmol) and the solution was transferred to a JY NMR tube. The reaction was monitored by ^{1}H NMR spectroscopy and after 24 hours complete consumption of the starting materials and intermediate was observed. The solvent was removed in vacuo and hexane (1 mL) was added and the product formed as colourless crystals at room temperature (20 mg, 62%). NMR analysis was consistent with previously reported data for this compound, where the compound was prepared by an alternate synthetic method.[4]

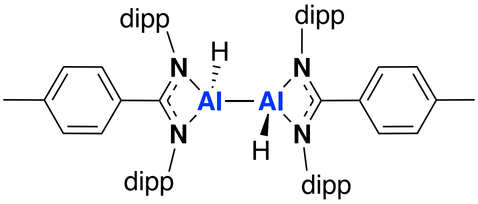

4: An excess of compound **A** (28.7 mg, 0.06 mmol) in benzene-d_6 (0.6 mL) was added to **1** (24 mg, 0.05 mmol) and the solution was transferred to a JY NMR tube. The reaction was monitored by ^{1}H NMR spectroscopy and after <15 minutes complete consumption **1** was observed, along with the formation of the intermediate **2**. The sample was heated at 80 °C and monitored periodically by ^{1}H NMR spectroscopy; after 5 days complete consumption of the intermediate was observed. The solvent was removed in vacuo, hexane (1 mL) was added and the sample was recrystallized by slow evaporation at 25 °C, with crystals of **4** forming selectively. The bright red crystals were isolated and dried in vacuo (11 mg, 45 %).

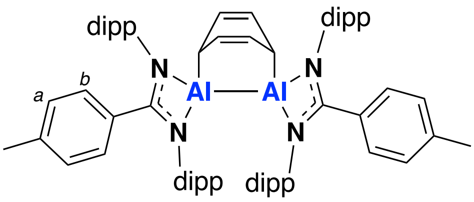

^{1}H NMR (500 MHz, benzene-d_6, 298 K): 0.41 (d, 6H, CH(C*H*$_3$), $^3J_{HH}$ = 6.8 Hz), 0.51 (d, 6H, CH(C*H*$_3$), $^3J_{HH}$ = 6.8 Hz), 0.84 (d, 6H, CH(C*H*$_3$), $^3J_{HH}$ = 6.8 Hz), 1.24 (d, 6H, CH(C*H*$_3$), $^3J_{HH}$ = 6.8 Hz), 1.33 (d, 6H, CH(C*H*$_3$), $^3J_{HH}$ = 6.8 Hz), 1.41 (d, 6H, CH(C*H*$_3$), $^3J_{HH}$ = 6.8 Hz), 1.50 (d, 6H, CH(C*H*$_3$), $^3J_{HH}$ = 6.8 Hz), 1.51 (d, 6H, CH(C*H*$_3$), $^3J_{HH}$ = 6.8 Hz), 1.63 (s, 6H, p-C*H*$_3$), 3.22 (sept, 4H, C*H*(CH$_3$)$_2$, $^3J_{HH}$ = 6.8 Hz), 3.94 (sept, 2H, C*H*(CH$_3$)$_2$, $^3J_{HH}$ = 6.8 Hz), 4.07 (sept, 2H, C*H*(CH$_3$)$_2$, $^3J_{HH}$ = 6.8 Hz), 6.36 (d, 4H, Ar*H*^{a}, $^3J_{HH}$ = 8.4 Hz), 6.84 (d, 2H, Ar*H*, $^3J_{HH}$ = 7.5 Hz), 6.97 (d, 4H, Ar*H*^{b}, $^3J_{HH}$ = 8.4 Hz), 7.0 (t, 2H, Ar*H*, $^3J_{HH}$ = 7.5 Hz), 7.06 (d, 2H, Ar*H*, $^3J_{HH}$ = 7.5 Hz), 7.12 (d, 2H, Ar*H*, $^3J_{HH}$ = 7.5 Hz), 7.20-7.28 (m, 4H, Ar*H*). ^{2}H NMR (500 MHz, benzene-d_6, 298 K): 2.68 (bs, C*D*), 5.35 (bs, C*D*); ^{13}C{^{1}H} NMR (125 MHz, benzene-d_6, 298 K): 20.8 (*p*-*C*H$_3$), 22.7 (CH(*C*H$_3$)$_2$), 23.2(CH(*C*H$_3$)$_2$), 23.8 (CH(*C*H$_3$)$_2$), 24.2 (CH(*C*H$_3$)$_2$), 24.4 (CH(*C*H$_3$)$_2$), 25.4 (CH(*C*H$_3$)$_2$), 25.9 (CH(*C*H$_3$)$_2$), 26.4 (CH(*C*H$_3$)$_2$), 28.1 (*C*H(CH$_3$)$_2$), 28.3 (*C*H(CH$_3$)$_2$), 28.6 (*C*H(CH$_3$)$_2$), 28.7 (*C*H(CH$_3$)$_2$), 123.4 (*C*H), 123.9 (*C*H), 124.2 (*C*H), 124.4 (*C*H), 125.7 (*C*H), 126.2 (*C*H), 126.8 (*C*H), 128.3 (*C*H), 128.5 (*C*H^a), 130.5 (*C*H^b), 139.3 (C^{IV}), 139.5 (C^{IV}), 140.6 (C^{IV}), 143.3 (C^{IV}), 143.6 (C^{IV}), 144.4 (C^{IV}), 145.3 (C^{IV}), 172.1 (N*C*(Ar)N). *Note: Alkane and alkene signals of the benzene-d_6 fragment are in intra-/inter-molecular exchange and can be observed as two broad singlets in the ^{2}H NMR spectrum, but no signals were observed in the ^{13}C{^{1}H} NMR spectrum.*

Anal. Calc. ($Al_2C_{70}H_{88}N_4$): C, 80.88; H, 8.53; N, 5.39. Found: C, 80.36; H, 8.65; N, 5.27.

7: Compound **A** (30 mg, 0.07 mmol) in benzene-d_6 (0.6 mL) was added to **5** (48.9 mg, 0.04 mmol) and the solution was transferred to a JY NMR tube. The reaction was monitored by ^{1}H NMR spectroscopy and after 3 hours complete consumption of the starting materials and intermediate was observed. The solvent was removed in vacuo and hexane (1 mL) was added. The solution was left to slowly evaporate at 25 °C, which afforded yellow crystals (37 mg, 76%).

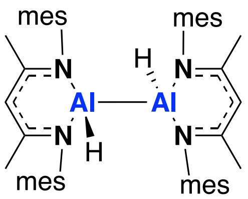

^{1}H NMR (500 MHz, benzene-d_6, 298 K): 1.48 (s, 12 H, C*H*$_3$), 2.10 (s, 12 H, C*H*$_3$), 2.17 (s, 12 H, C*H*$_3$), 2.41 (s, 12 H, C*H*$_3$), 4.70 (bs, 2H, Al–*H*), 4.80 (s, 2H, C(CH$_3$)C*H*C(CH$_3$)), 6.74 (s, 4H, Ar*H*), 6.75 (s, 4H, Ar*H*). ^{13}C{^{1}H} NMR (125 MHz, benzene-d_6, 298 K): 18.9 (*C*H$_3$), 19.1 (*C*H$_3$), 20.8 (*C*H$_3$), 22.3 (*C*H$_3$), 96.1 (C(CH$_3$)*C*HC(CH$_3$)), 129.5 (*C*H), 129.7 (*C*H), 133.9 (C^{IV}), 134.8 (C^{IV}), 141.8 (C^{IV}), 168.2 (*C*(CH$_3$)CH*C*(CH$_3$)).

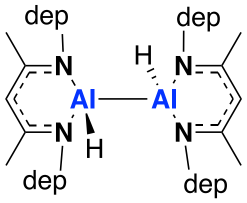

8: Compound **A** (23.3 mg, 0.05 mmol) in benzene-d_6 (0.6 mL) was added to **6** (41 mg, 0.1 mmol) and the solution was transferred to a JY NMR tube. The reaction was monitored by ^{1}H NMR spectroscopy and after 1 hour complete consumption of the starting materials and intermediate was observed. The solvent was removed in vacuo and hexane (1 mL) was added. The solution was left to stand at 25 °C and crystals of **B** formed. The reaction was filtered and the filtrate stored in a freezer at -35 °C, where the product precipitated as orange crystals (16 mg, 40%).

^{1}H NMR (400 MHz, benzene-d_6, 298 K): 1.10 (t, 12H, $CH_2C\mathit{H}_3$, $^3J_{HH}$ = 7.6 Hz), 1.29 (t, 12H, $CH_2C\mathit{H}_3$, $^3J_{HH}$ = 7.6 Hz), 1.44 (s, 12H, $C\mathit{H}_3$), 2.67 (m, 4H, $C\mathit{H}_2CH_3$), 2.75 (m, 4H, $C\mathit{H}_2CH_3$), 3.00 (m, 4H, $C\mathit{H}_2CH_3$), 3.09 (m, 4H, $C\mathit{H}_2CH_3$), 4.59 (bs, 2H, Al–*H*), 4.72 (s, 2H, $C(CH_3)C\mathit{H}C(CH_3)$), 7.03 (m, 12H, Ar*H*). $^{13}C\{^1H\}$ NMR (125 MHz, benzene-d_6, 298 K): 13.8 ($CH_2\mathit{C}H_3$), 14.0 ($CH_2\mathit{C}H_3$), 22.4 ($\mathit{C}H_3$), 23.8 ($\mathit{C}H_2CH_3$), 24.5 ($\mathit{C}H_2CH_3$), 96.1 ($C(CH_3)\mathit{C}HC(CH_3)$), 125.3 (*C*H), 125.9 (*C*H), 126.2 (*C*H), 138.6 (C^{IV}), 139.2 (C^{IV}), 142.8 (C^{IV}), 167.9 ($\mathit{C}(CH_3)CH\mathit{C}(CH_3)$).

Anal. Calc. ($Al_2C_{50}H_{68}N_4$): C, 78.08; H, 8.80; N, 7.19. Found: C, 77.03; H, 8.81; N, 7.07.

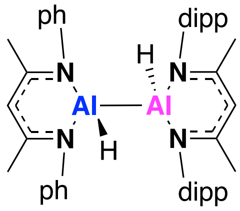

10: Compound **A** (23 mg, 0.05 mmol) in benzene-d_6 (0.6 mL) was added to **9** (14.4 mg, 0.05 mmol) and the solution was transferred to a JY NMR tube. The reaction was monitored by ^{1}H NMR spectroscopy and after <15 minutes complete consumption of the starting material was observed. The solvent was removed in vacuo and the orange solid was recrystallised from a mixture of toluene and hexane at -35 °C (34 mg, 91%).

^{1}H NMR (500 MHz, benzene-d_6, 298 K): 1.15 (d, 6H, $CH(C\mathit{H}_3)$, $^3J_{HH}$ = 6.8 Hz), 1.25 (d, 6H, $CH(C\mathit{H}_3)$, $^3J_{HH}$ = 6.8 Hz), 1.31 (d, 6H, $CH(C\mathit{H}_3)$, $^3J_{HH}$ = 6.8 Hz), 1.36 (d, 6H, $CH(C\mathit{H}_3)$, $^3J_{HH}$ = 6.8 Hz), 1.49 (s, 6H, $C\mathit{H}_3$), 1.70 (s, 6H, $C\mathit{H}_3$), 3.62 (sept, 4H, $C\mathit{H}(CH_3)_2$, $^3J_{HH}$ = 6.8 Hz), 4.59 (bs, 1H, Al–*H*), 5.03 (s, 1H, $C(CH_3)C\mathit{H}C(CH_3)$), 5.13 (s, 1H, $C(CH_3)C\mathit{H}C(CH_3)$), 5.13 (bs, 1H, Al–*H*), 6.05 (d, 4H, Ar*H*, $^3J_{HH}$ = 7.7 Hz), 6.90-7.05 (m, 6H, Ar*H*), 7.10-7.28 (m, 6H, Ar*H*). $^{13}C\{^1H\}$ NMR (125 MHz, benzene-d_6, 298 K): 22.8 ($\mathit{C}H_3$), 23.5 ($\mathit{C}H_3$), 23.5 ($CH(\mathit{C}H_3)$), 24.4 ($CH(\mathit{C}H_3)$), 24.6 ($CH(\mathit{C}H_3)$), 27.0 ($CH(\mathit{C}H_3)$), 28.0 ($CH(\mathit{C}H_3)_2$), 29.3 ($\mathit{C}H(CH_3)_2$), 98.3 ($C(CH_3)\mathit{C}HC(CH_3)$), 103.1 ($C(CH_3)\mathit{C}HC(CH_3)$), 124.1 (*C*H), 124.5 (*C*H), 124.6 (*C*H), 125.8 (*C*H), 127.0 (*C*H), 128.9 (*C*H), 143.6 (C^{IV}), 143.6 (C^{IV}), 146.2 (C^{IV}), 146.9 (C^{IV}), 166.7 ($\mathit{C}(CH_3)CH\mathit{C}(CH_3)$), 170.0 ($\mathit{C}(CH_3)CH\mathit{C}(CH_3)$).

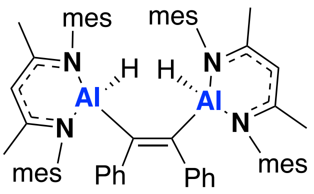

11: Compound **7** (20 mg, 0.03 mmol) was dissolved in benzene-d_6 (0.6 mL), diphenylacetylene (5 mg, 0.03 mmol) was added and the solution was transferred to a JY NMR tube. The reaction was heated at 80 °C for 1 hour, after which time the orange solution had turned colourless and the ^{1}H NMR spectrum showed complete consumption of **7**. The solvent was removed in vacuo, and the resultant solid washed with hexane yielding the product as a white powder which could be recrystallised from a mixture of toluene and hexane at -35 °C (20mg, 80%).

^{1}H NMR (400 MHz, benzene-d_6, 298 K): 1.39 (s, 12 H, CH_3), 1.97 (s, 12 H, CH_3), 2.12 (s, 12 H, CH_3), 2.20 (s, 12 H, CH_3), 3.77 (bs, 2H, Al–H), 5.03 (s, 2H, C(CH$_3$)CHC(CH$_3$)), 6.46 (s, 4H, Ar^{mes}H), 6.76 (s, 4H, CH^{mes}), 6.95 (2H, m, C$H^{p\text{-}Ph}$), 6.71 (4H, t, C$H^{m\text{-}Ph}$, $^{3}J_{\mathrm{HH}}$ = 8.0 Hz), 7.26 (4H, m, C$H^{o\text{-}Ph}$). ^{13}C{^{1}H} NMR (125 MHz, benzene-d_6, 298 K): 19.0 (CH$_3$), 19.3 (CH$_3$), 20.7 (CH$_3$), 22.7 (CH$_3$), 94.8 (C(CH$_3$)CHC(CH$_3$)), 123.0 (CH^{Ph}), 126.9 (CH^{Ph}), 129.1 (CH^{mes}), 129.1 (CH^{mes}), 130.4 (CH^{Ph}), 132.8 (C^{IV}), 134.1 (C^{IV}), 141.9 (C^{IV}), 150.7 (C^{IV}), 169.2 (C(CH$_3$)CHC(CH$_3$)).

12 a-c: Compound **7** (11 mg, 0.015 mmol) was dissolved in benzene-d_6 (0.6 mL) and the solution was transferred to a JY NMR tube. Hexafluorobenzene (5.2 µL, 0.045 mmol) was added and the reaction was heated at 80 °C for 30 minutes. The sample was analysed by ^{1}H and ^{19}F NMR spectroscopy and found to contain a mixture of products which have been assigned as **12 a-c** and **5**. Other minor unidentified products are also found to be present. Attempts to isolate single crystals have thus far been unsuccessful.

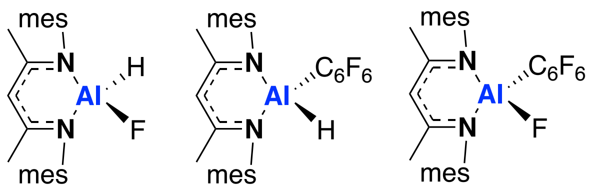

12a **12b** **12c**

Selected NMR data: **12a:** ^{19}F NMR (400 MHz, benzene-d_6, 298 K): -157.6 (bd, 1F, Al–F, $^{2}J_{\mathrm{HF}}$ = 62 Hz); **12b:** ^{19}F NMR (400 MHz, benzene-d_6, 298 K): -119.6 (m, 2F, o-C$_6$F$_5$), -153.7 (m, 1F, p-C$_6$F$_5$), -161.8 (m, 2F, m-C$_6$F$_5$); **12c:** ^{19}F NMR (400 MHz, benzene-d_6, 298 K): -118.7 (m, 2F, o-C$_6$F$_5$), -152.6 (m, 1F, p-C$_6$F$_5$), -162.3 (bs, 1F, Al–F),. Note: m-C$_6$F$_5$ hidden by **2b**. m/z (EI, +ve): **12a** + K, 417; **12b** + K, 567; **12c** + K, 483.

3. Supporting Figures

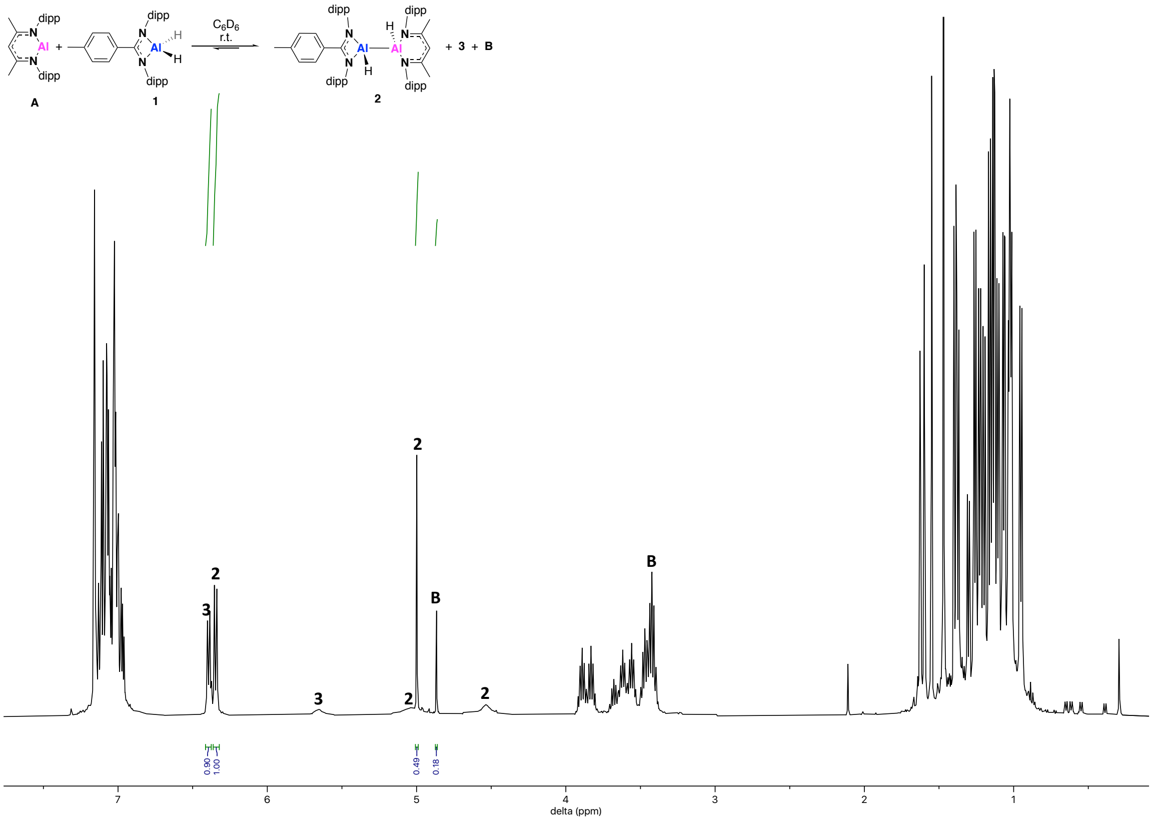

Figure S1: The stoichiometric reaction of **A** and **1** at 25 °C in benzene-d$_6$.

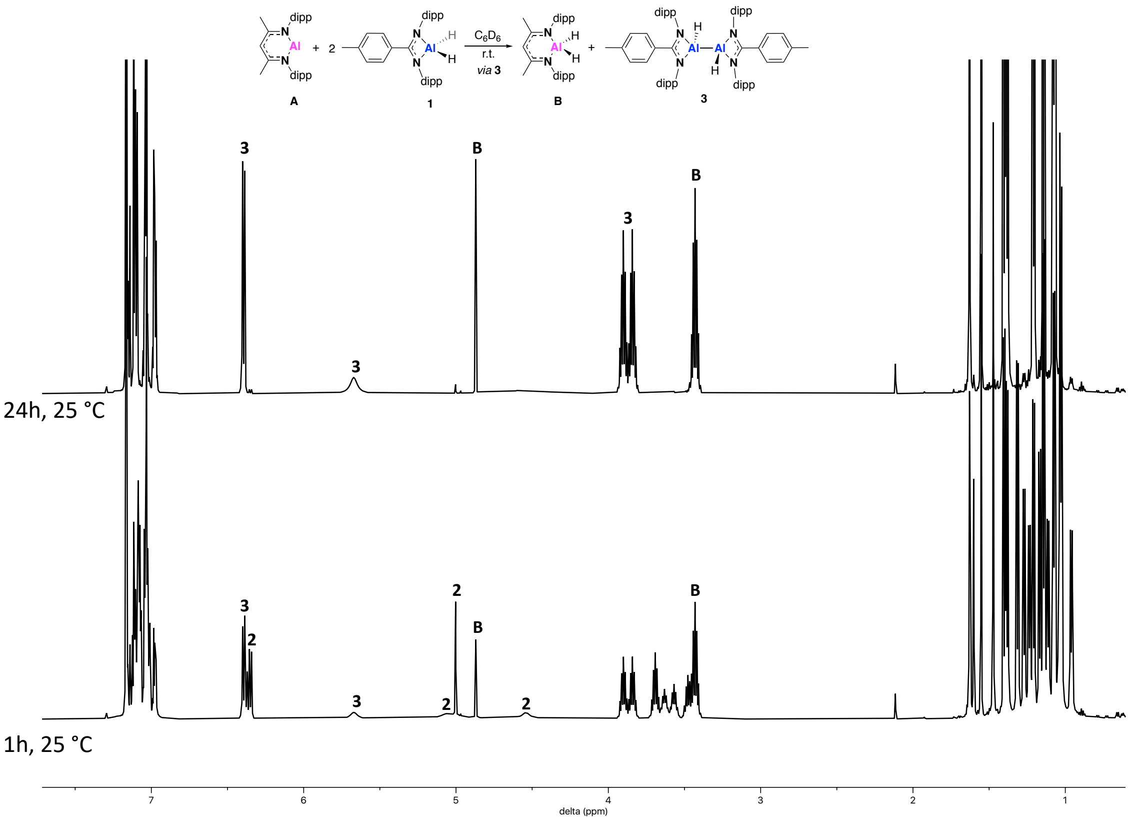

Figure S2: The 1:2 reaction of **A** and **1** at 25 °C in benzene-d$_6$.

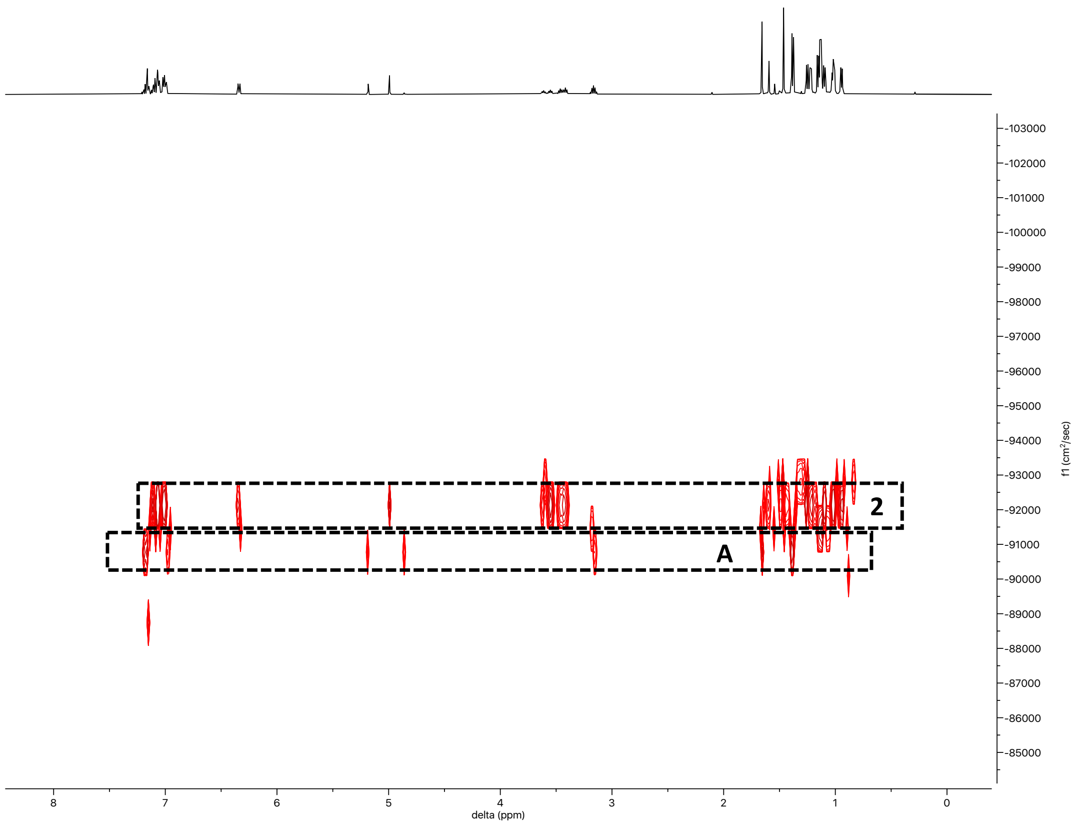

Figure S3: The DOSY NMR spectrum of **2** with an excess of **A** at 25 °C in benzene-d$_6$.

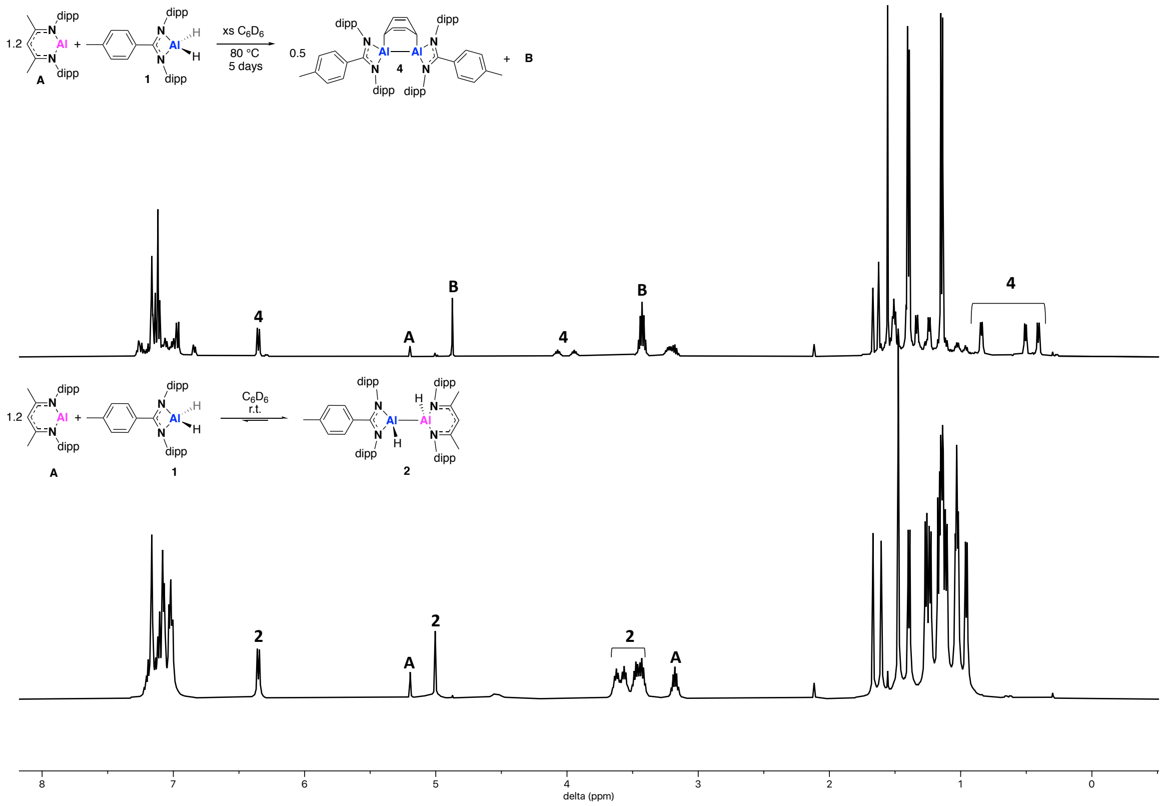

Figure S4: The reaction of **1** with an excess of **A** in at 25 °C in benzene-d$_6$ (bottom) and after 5 days at 80 °C (top).

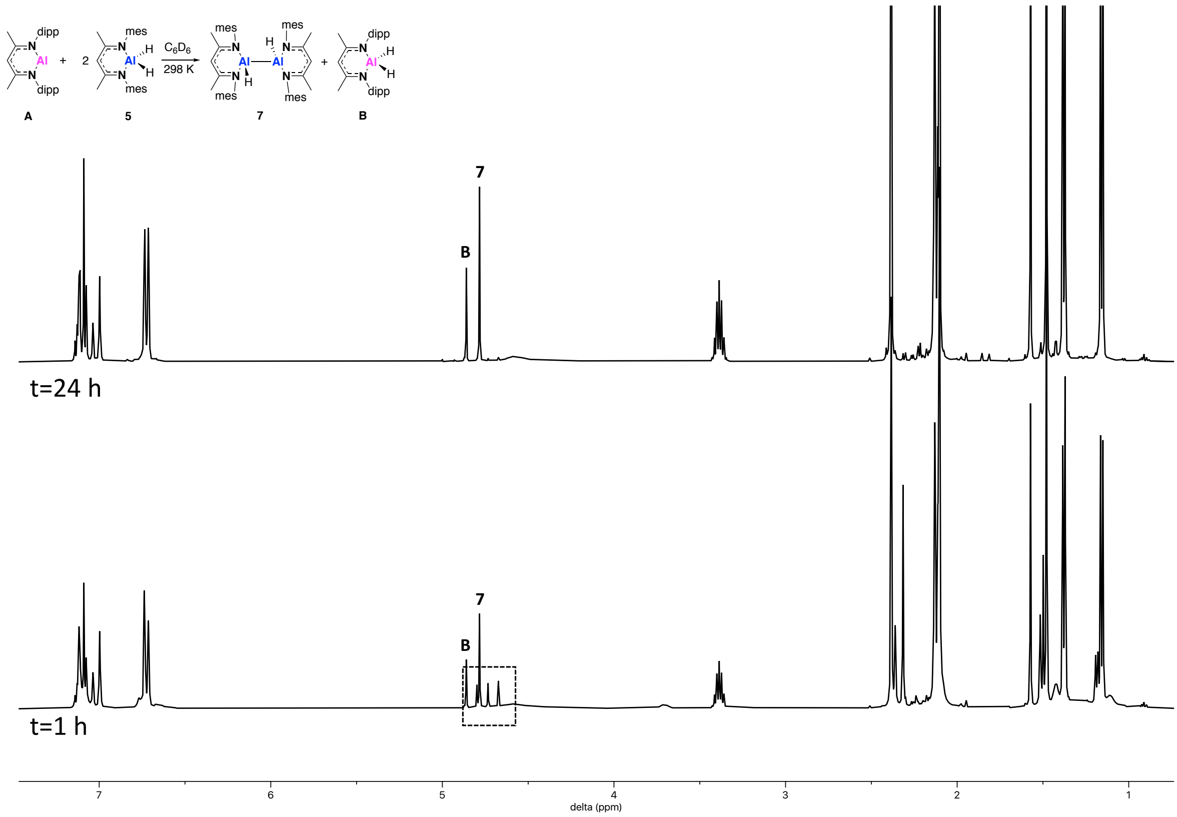

Figure S5: The reaction of **A** with 2 equiv. **5** at 25 °C in benzene-d_6 after 1h (bottom) and after 24h (top).

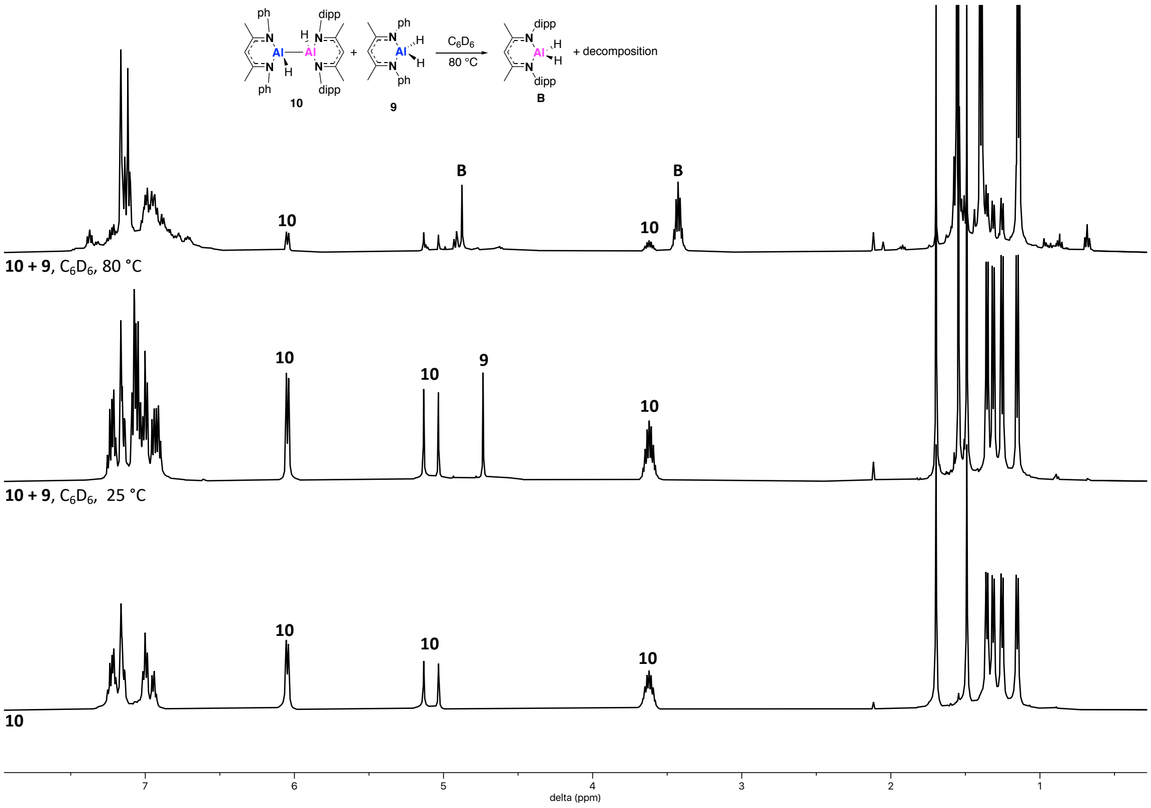

Figure S6: The reaction of **10** with 1 equiv. **9** in benzene-d_6 at 25 °C (bottom) and after 1h at 80 °C (top).

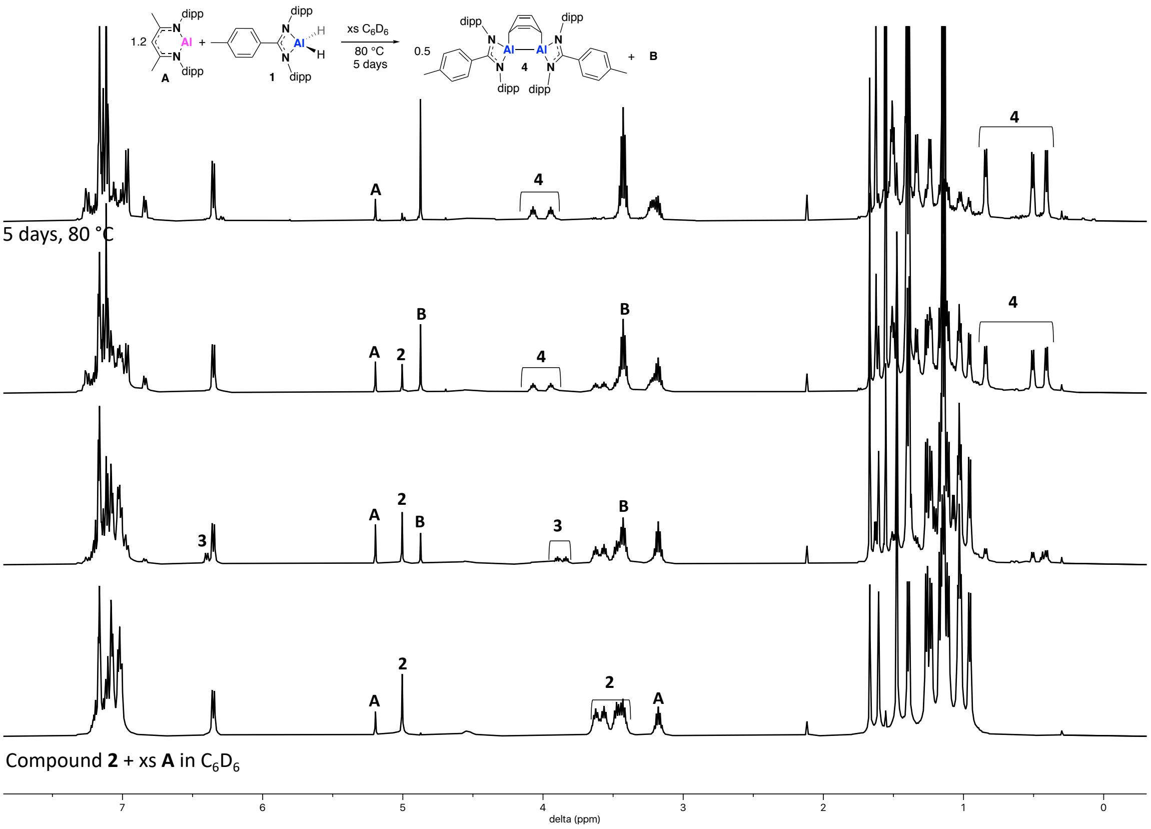

Figure S7: The evolution of the formation of **4** from **A** and **1** at 80 °C in benzene-d_6.

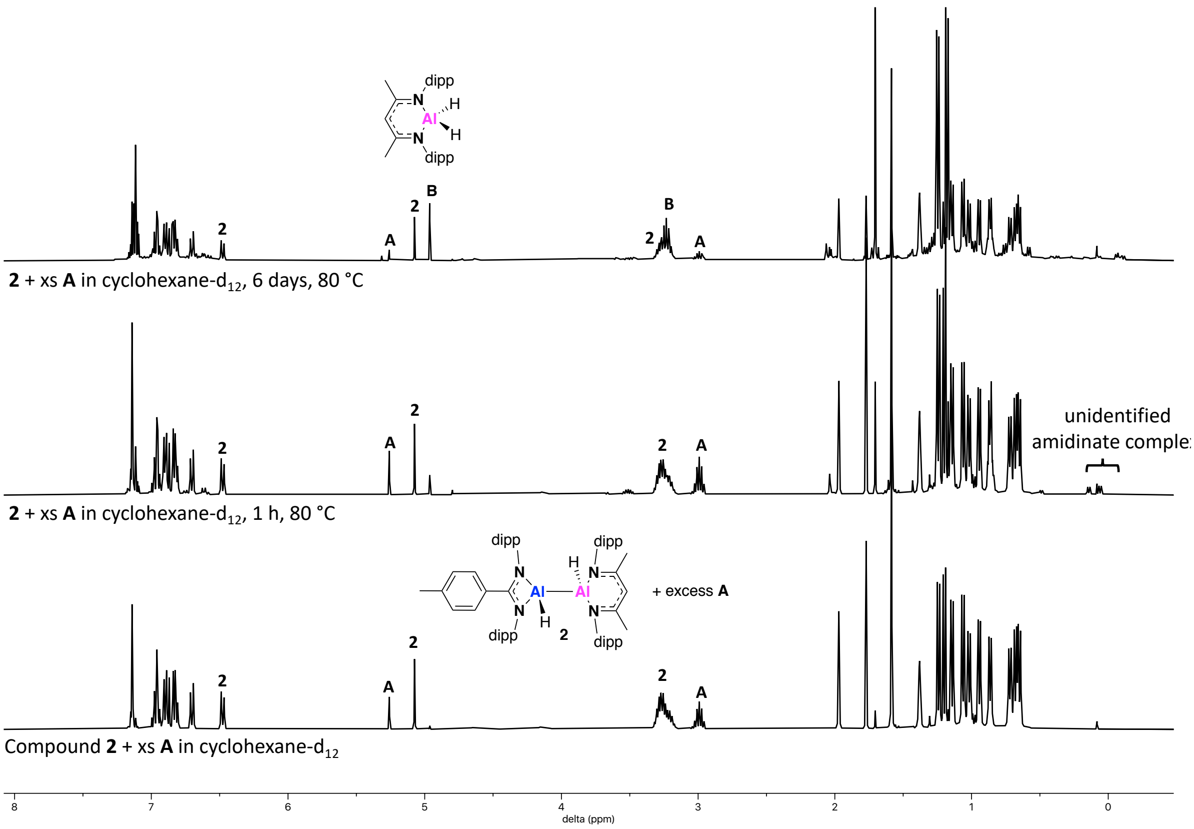

Figure S8: The reaction of **2** with excess **A** at 80°C in cyclohexane-d_{12}.

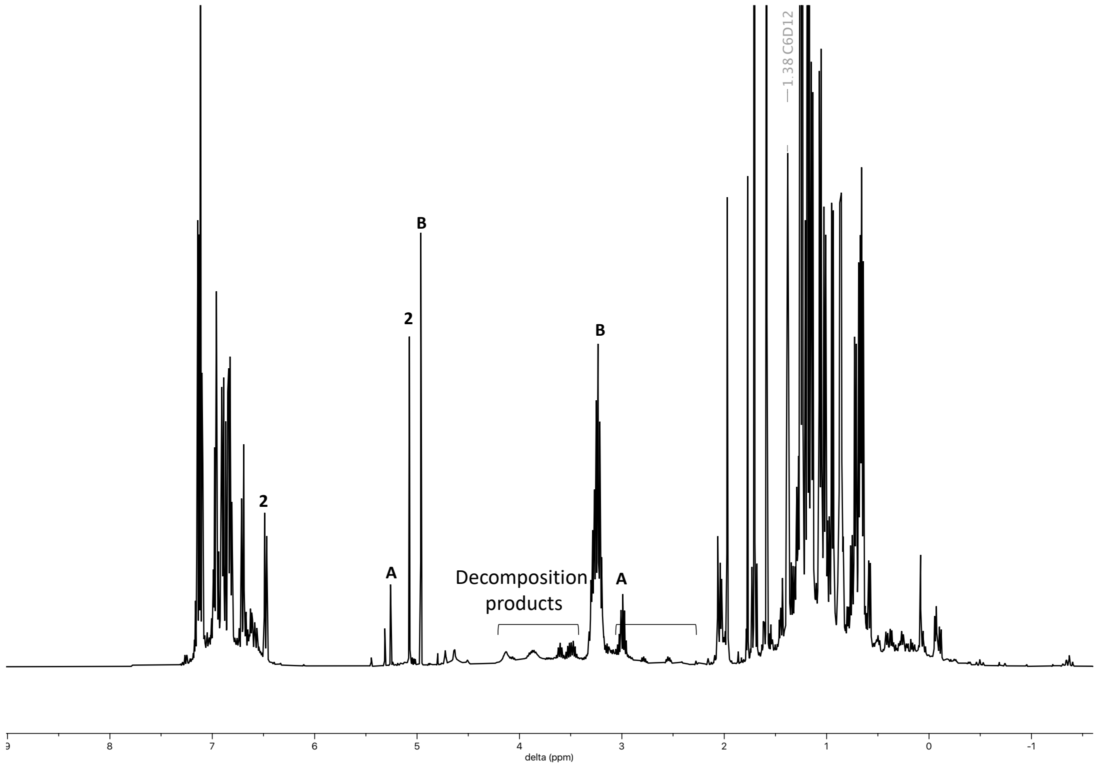

Figure S9: An expanded spectrum of the reaction of **2** with excess **A** at 80°C for 6 days in cyclohexane-d_{12}.

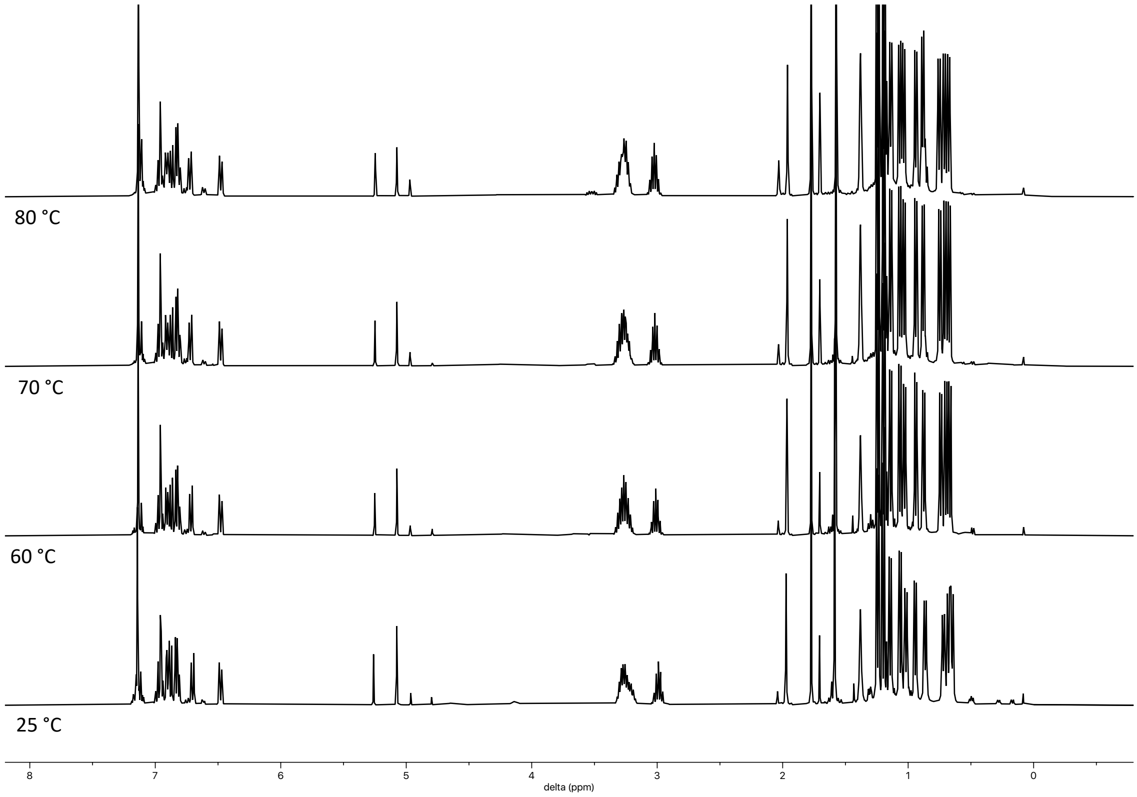

Figure S10: Variable temperature ^{1}H NMR of the reaction of **2** with excess **A** in cyclohexane-d_{12}.

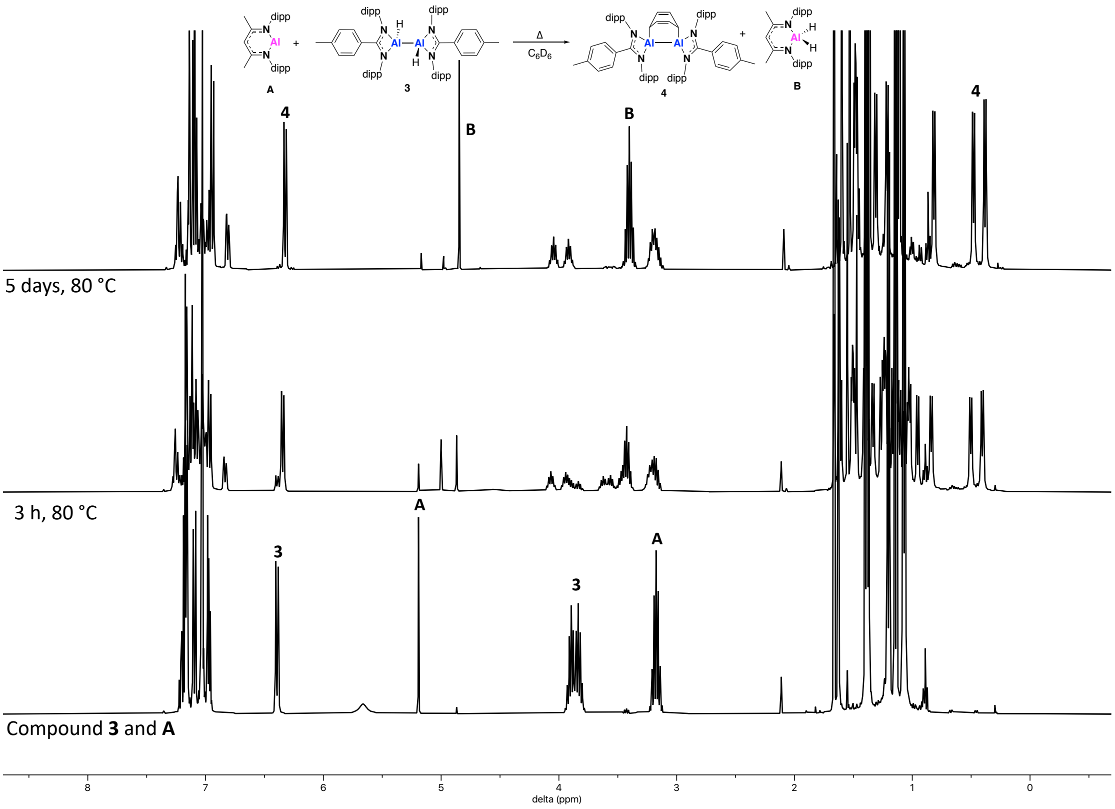

Figure S11: The reaction of compound **3** with **A** at 80°C in benzene-d_6.

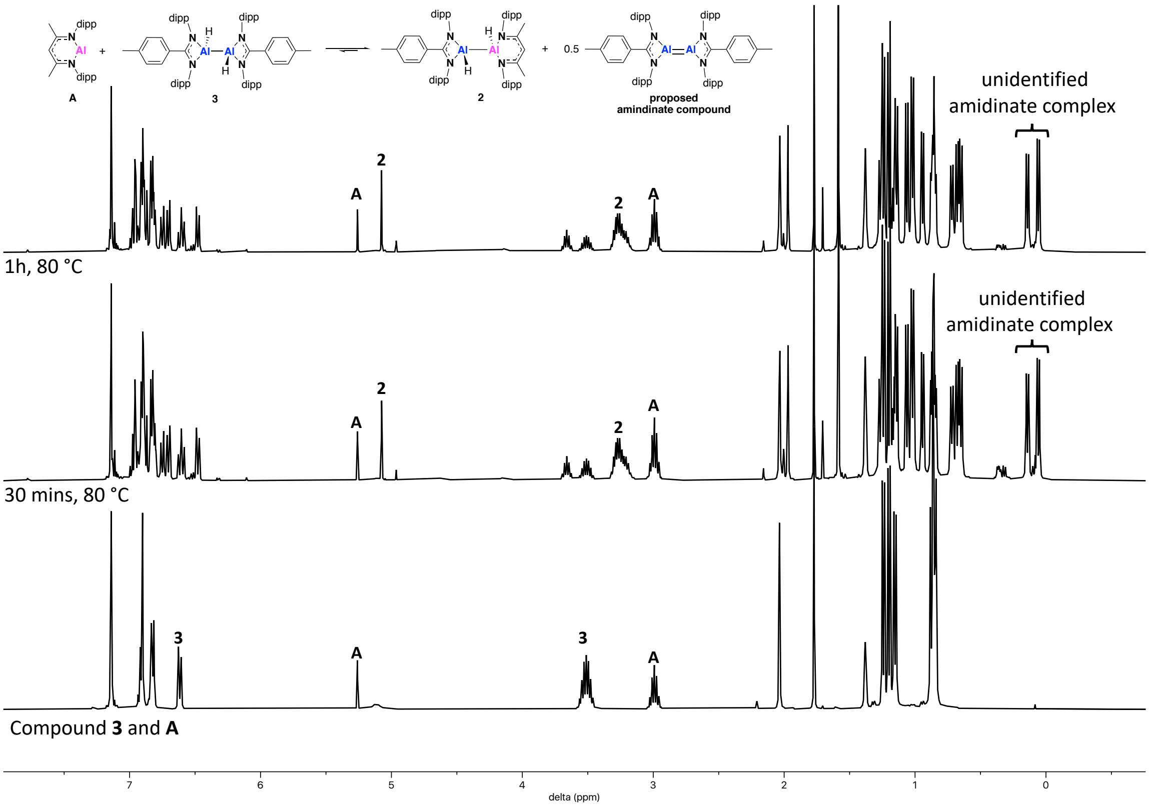

Figure S12: The reaction of compound **3** with **A** at 80°C in cyclohexane-d_{12}.

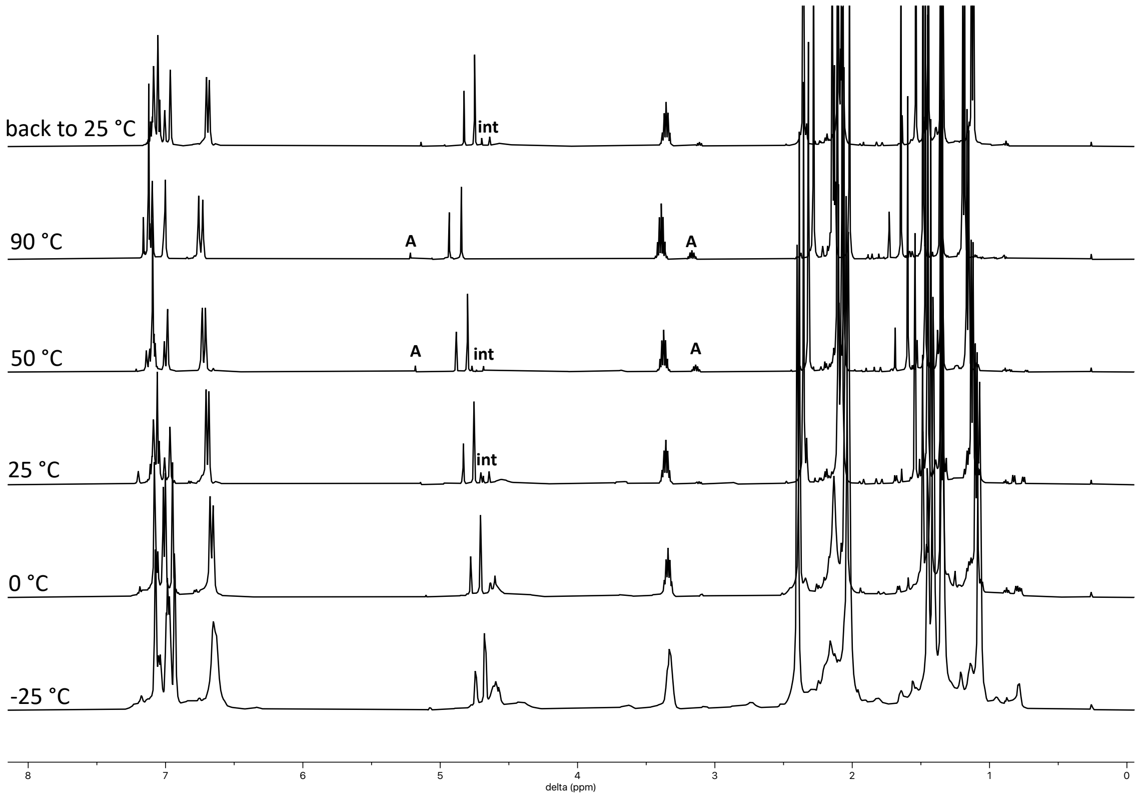

Figure S13: VT ^{1}H NMR spectra of products from the 1:1 reaction of **A** and **5** in toluene-d_8.

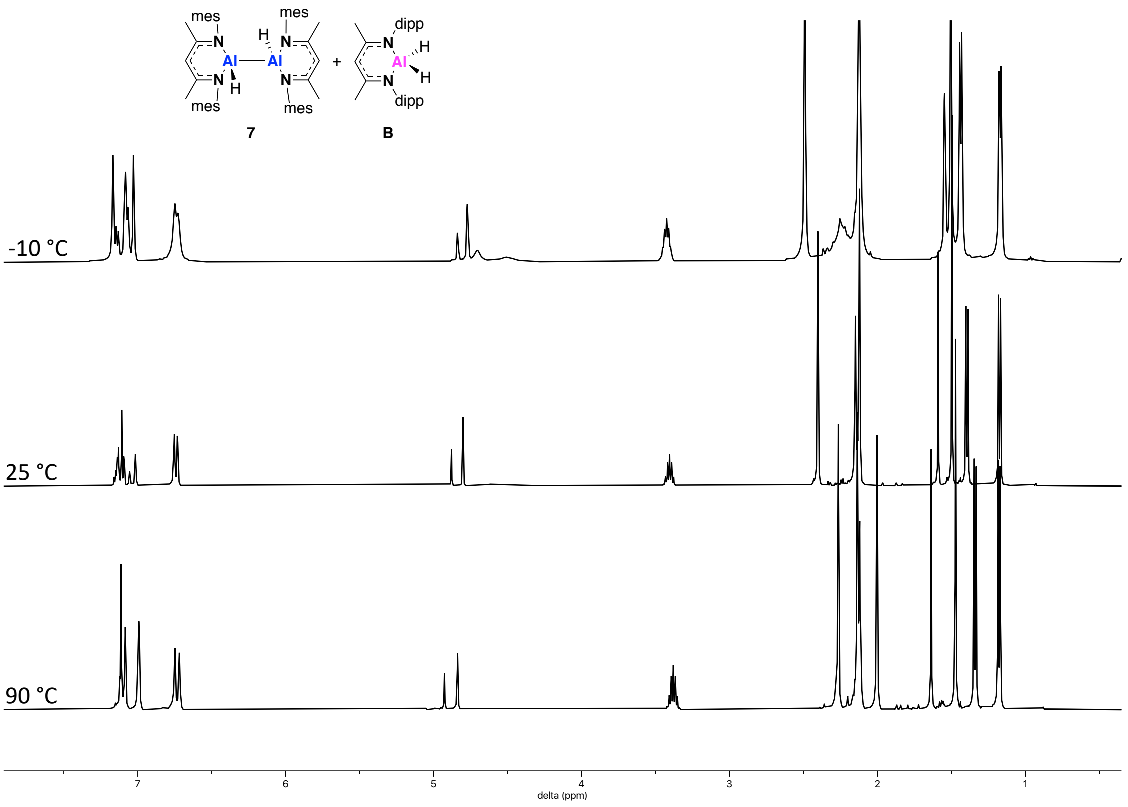

Figure S14: ^{1}H NMR spectra of a 1:1 mix of **7** and **B** toluene-d_8 at 90 °C (bottom), 25 °C (middle) and -10 °C (top).

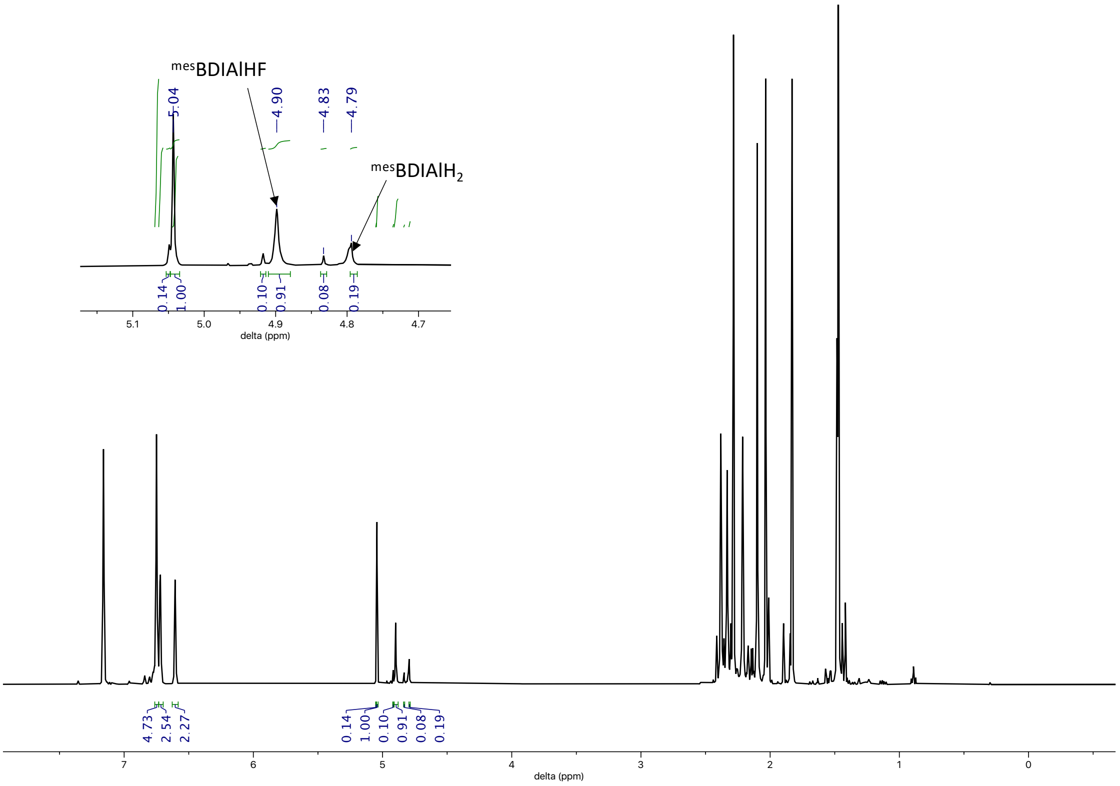

Figure S15: ^{1}H NMR specrum of the reaction of **7** and C_6F_6 in benzene-d6.

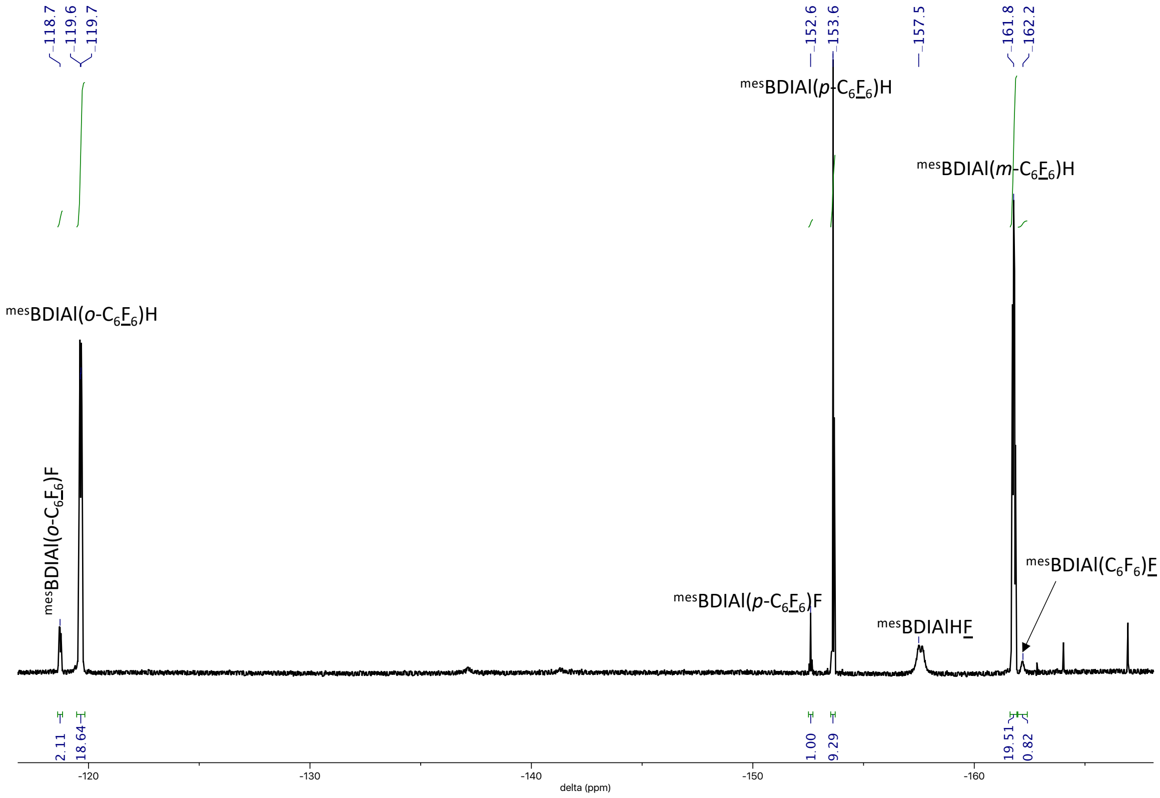

Figure S16: ^{19}F NMR specrum of the reaction of **7** and C_6F_6 in benzene-d6.

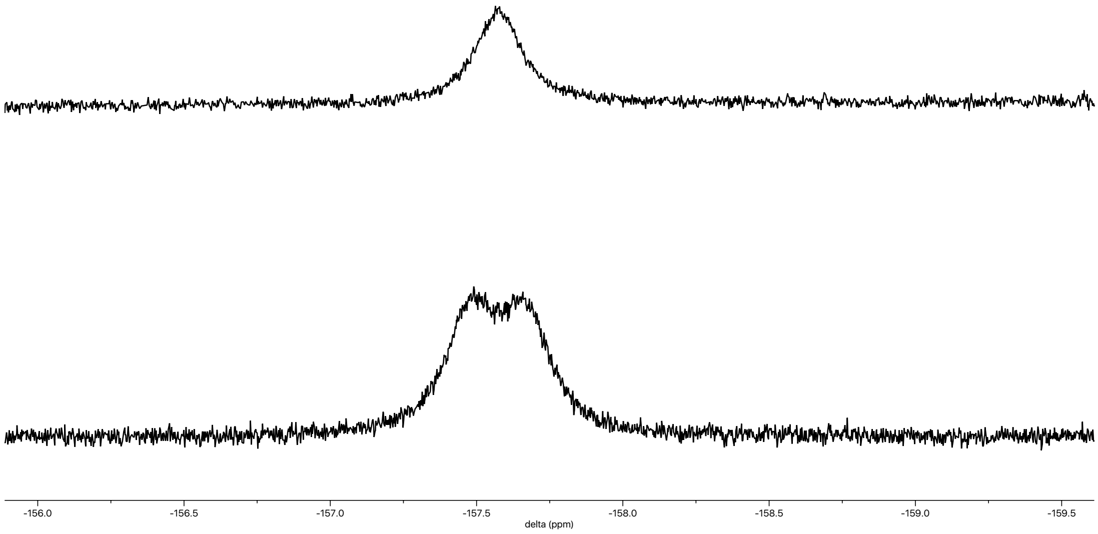

Figure S17: Expanded ^{19}F NMR specrum of the reaction of **7** and C_6F_6 in benzene-d_6, bottom ^{1}H–coupled, top ^{1}H-decoupled.

4. X-ray Crystallographic Data

All crystals were ran on a Agilent Oxford Diffraction SuperNova equipped with a microfocus Cu Kα X-ray source and an Atlas CCD detector. Full spheres of data were collected to 0.84 Å resolution with each 1° scan frame in ω collected twice. Total collection time varied depending on size and quality of crystal, and sample temperature. The Cryojet5® used for these measurement is the original prototype device developed by Oxford Instruments and the Pt-resistance sensor is located in the copper-block heat exchanger and not in the nozzle of the instrument close to the sample (in contrast to the CryojetHT® used in the PXRD experiments). Thus the temperatures quoted in these SXD experiments should be treated as nominal (despite stability to much better than 0.1 °C). Using Olex2,[5] the structure was solved with the ShelXT[6] structure solution program using Intrinsic Phasing and refined with the ShelXL[7] refinement package using Least Squares minimisation.

*The X-ray crystal structure of **4***

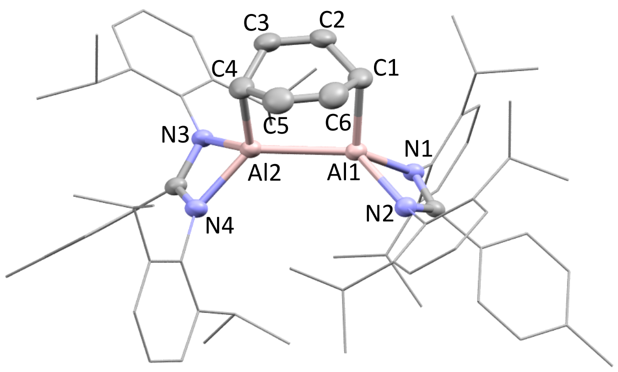

Figure S18: The X-ray crystal structure of **4**. Hydrogen atoms and solvent molecules omitted for clarity.

Single crystals of **4** were grown by slow evaporation from hexane solution. **4** was found to crystallise in a P-1 space group. The unit cell contained a disordered molecule of hexane that was modelled using a solvent mask (SQUEEZE).

Crystal Data for **4:** $C_{70}H_{88}Al_2N_4$, M =1039.40 g/mol, triclinic, space group P-1 (no. 2), a = 13.0837(4) Å, b = 13.5136(3) Å, c = 21.2897(6) Å, α = 75.234(2)°, β = 81.733(2)°, γ = 74.829(2)°, V = 3501.02(17) Å^3, Z = 2, T = 150.01(10) K, μ(CuKα) = 0.656 mm^{-1}, $Dcalc$ = 0.986 g/cm^3, 26972 reflections measured (6.96° ≤ 2Θ ≤ 146.782°), 13646 unique (R_{int} = 0.0378, R_{sigma} = 0.0446) which were used in all calculations. The final R_1 was 0.0477 (I > 2σ(I)) and wR_2 was 0.1392 (all data). CCDC 2155210.

Table S1: Key bond lengths (Å) and bond angles (°) of **4**.

**Al1–Al2**	**Al1–N1**	**Al1–N2**	**Al2–N3**	**Al2–N4**	**Al1–C1**	**Al2–C4**
2.5419(7)	1.948(1)	1.943(2)	1.944(2)	1.949(1)	2.017(2)	2.018(2)
**C1–C2**	**C2–C3**	**C3–C4**	**C4–C5**	**C5–C6**	**C1–C6**	
1.501(3)	1.337(3)	1.498(3)	1.499(3)	1.338(4)	1.498(3)	
**N1-Al1-N2**	**N1-Al1-N2**	**C1-Al1-Al2**	**C4-Al2-Al1**	**C6-C1-C2**	**C3-C4-C5**	
68.3	68.4	93.8	94.4	109.0	109.3	

*The X-ray crystal structure of **7***

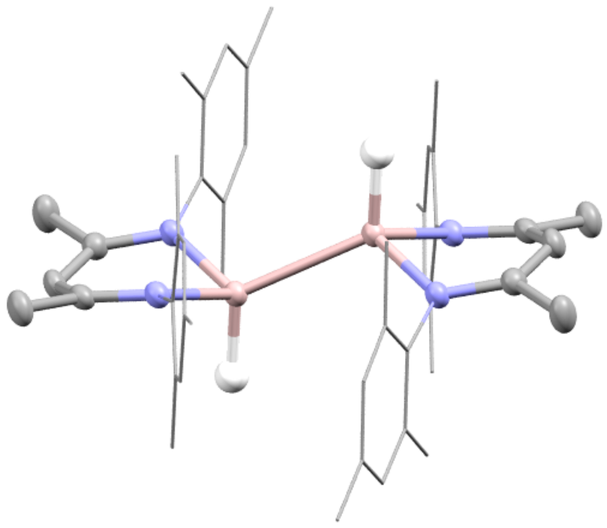

Figure S19: The X-ray crystal structure of **7**. Select hydrogen atoms and solvent molecules omitted for clarity.

Single crystals of **7** were grown by slow evaporation from hexane solution.

Crystal Data for **7:** $C_{46}H_{60}Al_2N_4$, *M* =722.94 g/mol, triclinic, space group P-1 (no. 2), *a* = 8.3105(6) Å, *b* = 10.0476(7) Å, *c* = 13.5868(11) Å, *α* = 71.798(7)°, *β* = 77.181(6)°, *γ* = 72.920(6)°, *V* = 1019.57(14) Å^3, *Z* = 1, *T* = 150.00(10) K, μ(CuKα) = 0.913 mm^{-1}, *Dcalc* = 1.177 g/cm^3, 6985 reflections measured (9.55° ≤ 2Θ ≤ 146.632°), 3953 unique (R_{int} = 0.0332, R_{sigma} = 0.0407) which were used in all calculations. The final R_1 was 0.0471 (I > 2σ(I)) and wR_2 was 0.1386 (all data). CCDC 2155211.

*The X-ray crystal structure of **8***

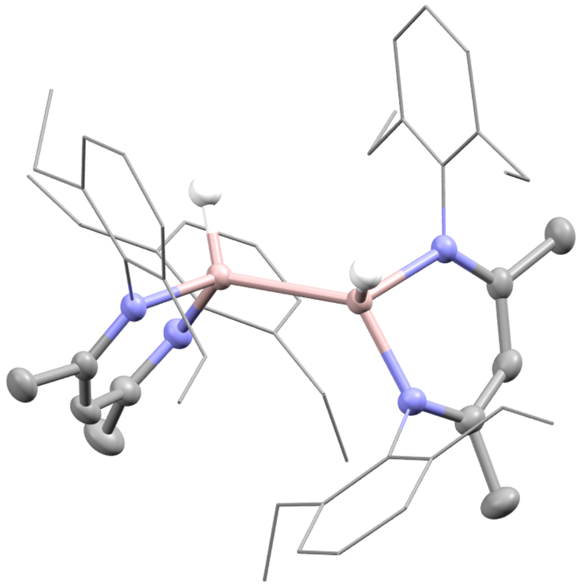

Figure S20: The X-ray crystal structure of **8**. Select hydrogen atoms and solvent molecules omitted for clarity.

Single crystals of **8** were grown by slow evaporation from hexane solution.

Crystal Data for **8:** $C_{50}H_{68}Al_2N_4$, *M* =779.04 g/mol, monoclinic, space group C2/c (no. 15), *a* = 19.0058(3) Å, *b* = 14.4650(2) Å, *c* = 17.1496(2) Å, *β* = 100.0780(10)°, *V* = 4642.00(11) Å^3, *Z* = 4, *T* = 149.97(10) K, μ(CuKα) = 0.833 mm^{-1}, *Dcalc* = 1.115 g/cm^3, 38762 reflections measured (7.726° ≤ 2Θ ≤ 146.934°), 4628 unique (R_{int} = 0.0468, R_{sigma} = 0.0183) which were used in all calculations. The final R_1 was 0.0415 (I > 2σ(I)) and wR_2 was 0.1177 (all data). CCDC 2155212.

*The X-ray crystal structure of **10***

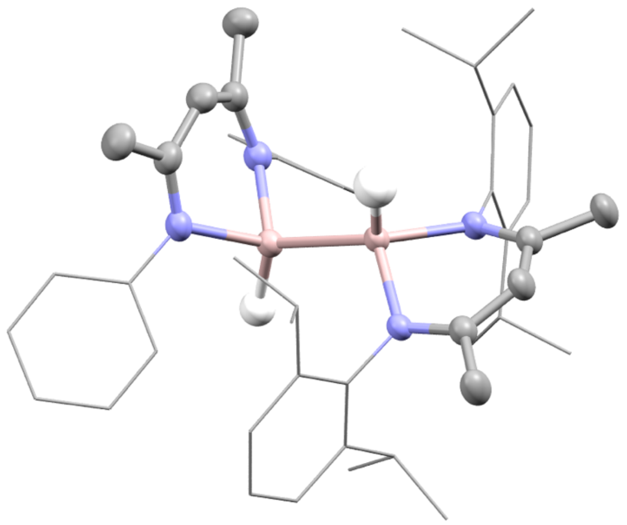

Figure S21: The X-ray crystal structure of **10**. Select hydrogen atoms and solvent molecules omitted for clarity.

Single crystals of **10** were grown from hexane solution at -35 °C.

The structure of **10** was found to have a small amount of unresolved residual electron density near the hydride (H). This was interpreted as co-crystallised **10-OH** and the crystal was modelled as a mixture of **10** (90%) and **10-OH** (10%). It was not possible to locate the H atom of the OH group in the minor component. These atoms were refined isotropically.

Crystal Data for **10:** $C_{46}H_{60.4}Al_2N_4O_{0.1}$, *M* =725.61 g/mol, orthorhombic, space group $P2_12_12_1$ (no. 19), *a* = 12.1525(2) Å, *b* = 16.6789(2) Å, *c* = 20.7945(2) Å, *V* = 4214.84(9) Å^3, *Z* = 4, *T* = 149.9(4) K, μ(CuKα) = 0.888 mm^{-1}, *Dcalc* = 1.143 g/cm^3, 81870 reflections measured (6.794° ≤ 2Θ ≤ 146.992°), 8418 unique (R_{int} = 0.0524, R_{sigma} = 0.0217) which were used in all calculations. The final R_1 was 0.0340 (I > 2σ(I)) and wR_2 was 0.0885 (all data). CCDC 2155213.

Table S2: Table of bond lengths (Å) and bond angles (°) for compound **10**.

Al1–Al2	Al1–N1	Al1–N2	Al2–N3	Al2–N4	N1–Al1–N2	N3–Al2–N4
2.6307(9)	1.940(2)	1.923(2)	1.940(2)	1.947(2)	93.8	91.6

*The X-ray crystal structure of **11***

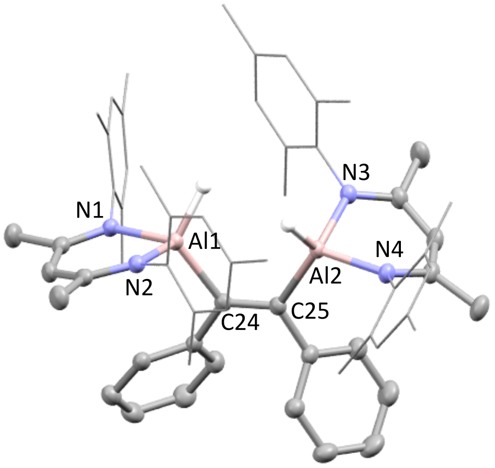

Figure S22: The X-ray crystal structure of **11**. Select hydrogen atoms and solvent molecules omitted for clarity.

Single crystals of **11** were grown from benzene solution at 25 °C.

Crystal Data for **11**: $C_{66}H_{76}Al_2N_4$, M =979.26 g/mol, monoclinic, space group P2$_1$/n (no. 14), a = 12.28210(10) Å, b = 34.0314(2) Å, c = 13.99810(10) Å, β = 103.2620(10)°, V = 5694.85(7) Å^3, Z = 4, T = 151(2) K, μ(CuKα) = 0.781 mm^{-1}, $Dcalc$ = 1.142 g/cm^3, 102293 reflections measured (6.988° ≤ 2Θ ≤ 145.242°), 11223 unique (R_{int} = 0.0351, R_{sigma} = 0.0151) which were used in all calculations. The final R_1 was 0.0540 (I > 2σ(I)) and wR_2 was 0.1334 (all data). CCDC 2155214.

5. Computational Detail

5.1 Computational methods

DFT calculations were run using Gaussian 09. Al centres were described with Stuttgart SDDAll RECPs and associated basis sets and the 6-31G** basis sets were used for all other atoms.[8-10] The functions ωB97X, M062X, M06L and B3PW91 were investigated.

Geometry optimisation calculations were performed without symmetry constraints. Free energies reported within the main text are corrected for the effects of benzene solvent (ε=2.2706) using the using the polarizable continuum model (PCM).[11] In addition, single point dispersion corrections were applied to the ωB97X optimised geometries (dispersion corrected ωB97X-D functional).[12]

The graphical user interface used to visualise the various properties of the intermediates was GaussView 5.0.8.

4.2 Functional Testing

The stuctures of compounds **3** and **4** were optimized using a split 6-31G**(C, H, N)/SDDAll (Al) basis set and series of different fuctionals. Key bond lengths were compared with single crystal X-ray diffraction data (SCXRD). The models all slightly over estimate the bond lengeths between the Al metal centre and heteroatoms, but in both cases the M06L functional was found to provide the best agreement between experimental and theoretical Al–Al bond lengths. The M06L functional was used to further investigate the equilibrium reactions and reported energies include a single point solvent correction using the PCM solvent model (solvent=benzene).

Table S3: Comparison of key bond lengths (Å) for compound **3** between SCXRD data and DFT optimized structures using a range of different functionals.

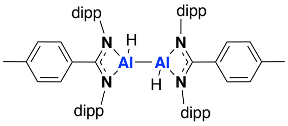

	SCXRD	wB97x	M062X	M06L
**Al1-Al1A**	2.6303	2.61279	2.61724	2.62765
**Al1-H**	1.5335	1.60907	1.60141	1.59427
**Al1-N1**	1.9443	1.98969	2.00152	2.00003
**Al1-N2**	1.9603	2.00456	2.00031	2.00234

Table S4: Comparison of key bond lengths (Å) for compound **4** between SCXRD data and DFT optimized structures using a range of different functionals.

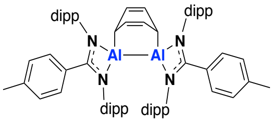

	SCXRD	wB97x	M062X	M06L	B3PW91
**Al1-Al2**	2.5419(7)	2.55923	2.55579	2.55422	2.62915
**Al1-C1**	2.017(2)	2.02669	2.03385	2.03838	2.0424
**Al2-C4**	2.018(2)	2.02669	2.03339	2.0395	2.0424
**Al1-N1**	1.948(1)	1.98817	1.98369	1.98183	2.01532
**Al1-N2**	1.943(2)	1.98428	1.98661	1.973	2.00666
**Al2-N3**	1.944(2)	1.98427	1.97843	1.97227	2.00668
**Al2-N4**	1.949(1)	1.98817	1.99383	1.98533	2.01541

4.3 Calculated energies of reactants, products and intermediates

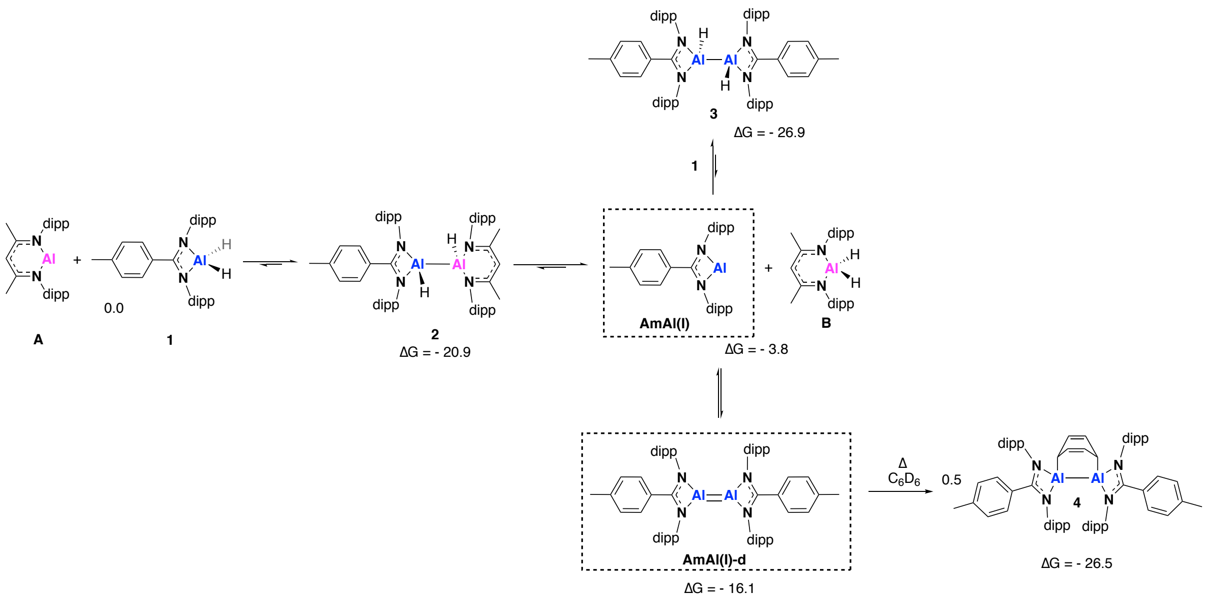

Figure S23: Proposed reaction pathway for the formation of **2-4** and **B** (M06L; Al (SDDAll), C H N (6-31G**)+ ΔE_{solv} (PCM, benzene)). Gibbs free energies relative to starting materials **A+1** (kcal mol^{-1}).

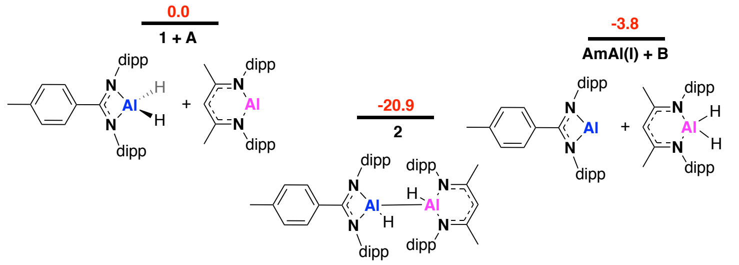

Figure S24: The relative Gibbs free energies of products from the reaction of **1** and **A** (kcal mol^{-1}).

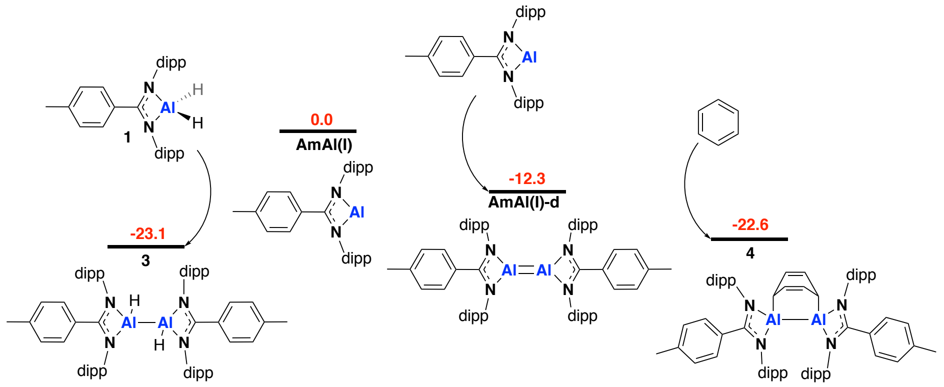

Figure S25: The relative Gibbs free energies of products for two different reaction pathways from the proposed intermediate **AmAl(I)** (kcal mol^{-1}).

Table S5: Table showing the ΔΔG (kcal mol^{-1}) between **(1+A)–(2)** and **(2)–(AmAl(I)+B)** for a series of different functionals.

	wB97xD	M062X	M06L
**ΔG (1+A)–(2)**	-20.3	-12.2	-20.9
**ΔG (2)–(AmAl(I)+B)**	19.8	12.0	17.1

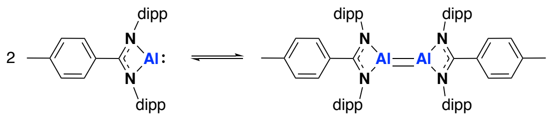

	ΔG (kcal mol-1)
**wB97xD**	-7.4
**M062X**	-2.3
**M06L**	-12.3

Figure S26: The relative Gibbs free energies for the dimerisation of AmAl(I) with a series of functionals.

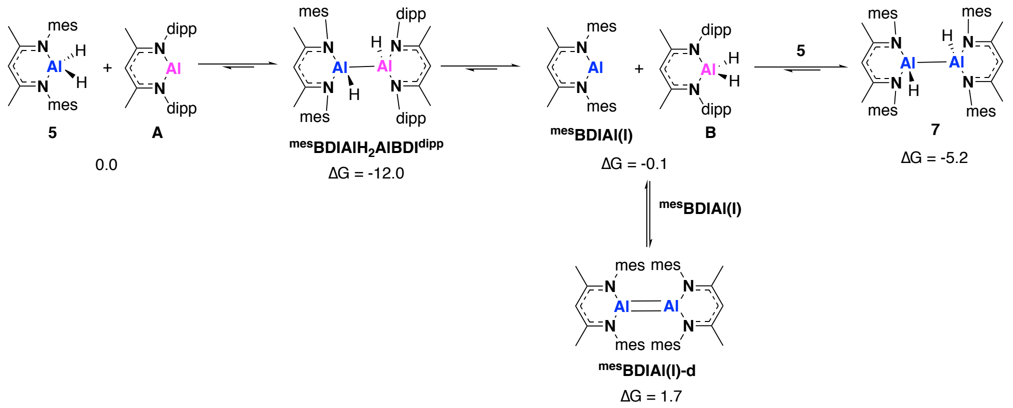

Figure S27: Proposed reaction pathway for the formation of **7** and **B**. Gibbs free energies relative to starting materials **A+5** (kcal mol-1).

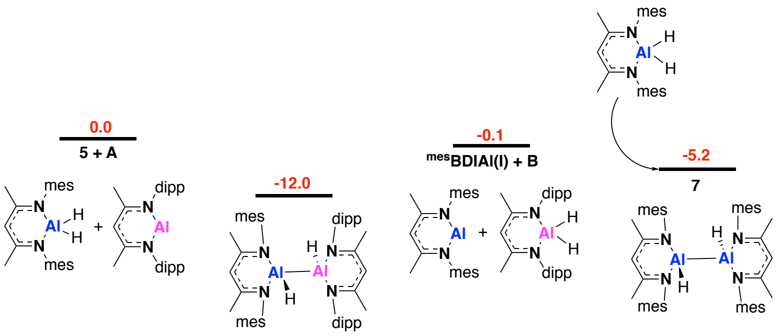

Figure S28: The relative Gibbs free energies of products from the reaction of **5** and **A** (kcal mol-1).

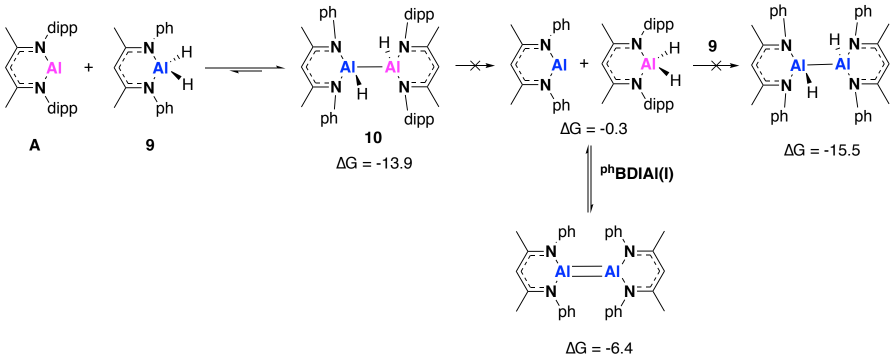

Figure S29: Proposed reaction pathway for the formation of **10**. The relative Gibbs free energies of products from the reaction of **9** and **A** (kcal mol-1).

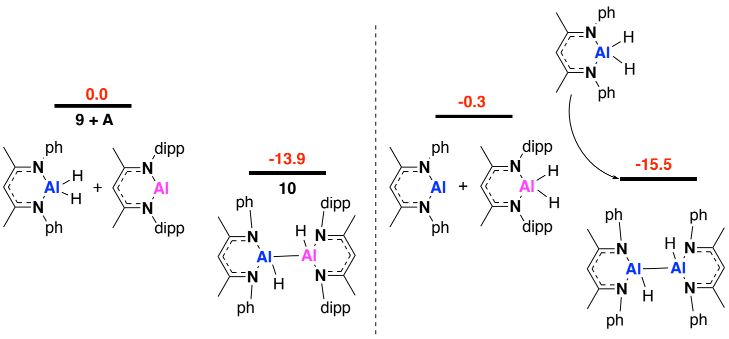

Figure S30: The relative Gibbs free energies of products from the reaction of **9** and **A** (kcal mol^{-1}).

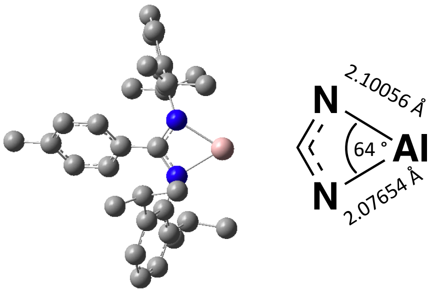

Figure S31: The optimised structure of **AmAl(I)**.

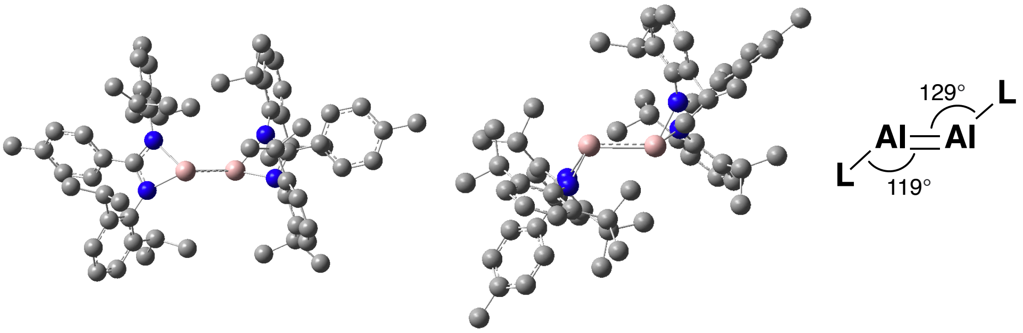

Figure S32: The optimised structure of **AmAl(I)-d** as viewed from the top and side.

6. Multinuclear NMR Data

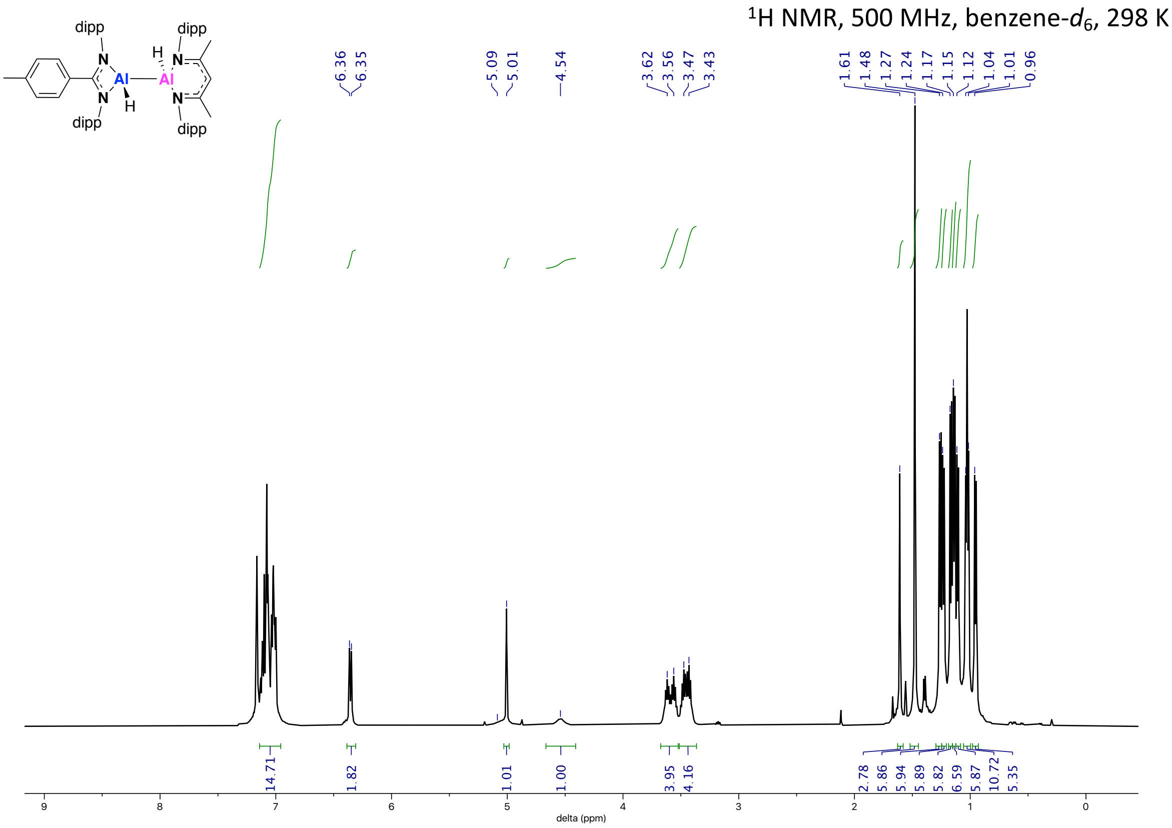

Figure S33: ^{1}H NMR spectrum of compound **2**.

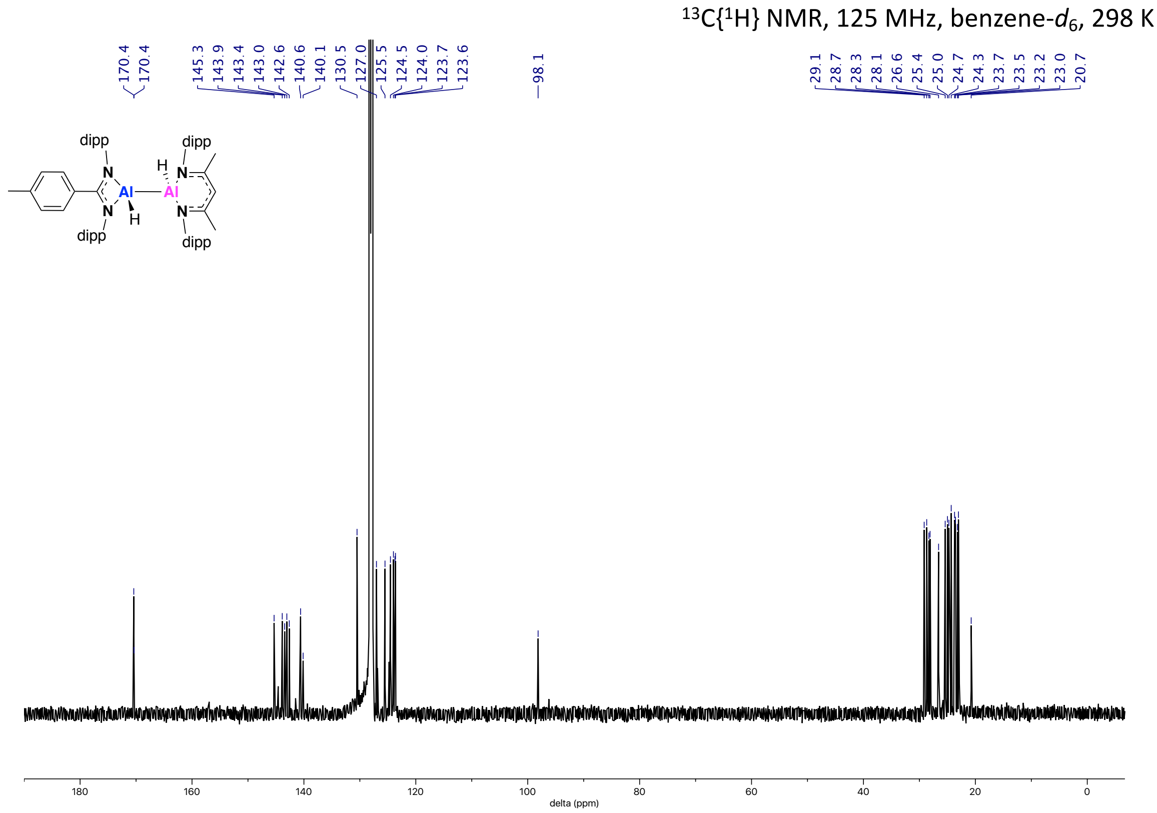

Figure S34: $^{13}C\{^{1}H\}$ NMR spectrum of compound **2**.

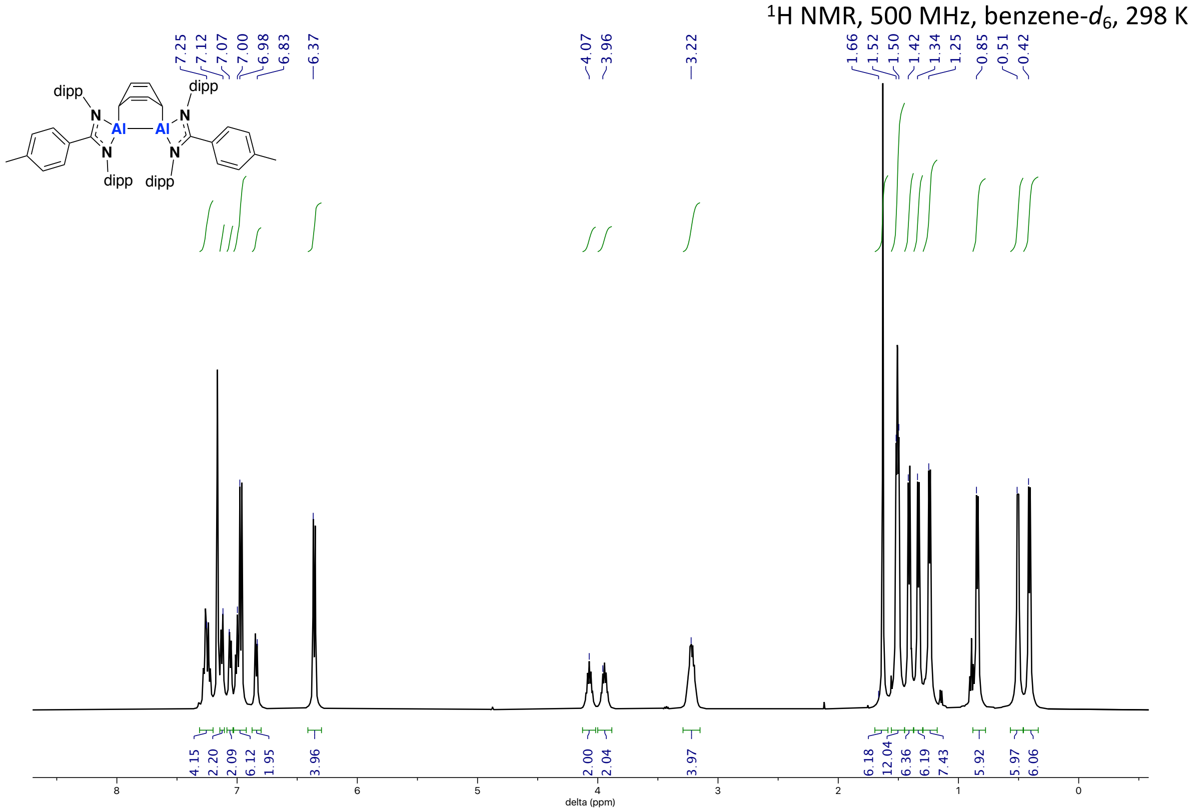

Figure S35: ^{1}H NMR spectrum of compound **4**.

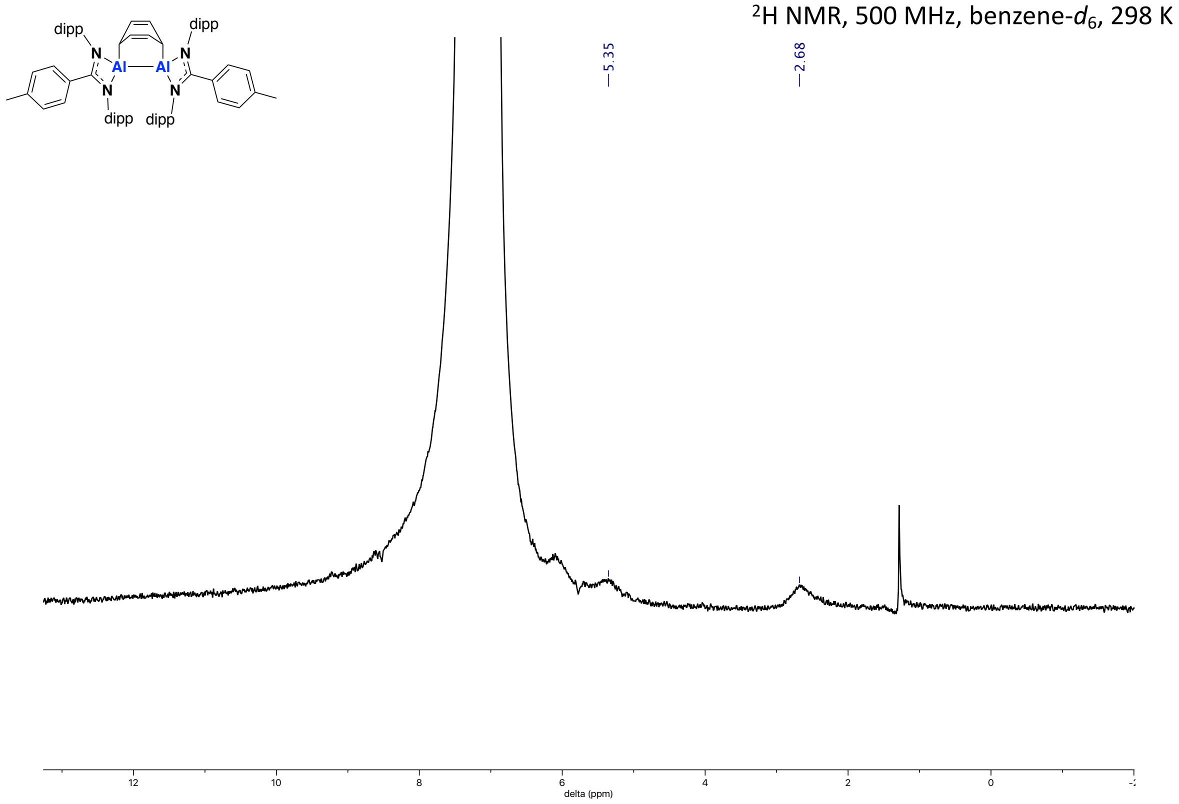

Figure S36: ^{2}H NMR spectrum of compound **4**.

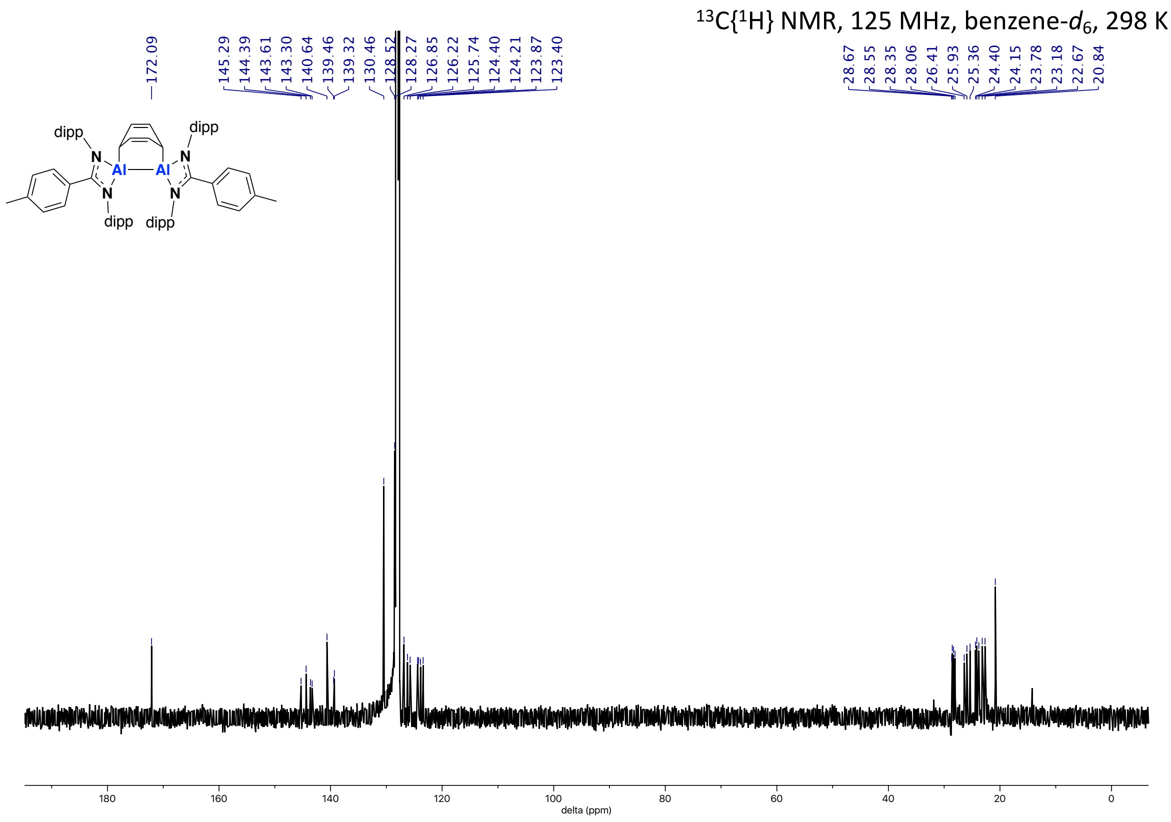

Figure S37: $^{13}C\{^{1}H\}$ NMR spectrum of compound **4**.

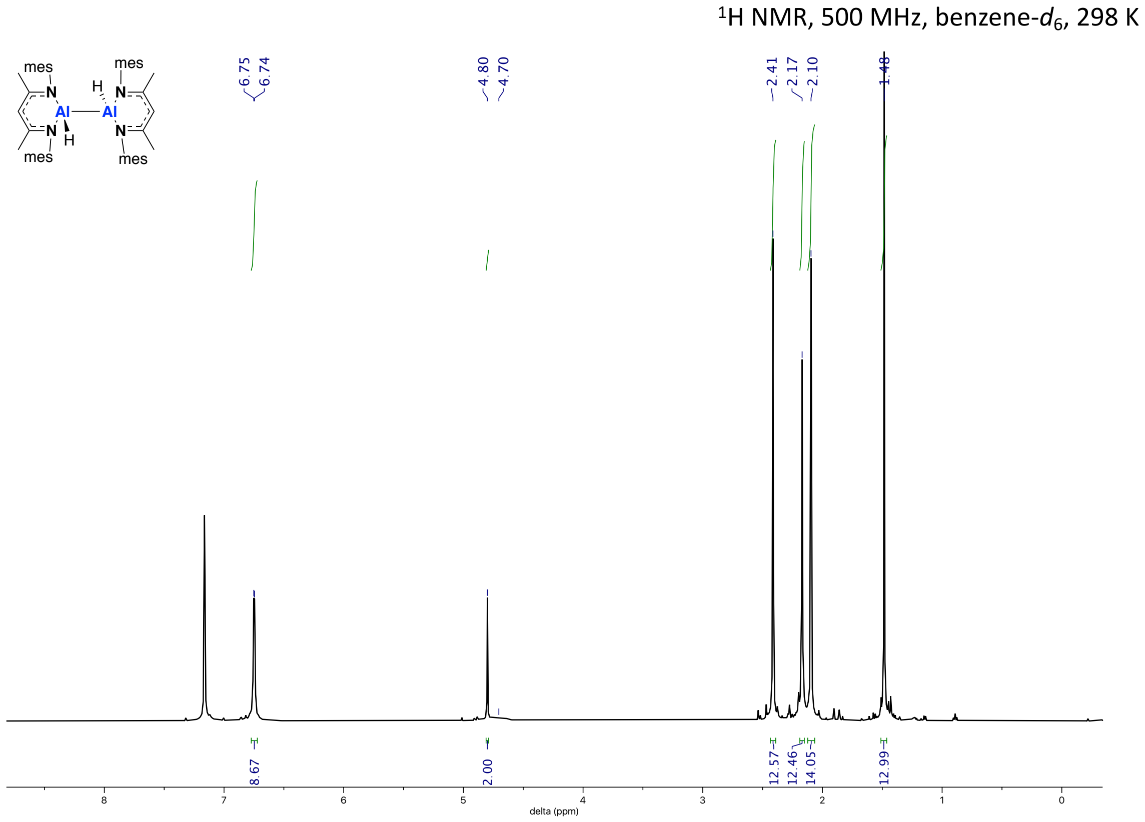

Figure S38: ^{1}H NMR spectrum of compound **7**.

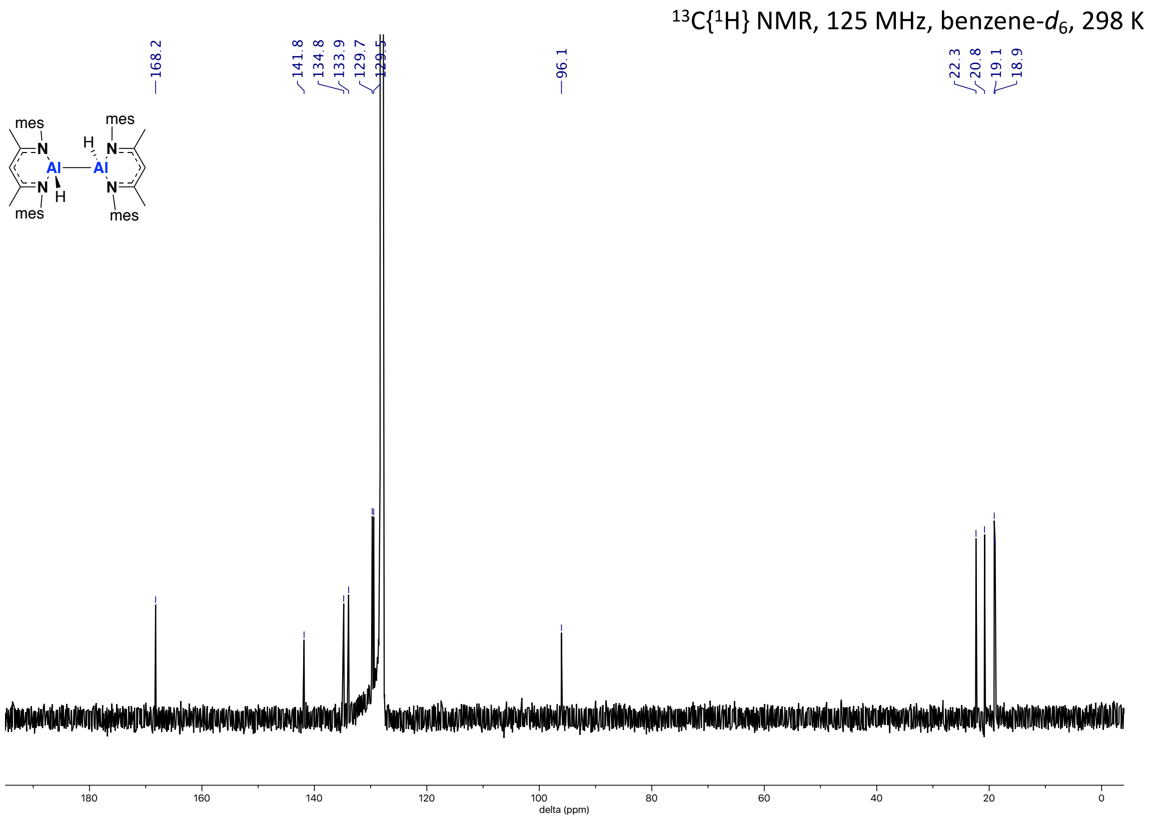

Figure S39: $^{13}C\{^{1}H\}$ NMR spectrum of compound **7**.

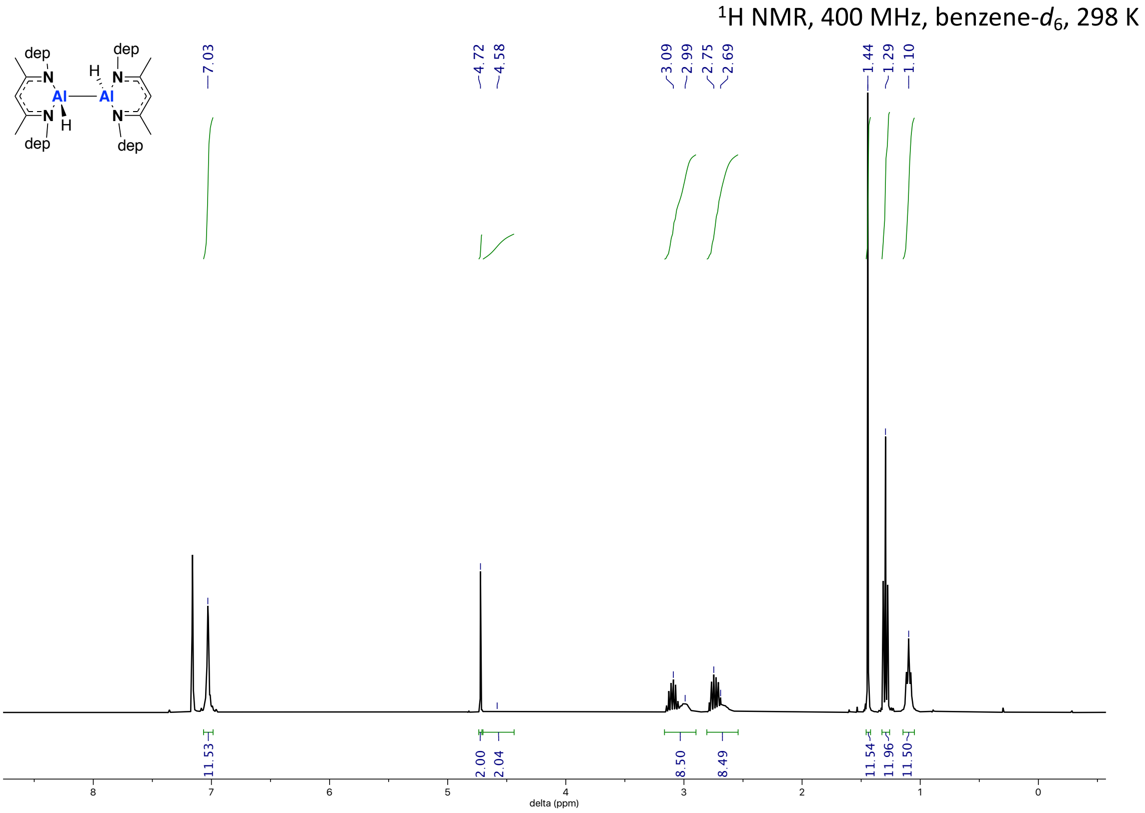

Figure S40: ^{1}H NMR spectrum of compound **8**.

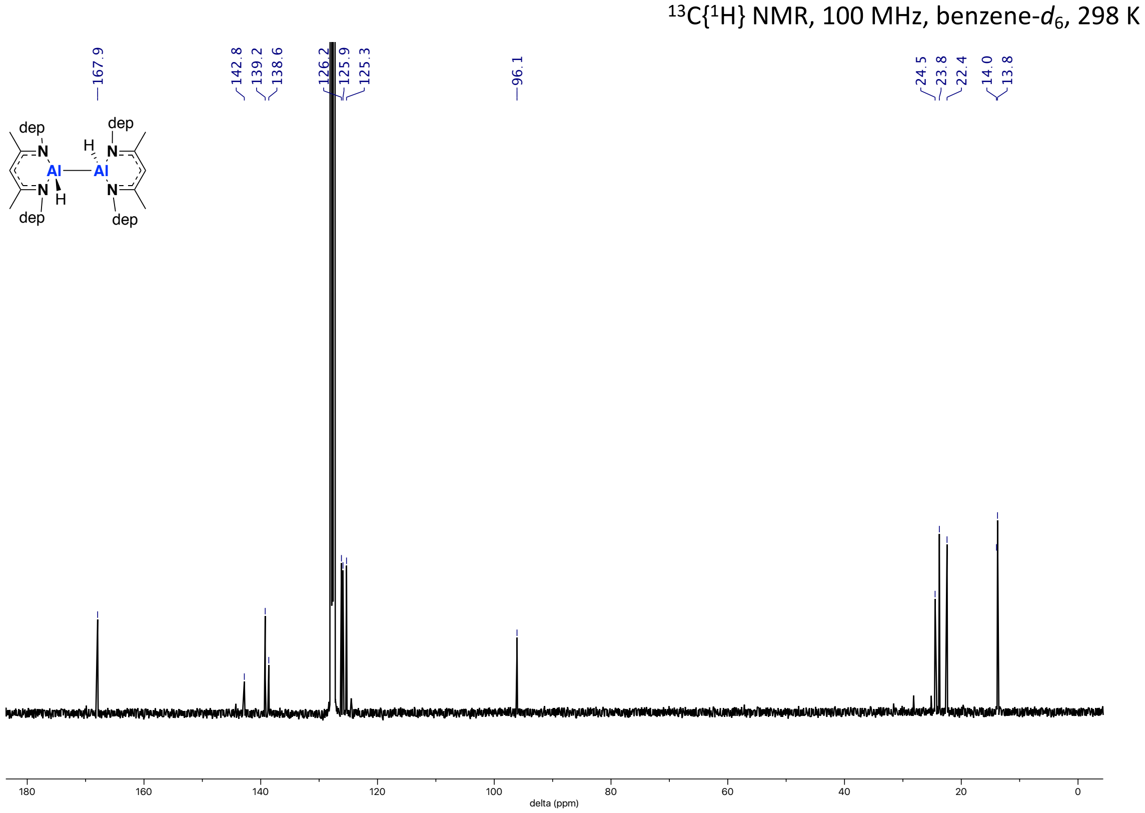

Figure S41: $^{13}C\{^{1}H\}$ NMR spectrum of compound **8**.

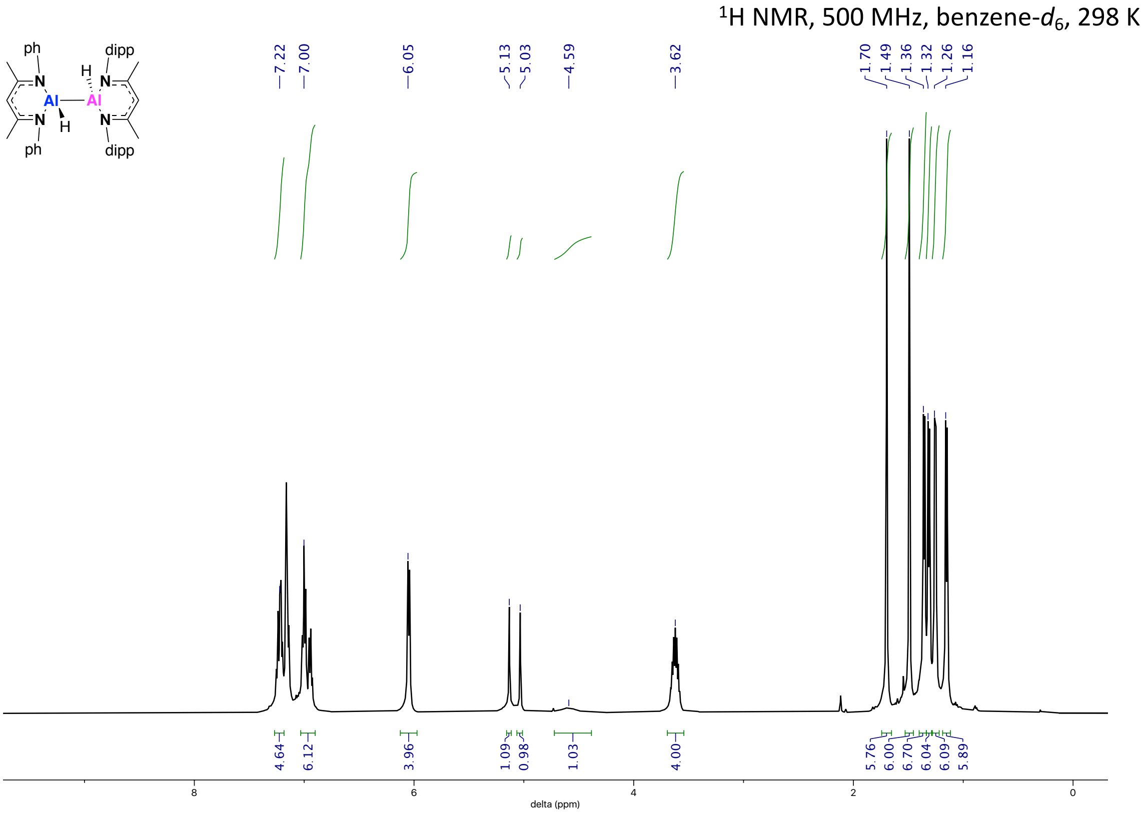

Figure S42: ^{1}H NMR spectrum of compound **10**.

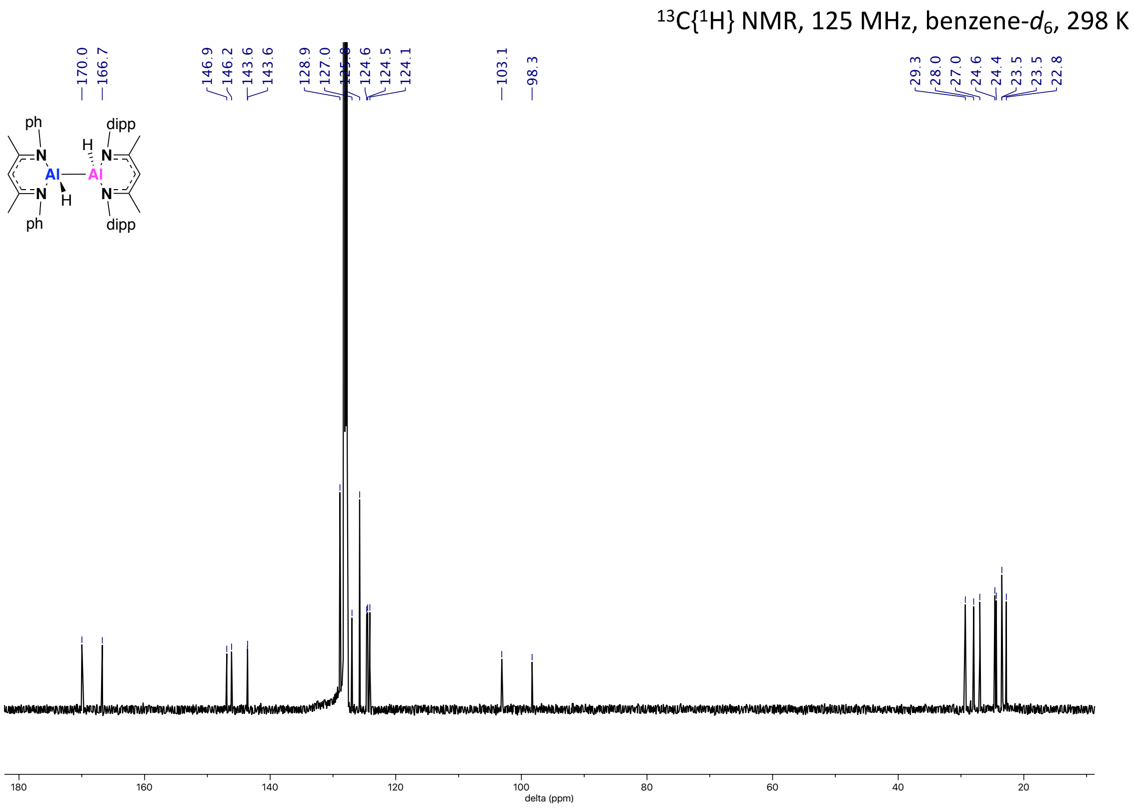

Figure S43: $^{13}C\{^{1}H\}$ NMR spectrum of compound **10**.

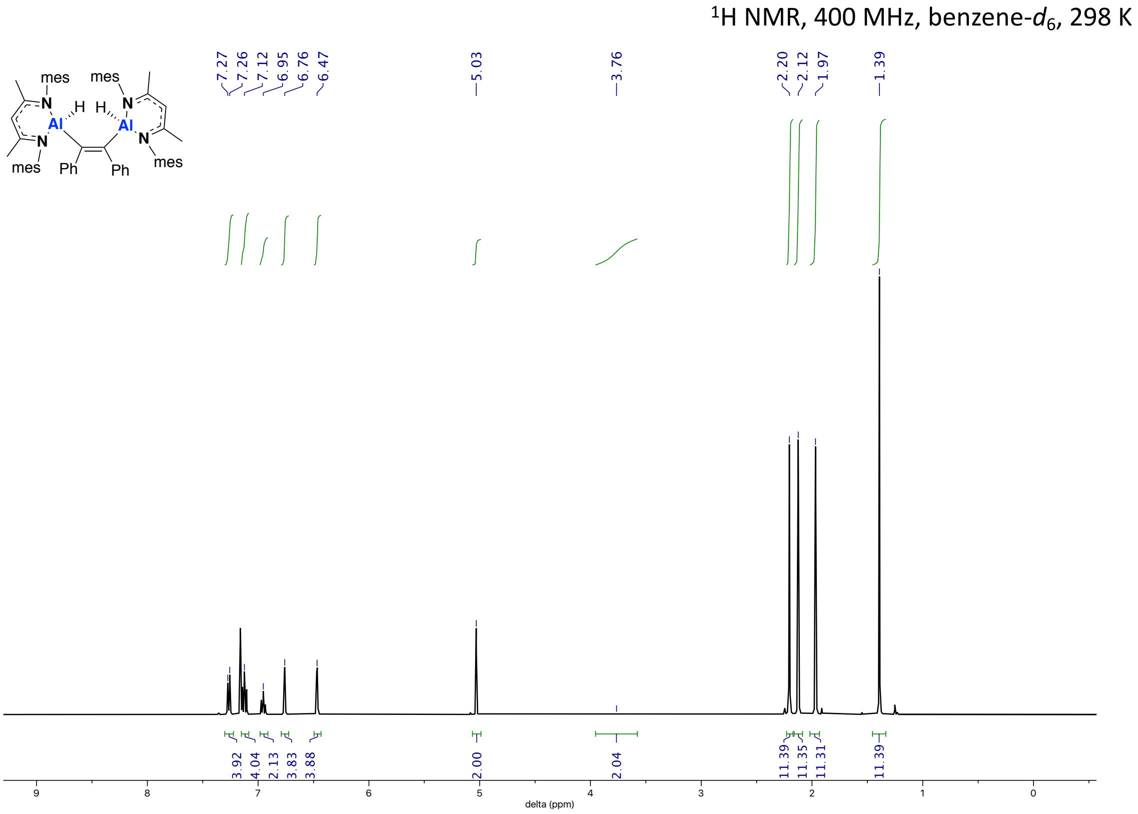

Figure S44: ^{1}H NMR spectrum of compound **11**.

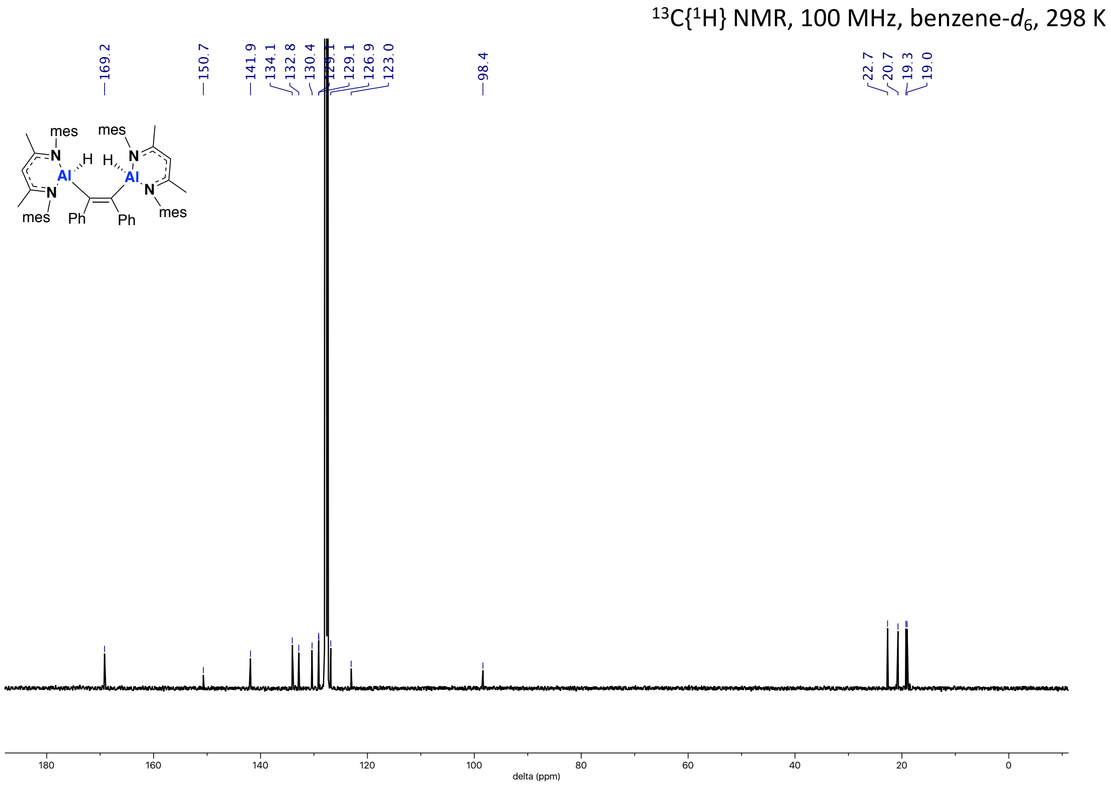

Figure S45: $^{13}C\{^{1}H\}$ NMR spectrum of compound **11**.

7. References

(1) Ruff, J. K.; Hawwthorne, M. F. *J. Am. Chem. Soc.* **2015**, No. 82, 2141.
(2) Cui, C.; Roesky, H. W.; Schmidt, H. G.; Noltemeyer, M.; Hao, H.; Cimpoesu, F. *Angew. Chem. Int. Ed.* **2000**, *39*, 4274.
(3) Hobson, K.; Carmalt, C. J.; Bakewell, C. *Inorg. Chem.* **2021**, *60*, 10958.
(4) Bonyhady, S. J.; Collis, D.; Frenking, G.; Holzmann, N.; Jones, C.; Stasch, A. *Nat. Chem.* **2010**, *2* (10), 865.
(5) Dolomanov, O. V.; Bourhis, L. J.; Gildea, R. J.; Howard, J. A. K.; Puschmann, H. *J. Appl. Cryst. 42*, 339.
(6) Sheldrick, G. M. *Act. Cryst.* **2015**, *A71*, 3.
(7) Sheldrick, G. M. *Act. Cryst.* **2015**, *C71*, 3.
(8) Hehre, W. J.; Ditchfield, R.; Pople, J. A. *J. Chem. Phys.* **1972**, *56* (5), 2257.
(9) Hariharan, P. C.; Pople, J. A. *Theoret. chim. Acta* **1973**, *28* (3), 213.
(10) Clark, T.; Chandrasekhar, J.; Spitznagel, G. W.; Rague Schleyer, von, P. *J. Comput. Chem.* **1983**, *4* (3), 294.
(11) Tomasi, J.; Mennucci, B.; Cammi, R. *Chem. Rev.* **2005**, *105* (8), 2999.
(12) Chai, J.-D.; Head-Gordon, M. *J. Chem. Phys.* **2008**, *128* (8), 84106.

8. XYZ Coordinates

1

C	4.05864100	0.11640000	-1.76397400
H	4.31977200	0.43289600	-2.77219700
C	-5.03996400	0.25216200	0.73737900
N	-1.09862400	-1.07042100	0.11097100
C	-3.03786800	-1.72661000	-1.84015600
H	-1.97475300	-1.54755000	-2.05290700
N	1.10193800	-1.06502300	0.15654700
C	-1.73593100	0.42799600	2.60506400
H	-0.74240200	0.09591900	2.28305300
C	-2.07703500	-0.35890600	3.87048100
C	-4.70538800	-0.47347300	-0.40097400
H	-5.47275200	-0.70353700	-1.13592700
C	2.73540300	-0.24452500	-1.49346800
C	3.04500200	-1.21411700	2.20674500
H	1.98108800	-0.99172200	2.36824600
C	3.40236500	-0.72080800	0.81993700
C	-4.06106300	0.54184300	1.67666000
H	-4.32493800	1.09567300	2.57583500
C	-2.73681600	0.12738000	1.50619600
C	1.73035900	-0.22485800	-2.62875400
H	0.73780700	-0.46316200	-2.23002200
C	3.83603600	-0.53475000	3.31846000
C	-1.65198900	1.92501600	2.89897500
C	4.70875900	-0.35622300	0.49831500
H	5.47792700	-0.39752700	1.26556400
C	1.64655400	1.15174500	-3.28603000
C	2.41197800	-0.65645500	-0.18507400
C	-3.39817900	-0.90493500	-0.61982300
C	2.06361100	-1.30448700	-3.65838200
C	5.03994600	0.06674600	-0.78454400
C	-2.40960600	-0.59353600	0.34005400
C	0.00176900	-0.30404500	0.04235000
C	3.21201800	-2.73283600	2.28233100
C	-3.83072700	-1.34970100	-3.08605400
C	-3.19928900	-3.21675700	-1.53389400
C	0.00114600	1.15749400	-0.13693500
C	0.96245600	1.95477500	0.49684200
C	-0.95842200	1.77399800	-0.94981100
C	0.95616800	3.33214000	0.32438800
H	1.70922700	1.49001800	1.13554700
C	-0.95087100	3.15113100	-1.12380800
H	-1.69985000	1.16550000	-1.46137300
H	1.70626200	3.93893600	0.82634700
H	-1.69457500	3.61448900	-1.76799300
C	0.00097900	3.95543800	-0.48681500
C	-0.01744800	5.44352700	-0.64820600
H	-0.64098400	5.91526000	0.12000600
H	0.98319100	5.87338100	-0.55428000
H	-0.42687700	5.74074700	-1.61714000
Al	0.00185900	-2.69986900	0.30802500
H	0.11243000	-3.58888000	-0.99429000
H	-0.10415600	-3.31257300	1.76095700
H	-3.44936400	-1.88879100	-3.95836500
H	-4.89092700	-1.60864600	-2.99113800
H	-3.76996800	-0.27744400	-3.29824900
H	-6.06250900	0.58500700	0.89242200
H	-2.63632800	-3.51390800	-0.64236800
H	-4.25184300	-3.45755700	-1.34566400
H	-2.84998400	-3.83631500	-2.36593100
H	-0.85728400	2.13539500	3.62247200
H	-1.44746100	2.50394700	1.99287000
H	-2.58926900	2.29750500	3.32746000
H	-1.34484100	-0.16402600	4.66099300
H	-3.06380200	-0.07649800	4.25476700
H	-2.09075600	-1.43581200	3.67911700
H	3.77149000	0.55632300	3.25611400
H	3.45639300	-0.84013400	4.29807000
H	4.89718100	-0.80545800	3.29070000
H	2.64512300	-3.24509600	1.49764600
H	4.26480300	-3.00969400	2.15483600
H	2.86977200	-3.12505200	3.24529900
H	0.84927400	1.17624800	-4.03637200
H	1.44519300	1.93812900	-2.55181900
H	2.58222200	1.40493400	-3.79711800
H	1.32863100	-1.31006200	-4.47006700
H	3.04976600	-1.13152900	-4.10417900
H	2.07334700	-2.29918100	-3.20344800
H	6.06179400	0.35163300	-1.01928200

2

C	-3.49880800	-4.59394100	0.03332900
H	-4.33527800	-5.10516300	0.50683400
C	-3.20273700	4.86672000	0.25599900
N	-2.15804200	0.81930300	0.63815600
C	-3.60990700	2.03132700	2.78312500
H	-3.66912400	0.97159500	2.49652300
N	-1.80580000	-1.28621100	0.09756300
C	-1.69727900	2.14970700	-1.94125400
H	-1.53957100	1.09705600	-1.68597300
C	-0.32591800	2.72608200	-2.27440700
C	-3.51096300	4.16531400	1.41764600
H	-3.99165100	4.68289100	2.24491800
C	-3.22955500	-3.26933100	0.39028300
C	-0.09991100	-2.61419000	-1.73898900
H	0.37744200	-2.05425200	-0.91902200
C	-1.31273500	-3.30504500	-1.14557300
C	-2.62649800	4.19886100	-0.81712400
H	-2.40345000	4.73964500	-1.73575100
C	-2.31804100	2.83806800	-0.74417000
C	-4.07924400	-2.62234900	1.46726000
H	-3.79281700	-1.56964700	1.56222300
C	-0.47298800	-1.58621600	-2.80682100
C	-2.62460600	2.19562800	-3.15443500
C	-1.62355000	-4.62587900	-1.46541500
H	-0.99532300	-5.16506700	-2.16877200
C	-5.56733900	-2.67085900	1.12477100
C	-2.14388600	-2.61678100	-0.22963200
C	-3.22356300	2.80528500	1.53667800
C	-3.81029600	-3.27356700	2.82356100
C	-2.71720200	-5.26814900	-0.89270300
C	-2.59114900	2.15224200	0.45646000
C	-2.63402800	-0.24216300	-0.03130300
C	0.94389000	-3.57721900	-2.28274200
C	-4.97480400	2.44487600	3.32712900
C	-2.54703800	2.13226100	3.87511000
C	-3.90029800	-0.24541600	-0.79157300
C	-4.04251700	-0.98784600	-1.97178600
C	-5.00095200	0.48752800	-0.32889900
C	-5.24119400	-0.97506300	-2.67221400
H	-3.20916100	-1.57637700	-2.34314900
C	-6.19951000	0.48527300	-1.02922600

H	-4.92179300	1.04295400	0.60054900
H	-5.32986200	-1.55358900	-3.58906000
H	-7.04529500	1.05136100	-0.64499000
C	-6.34028200	-0.23738100	-2.21826700
C	-7.62111600	-0.20879300	-2.99275600
H	-7.60104100	0.57330100	-3.76057300
H	-7.80309600	-1.15524800	-3.50908100
H	-8.47950500	-0.00169000	-2.34845800
Al	-0.53387400	-0.20561800	1.25504000
H	-0.82195900	-0.59780300	2.78061900
H	-5.28337800	1.77408200	4.13483500
H	-4.95913700	3.45840300	3.74288000
H	-5.74900800	2.42082200	2.55268900
H	-3.42834200	5.92730400	0.18303300
H	-1.58930400	1.72476300	3.53959800
H	-2.39205900	3.17696900	4.17169200
H	-2.84861300	1.57076500	4.76563200
H	-2.20900900	1.60755900	-3.98056500
H	-3.61666400	1.79668200	-2.92017200
H	-2.75458800	3.22326100	-3.51369800
H	0.14820000	2.16123800	-3.08541500
H	-0.39292900	3.77392300	-2.58936400
H	0.34256600	2.69135600	-1.40776600
H	-1.14995300	-0.82049200	-2.41719300
H	0.42363800	-1.07345800	-3.17336800
H	-0.96260000	-2.07262200	-3.65985500
H	1.21440300	-4.34135600	-1.54742500
H	0.59777000	-4.08445700	-3.19127600
H	1.85642200	-3.03127600	-2.54229600
H	-6.15238700	-2.09965000	1.85361300
H	-5.76758500	-2.25740100	0.13136500
H	-5.94491100	-3.69968000	1.13918800
H	-4.41200100	-2.80404100	3.60905000
H	-4.05912700	-4.34096500	2.80525500
H	-2.75707000	-3.18054600	3.10498700
N	3.05045600	1.70386600	0.50820400
C	4.02215400	1.74290100	1.42803600
C	4.43702500	0.62511600	2.16821100
H	5.19513700	0.81001000	2.92027200
C	4.14403600	-0.71906000	1.90840800
N	3.22686000	-1.11234300	1.01680200
C	4.76587800	3.02525300	1.68021100
H	5.03159000	3.52079900	0.74216800
H	5.67520900	2.84020200	2.25306000
H	4.15300800	3.73859000	2.24039400
C	4.95863500	-1.75090900	2.63389300
H	4.30892300	-2.43402700	3.19078400
H	5.65715200	-1.28552700	3.32988000
H	5.52418000	-2.37646200	1.93582800
C	2.76276400	2.91018500	-0.21863700
C	3.18251300	3.02099400	-1.56305900
C	2.86144900	4.18740000	-2.26114400
H	3.17510200	4.28984000	-3.29589700
C	2.14083800	5.21427900	-1.66477100
C	1.73414100	5.08949800	-0.34533000
H	1.15597700	5.88563800	0.11860100
C	2.04359700	3.95144300	0.40463200
C	3.99332800	1.92610000	-2.22973700
H	3.61986000	0.96914100	-1.84785300
C	1.54424700	3.86454900	1.83351300
H	2.05282700	3.03213800	2.33389900
C	3.31001000	-2.46981100	0.55483300
C	2.64163800	-3.51271200	1.22297200
C	2.81363700	-4.81899700	0.75565500
H	2.29853100	-5.63170100	1.26403200
C	3.62996900	-5.09567500	-0.33288100

C	4.27407600	-4.05404300	-0.98794700
H	4.91362500	-4.26674600	-1.84282600
C	4.12569800	-2.73127300	-0.56606600
C	1.73352100	-3.26468900	2.40960300
H	1.84176900	-2.21282000	2.70736500
C	4.88675300	-1.63285500	-1.28379700
H	4.57760800	-0.67035800	-0.85938000
H	1.89264000	6.10735800	-2.23198600
H	3.75897800	-6.11941700	-0.67338200
C	4.56151500	-1.59844400	-2.77417800
H	5.15867800	-0.82924500	-3.27702700
H	4.78361300	-2.55360700	-3.26359500
H	3.50373400	-1.36640500	-2.94121700
C	6.39227200	-1.77270100	-1.05726200
H	6.64942700	-1.72552100	0.00610300
H	6.76581000	-2.72728500	-1.44563500
H	6.93902500	-0.97127900	-1.56561900
C	0.28000000	-3.48359800	1.99976200
H	0.09974200	-4.52183400	1.69365800
H	-0.40828500	-3.23959000	2.81464800
H	0.02045500	-2.85994200	1.13890800
C	2.08106200	-4.13947800	3.61239200
H	1.90967300	-5.19995100	3.39746800
H	3.12773000	-4.03570100	3.91641700
H	1.45434000	-3.87767600	4.47018400
C	1.82844100	5.13551600	2.63263000
H	2.88090900	5.43417000	2.58157000
H	1.23424100	5.98063600	2.26962000
H	1.56837500	4.99082100	3.68559300
C	0.05293900	3.55172100	1.84956600
H	-0.32931900	3.49786700	2.87337600
H	-0.52852000	4.31023400	1.31178300
H	-0.14688200	2.59248300	1.35968200
C	5.47396500	2.01811600	-1.85712800
H	5.89445100	2.99220100	-2.13420300
H	5.63724200	1.87032200	-0.78425300
H	6.04958000	1.24674800	-2.38259000
C	3.82328500	1.89374400	-3.74375900
H	4.26878200	2.76751300	-4.23240700
H	4.31834100	1.01195900	-4.16040400
H	2.76704200	1.84899000	-4.02842800
Al	1.90561700	0.10333700	0.19903100
H	1.94366100	-0.18860500	-1.37614700
H	-2.94598800	-6.29710800	-1.15679800

3

Al	-1.07309300	0.36596900	-1.22318000
N	-2.68100900	1.26890600	-0.44895200
C	-3.26435300	0.08429800	-0.19729200
H	-0.98197200	0.56161600	-2.80276900
N	-2.53032100	-0.91120000	-0.71850900
C	-4.49554900	-0.08791500	0.59821800
C	-4.73948100	0.75376500	1.69266900
H	-4.01888300	1.52750300	1.94391100
C	-5.87727400	0.58980300	2.46998800
H	-6.04176800	1.24452700	3.32281900
C	-6.81551400	-0.40714900	2.17878300
C	-6.56533900	-1.24635600	1.08840200
H	-7.27650300	-2.03327400	0.84761200
C	-5.42361700	-1.09744800	0.31162600
H	-5.25331100	-1.76938700	-0.52416200
C	-8.05912300	-0.55583900	2.99874600
H	-7.87985500	-0.32249800	4.05186500
H	-8.46497000	-1.56887800	2.93868200

H	-8.84431200	0.12612500	2.65242800
C	-3.32199100	2.52247500	-0.33617400
C	-2.72958100	3.50737400	0.48659100
C	-3.34209500	4.75394300	0.59864500
H	-2.89296600	5.51077800	1.23734400
C	-4.51828000	5.03943500	-0.08638500
H	-4.98836000	6.01375000	0.01454000
C	-5.07938400	4.07325300	-0.90911100
H	-5.98699000	4.30055100	-1.46563200
C	-4.50035000	2.80994800	-1.05737100
C	-1.42225800	3.21030300	1.18327500
H	-1.39115500	2.12740900	1.37013400
C	-0.25721900	3.55472300	0.25310800
H	-0.37702800	3.11513800	-0.74507800
H	0.69930000	3.21087800	0.66263100
H	-0.17775600	4.63950400	0.11086300
C	-1.25029900	3.90416400	2.52789600
H	-1.14315600	4.98996600	2.42231900
H	-0.34580200	3.53871300	3.02536000
H	-2.09963500	3.71647000	3.19240600
C	-5.12270600	1.83320500	-2.03362400
H	-4.60413100	0.87482100	-1.94253100
C	-6.60111100	1.58466600	-1.74525900
H	-6.76398000	1.28495500	-0.70520600
H	-6.99169200	0.79054900	-2.39159700
H	-7.20227200	2.48161400	-1.93265400
C	-4.91216600	2.30466400	-3.47206500
H	-5.38681400	3.27789900	-3.64232200
H	-5.34663100	1.59338900	-4.18309700
H	-3.84732200	2.40712800	-3.70288900
C	-2.73490800	-2.28622100	-0.46496000
C	-2.59170200	-2.82547300	0.82795700
C	-2.81765800	-4.19377000	1.00401000
H	-2.71143400	-4.62030900	1.99980100
C	-3.15278700	-5.01460300	-0.06309000
H	-3.32917600	-6.07484100	0.09660400
C	-3.23162300	-4.47818000	-1.34479300
H	-3.45665700	-5.13020000	-2.18462000
C	-3.01366400	-3.11982200	-1.57195600
C	-2.12037700	-2.00417400	2.01140700
H	-2.06270900	-0.94868800	1.71435900
C	-3.07860100	-2.09790400	3.19665600
H	-4.09966100	-1.82530500	2.91118000
H	-2.76180100	-1.43032200	4.00447600
H	-3.10692300	-3.11417100	3.60597300
C	-0.70771900	-2.43293300	2.41318600
H	-0.68002700	-3.48433300	2.72336400
H	-0.33532000	-1.82261900	3.24218700
H	0.00082800	-2.33295300	1.58086700
C	-3.07144000	-2.53665300	-2.97101700
H	-2.31532600	-1.74044500	-3.01903500
C	-4.42398200	-1.87939000	-3.24782800
H	-5.23339100	-2.61801400	-3.19745000
H	-4.44466100	-1.42622600	-4.24473400
H	-4.64351900	-1.09019000	-2.52257300
C	-2.74540500	-3.55066800	-4.06091700
H	-1.80940100	-4.08190300	-3.85800800
H	-2.64715100	-3.04905300	-5.02811800
H	-3.53566400	-4.30206300	-4.17052600
Al	0.87786700	0.32711700	0.53658800
N	2.35700900	-0.98296100	0.28450200
C	3.24423700	-0.00173900	0.04065900
H	0.58366500	0.54192100	2.09678500
N	2.63704600	1.19177800	0.12161900
C	4.66128600	-0.22657200	-0.30236400
C	5.03301000	-1.39198900	-0.98938500
H	4.27299200	-2.11444700	-1.26720200
C	6.35614800	-1.62269000	-1.33369300
H	6.61785600	-2.53160600	-1.87119900
C	7.36051700	-0.70612300	-1.00162600
C	6.98689600	0.45550800	-0.31972300
H	7.74926000	1.18323300	-0.05126300
C	5.66193700	0.70119600	0.01827700
H	5.40788200	1.61826600	0.53881100
C	8.78513000	-0.95605100	-1.38710300
H	8.93406600	-0.81991500	-2.46413300
H	9.46851300	-0.27469200	-0.87481200
H	9.09141700	-1.98059700	-1.15561400
C	2.66399500	-2.28986300	0.73171000
C	2.34143500	-3.38377600	-0.10101600
C	2.51819100	-4.67623300	0.39114600
H	2.26697900	-5.52564800	-0.23943800
C	3.01814600	-4.89451300	1.67052100
H	3.14985200	-5.90795800	2.03958400
C	3.35675000	-3.81083000	2.46869900
H	3.75968200	-3.98016400	3.46589400
C	3.18607600	-2.49675300	2.02455900
C	1.79475700	-3.14382200	-1.49333700
H	2.16049100	-2.15901700	-1.81986200
C	0.27195300	-3.06337500	-1.45767600
H	-0.06333000	-2.30369500	-0.74326700
H	-0.14128100	-2.80625000	-2.44114200
H	-0.17312200	-4.01503500	-1.14181200
C	2.25632800	-4.17771000	-2.51406800
H	1.83179000	-5.16720200	-2.31267800
H	1.93221900	-3.89179500	-3.51945800
H	3.34637100	-4.28290500	-2.52689900
C	3.56748900	-1.35158900	2.94050200
H	3.28958300	-0.41107300	2.45168000
C	5.07658200	-1.32854300	3.18588400
H	5.63916600	-1.28331800	2.24745400
H	5.36091300	-0.46281300	3.79450000
H	5.39931800	-2.22889300	3.72166500
C	2.80299500	-1.39975400	4.26110000
H	3.03111700	-2.31078200	4.82599800
H	3.07054100	-0.54596500	4.89277400
H	1.72220600	-1.36933900	4.09512700
C	3.16988800	2.41657300	-0.33377400
C	3.47917500	2.62008800	-1.69323400
C	4.00055500	3.85963300	-2.07482100
H	4.24554700	4.02654200	-3.12233000
C	4.19307400	4.87808400	-1.15306600
H	4.59889700	5.83441400	-1.47137600
C	3.84419900	4.67647800	0.17890200
H	3.97617900	5.48471800	0.89314800
C	3.32776600	3.45605100	0.61295800
C	3.22108000	1.57449000	-2.76062000
H	2.85390300	0.66052700	-2.27839100
C	4.49234700	1.21712700	-3.52921100
H	5.29796400	0.90268300	-2.85836800
H	4.30209400	0.40320000	-4.23662900
H	4.85720900	2.07336600	-4.10814200
C	2.12470500	2.04953700	-3.71427700
H	2.43823700	2.94971900	-4.25538900
H	1.88816000	1.27935600	-4.45560600
H	1.20124000	2.28853800	-3.17808300
C	2.96914200	3.22868600	2.07157300
H	2.09548200	2.56178200	2.09814000
C	4.08784500	2.49796600	2.81519800
H	5.01314900	3.08706700	2.80950200
H	3.81020200	2.31950100	3.86004800
H	4.30383700	1.52547900	2.36309600

C	2.59377900	4.51000200	2.80583300
H	1.82785500	5.07853500	2.26771800
H	2.20341500	4.27663200	3.80117800
H	3.45817900	5.16813400	2.94924500

4

Al	-1.27828400	0.14987100	-1.37588500
Al	1.25788100	-0.15233400	-1.35151500
N	-2.44780000	1.21118900	-0.17860400
N	-2.72724500	-0.93496300	-0.59079400
N	2.43494900	-1.22124200	-0.16260900
N	2.69388400	0.93317600	-0.54565200
C	-3.16415800	0.11595600	0.13038600
C	-4.23121400	0.04542100	1.14005700
C	-2.49794300	2.44848800	0.50070800
C	-2.14826800	2.57372200	1.86031900
C	2.50234500	-2.46895300	0.49610300
C	3.14638200	-0.12494500	0.15557400
C	-1.59566900	1.42943900	2.68882700
H	-1.59990200	0.51674800	2.07903600
C	-3.47512100	-2.11363800	-0.82831600
C	3.44369600	2.10666300	-0.80604900
C	-6.22790100	-0.13063500	3.12713400
C	-5.11198200	1.11239300	1.36287900
H	-5.02875900	2.01755300	0.76730900
C	-6.09625500	1.01742000	2.33823300
H	-6.77579600	1.85201200	2.49524400
C	2.88834500	3.36284900	-0.48192700
C	-2.86916000	3.58680400	-0.25520400
C	-0.14333700	1.71691000	3.07916200
H	0.51234900	1.75453600	2.20209300
H	0.24480500	0.94729700	3.75425300
H	-0.05818000	2.67882600	3.59744600
C	2.88287600	-3.59126600	-0.27965200
C	4.22052700	-0.05921300	1.15766600
C	-2.97908900	4.81611600	0.39246500
H	-3.27219300	5.69370500	-0.17761100
C	1.41076000	-0.27983800	-3.38127600
H	2.42185800	-0.50784000	-3.73905300
C	-5.33512200	-1.18678900	2.90892800
H	-5.40944100	-2.08274400	3.52109700
C	2.16375000	-2.61994700	1.85594200
C	-4.35617300	-1.10666300	1.92942100
H	-3.66367400	-1.93161300	1.78062300
C	-4.75979600	-2.05303800	-1.41469400
C	5.10002500	-1.12791500	1.37964200
H	5.01421800	-2.03426900	0.78678200
C	-2.89278000	-3.36373100	-0.52678700
C	4.70314400	2.03508400	-1.44541300
C	3.00439500	-4.83045300	0.34678100
H	3.30283800	-5.69582300	-0.23902300
C	1.49702500	3.50075400	0.09570400
H	1.14588200	2.49791000	0.37203000
C	3.62543000	4.51743900	-0.74967600
H	3.19446200	5.48462700	-0.49866700
C	4.35139300	1.09314600	1.94595100
H	3.66053000	1.91931600	1.79794200
C	-2.43850000	1.16816400	3.93694300
H	-2.37158300	2.00668600	4.63972500
H	-2.08419300	0.27427500	4.46260800
H	-3.49505600	1.02042600	3.69461300
C	-2.27375400	3.83018100	2.46308800
H	-2.01479100	3.93424600	3.51543200
C	-2.70280900	4.93993100	1.75038300
H	-2.79792400	5.90386200	2.24248800
C	1.59538400	-1.49749300	2.70278200
H	1.58123300	-0.57681500	2.10570500
C	0.97067300	1.11156700	-3.72798100
H	1.72438000	1.86404800	-3.95627200
C	6.08797700	-1.03345400	2.35120100
H	6.76610800	-1.86946000	2.50678500
C	-1.37655800	0.39709000	-3.39683100
H	-2.37179300	0.66597200	-3.77043600
C	-0.34133800	1.42816100	-3.72821100
H	-0.66496800	2.43964600	-3.97181700
C	2.31179100	-3.88303000	2.43944100
H	2.06421400	-4.00561700	3.49248300
C	6.22514600	0.11515200	3.13833700
C	4.88614500	4.45542000	-1.32709100
H	5.44943100	5.36450400	-1.51870800
C	-1.47396400	-3.47069600	-0.01440600
H	-1.16977800	-2.47570000	0.33790500
C	0.39110100	-1.26539400	-3.86245700
H	0.72699900	-2.24495700	-4.20039000
C	5.40700000	3.21838000	-1.67867800
H	6.37869500	3.16064900	-2.16621000
C	5.33379100	1.17277600	2.92182900
H	5.41193100	2.06952700	3.53239400
C	-5.38301800	-0.76096300	-1.89966600
H	-4.74103600	0.06937200	-1.59297100
C	-0.91875900	-0.94841800	-3.87735200
H	-1.66281500	-1.66741600	-4.21650600
C	2.74333200	-4.97769800	1.70538500
H	2.85229400	-5.94820000	2.18144100
C	5.29432200	0.73958100	-1.96062200
H	4.62969800	-0.08118900	-1.67498900
C	2.43664800	-1.23700500	3.95187000
H	2.39273400	-2.08782800	4.64155000
H	2.06347300	-0.36037000	4.49327000
H	3.48832100	-1.06002000	3.70775600
C	-5.45676700	-3.24434900	-1.62775800
H	-6.44690200	-3.19723100	-2.07774500
C	1.45790500	4.37462600	1.34658800
H	2.12548400	3.99929200	2.13017100
H	0.44149300	4.40807500	1.75483900
H	1.75474400	5.40634600	1.12752900
C	-3.13762200	3.45986900	-1.74223500
H	-2.45852700	2.68524900	-2.12448200
C	0.14929800	-1.81832800	3.09124000
H	-0.50502900	-1.86186700	2.21355100
H	-0.25531600	-1.06325700	3.77330300
H	0.08432700	-2.78702200	3.59991400
C	-7.30556600	-0.23652600	4.16072100
H	-7.61869000	0.74647500	4.52174200
H	-6.98390100	-0.82910500	5.02144200
H	-8.19679200	-0.72748200	3.75300600
C	3.16528200	-3.43500800	-1.76170800
H	2.49951600	-2.64494600	-2.13585400
C	-3.62549400	-4.52616500	-0.76969400
H	-3.17662200	-5.48934200	-0.53707300
C	0.54513700	4.04432700	-0.96846400
H	0.90394700	5.00491900	-1.35783300
H	-0.45157000	4.20492300	-0.54763600
H	0.45635500	3.35357200	-1.81614900
C	-6.76977900	-0.52206700	-1.30671000
H	-7.49276500	-1.25676800	-1.67881000
H	-7.14370400	0.47019200	-1.58367500
H	-6.76047800	-0.58809900	-0.21453100
C	-4.90739500	-4.47586500	-1.30116600
H	-5.46655200	-5.39097400	-1.47552700
C	6.67605900	0.45783900	-1.37316600

H	7.41140000	1.18802500	-1.72993400
H	7.03147200	-0.53448500	-1.67363600
H	6.67059000	0.49990400	-0.27994100
C	-5.43344800	-0.73378200	-3.42741500
H	-4.43753200	-0.86288000	-3.86302000
H	-5.84224700	0.21640000	-3.78852100
H	-6.06962500	-1.53798000	-3.81430800
C	5.35236200	0.74645900	-3.48854800
H	4.36522600	0.92028300	-3.92804100
H	5.73076600	-0.20861600	-3.86924900
H	6.01942600	1.53559900	-3.85371300
C	-1.31985400	-4.44523200	1.14889500
H	-1.99225800	-4.20092400	1.97866500
H	-0.29186800	-4.42226800	1.52751900
H	-1.52894300	-5.47727400	0.84641500
C	-2.85464700	4.73439100	-2.52791000
H	-3.57632900	5.52698000	-2.30025100
H	-2.92861700	4.53661800	-3.60181500
H	-1.85228000	5.12506200	-2.32620400
C	-0.54220900	-3.85268500	-1.16371600
H	-0.84130500	-4.81309000	-1.60143700
H	0.48702000	-3.95284700	-0.80653200
H	-0.55902400	-3.10414700	-1.96566000
C	7.30656900	0.21984000	4.16803000
H	6.98970600	0.81537000	5.02849500
H	8.19795700	0.70704600	3.75618600
H	7.61782000	-0.76335900	4.53014500
C	-4.56580500	2.98229500	-2.00273400
H	-4.78077900	2.05147200	-1.47015700
H	-4.73564100	2.80332300	-3.07028900
H	-5.29135500	3.73358100	-1.66803300
C	2.88064600	-4.69021800	-2.57733900
H	3.58929100	-5.49549900	-2.35344600
H	2.97523200	-4.47278300	-3.64569300
H	1.87075800	-5.07316000	-2.40017000
C	4.60184800	-2.96712800	-1.99309700
H	4.82456300	-2.05844000	-1.42660600
H	4.78363800	-2.75365700	-3.05248900
H	5.31453400	-3.73893500	-1.67800300

AmAl(I)

C	3.92595600	1.68606600	1.17160100
H	4.11844300	2.43299400	1.93681600
C	-5.05479200	-0.59288100	-0.28647000
N	-1.06398700	0.53543200	-0.95303600
C	-2.83203200	2.50531900	-0.01665500
H	-1.80317100	2.43392500	0.36832900
N	1.14441200	0.46244100	-0.94390200
C	-1.84238100	-2.30909100	-1.37466600
H	-0.82969700	-1.89094700	-1.43386400
C	-2.21162400	-2.79786700	-2.77475400
C	-4.63607200	0.71607900	-0.06473800
H	-5.35427200	1.45785900	0.27516500
C	2.64012200	1.56095600	0.64416700
C	3.21373000	-1.28317200	-1.87562700
H	2.14620600	-1.54642600	-1.82879000
C	3.46482500	-0.25024100	-0.79713700
C	-4.13607900	-1.54577600	-0.70094500
H	-4.46268300	-2.57037000	-0.87117100
C	-2.79182800	-1.22229000	-0.91068100
C	1.52382500	2.50044700	1.06467000
H	0.60260800	1.91292200	1.17480000
C	4.01756600	-2.56487500	-1.68411000
C	-1.79790900	-3.47156800	-0.38371300
C	4.73602700	-0.07773900	-0.24735800
H	5.55358100	-0.70968400	-0.58625300
C	1.76192100	3.17802200	2.40824400
C	2.41475000	0.57487200	-0.34526500
C	-3.30771900	1.08722900	-0.26087100
C	1.26041200	3.55275100	-0.01410700
C	4.96946000	0.87845000	0.73235600
C	-2.38333900	0.10818500	-0.69343800
C	0.02298200	0.12565900	-0.28981800
C	3.47484000	-0.68784900	-3.25973700
C	-3.66016100	3.25880900	1.01654200
C	-2.76947000	3.29343300	-1.32554900
C	0.00065400	-0.64989700	0.96658100
C	0.92809900	-1.67883800	1.16980900
C	-0.94626700	-0.38720300	1.96435800
C	0.89623000	-2.43460300	2.33352300
H	1.66344400	-1.89327400	0.39874600
C	-0.95995900	-1.13701300	3.13241000
H	-1.66525100	0.41634600	1.82493000
H	1.61717100	-3.23706700	2.47307100
H	-1.69361100	-0.91497100	3.90404000
C	-0.04687600	-2.17845200	3.33483100
C	-0.09496500	-3.01149300	4.57776600
H	-0.76701400	-3.86833900	4.45248500
H	0.88882800	-3.41286100	4.83449500
H	-0.46426300	-2.44075000	5.43385600
Al	0.08984000	1.09251400	-2.61808400
H	-3.19176700	4.21967400	1.25123900
H	-4.66925400	3.47832500	0.65058700
H	-3.76161300	2.69506500	1.94961600
H	-6.09457000	-0.86698200	-0.13114700
H	-2.13213400	2.80008200	-2.06888300
H	-3.76841400	3.38956300	-1.76551100
H	-2.37473600	4.30223000	-1.16280700
H	-1.04249000	-4.20743900	-0.67913000
H	-1.55942700	-3.12785400	0.62770800
H	-2.76178400	-3.99109000	-0.34060300
H	-1.51066900	-3.56456400	-3.12098500
H	-3.21500300	-3.23825300	-2.78496000
H	-2.20576900	-1.97917400	-3.50099200
H	3.88710000	-2.98367600	-0.68083000
H	3.70867200	-3.32288400	-2.41026200
H	5.08988200	-2.39952100	-1.83448100
H	2.86351500	0.20376300	-3.43891800
H	4.52516500	-0.39064200	-3.35846900
H	3.25301300	-1.41077800	-4.05205000
H	0.87478200	3.74417400	2.70754200
H	1.98062500	2.45102000	3.19614900
H	2.59512400	3.88808300	2.36565300
H	0.43226700	4.21088800	0.27199500
H	2.14753900	4.17495400	-0.17462000
H	1.00565200	3.09882700	-0.98076200
H	5.96352600	0.99648700	1.15460200

AmAl(I)-d

C	-3.43694200	-3.47363700	-2.87676000
H	-3.75865700	-3.57514400	-3.91201100
C	-3.99737400	4.42919800	2.57306600
N	-2.77155600	1.31552600	0.02193900
C	-3.41740900	3.83795100	-1.15299900
H	-3.60333400	2.87020300	-1.64299100
N	-2.52547100	-0.82149300	-0.44189000
C	-2.88123200	0.80934800	2.92156900
H	-2.48915100	0.14230200	2.14347100

C	-1.76980200	0.97430600	3.95731000
C	-3.91940000	4.63575700	1.20001600
H	-4.17580900	5.60899100	0.78865200
C	-3.26968000	-2.19159900	-2.34270900
C	-2.24810000	-3.05768400	1.24715200
H	-2.76144100	-2.15501200	1.61196600
C	-2.63913000	-3.22528600	-0.20732300
C	-3.66831400	3.18754600	3.09866600
H	-3.72044000	3.02694100	4.17421600
C	-3.25824300	2.13247300	2.27946900
C	-3.56152400	-0.98451500	-3.21147300
H	-3.37618600	-0.07596700	-2.62306200
C	-2.68134600	-4.22027700	2.13119500
C	-4.09812900	0.13546000	3.55793700
C	-2.81920100	-4.48125600	-0.78282900
H	-2.66494300	-5.37299100	-0.18070800
C	-5.02958200	-0.95872400	-3.63656600
C	-2.84630000	-2.07855500	-1.00619800
C	-3.52913300	3.61062900	0.33952100
C	-2.63549100	-0.93141900	-4.42476300
C	-3.20391800	-4.60938700	-2.11499100
C	-3.20530600	2.34939600	0.88517700
C	-3.40493200	0.13161800	-0.10224000
C	-0.75025400	-2.80520200	1.39580500
C	-4.42559100	4.84183000	-1.69864600
C	-1.99150700	4.25126500	-1.51201800
C	-4.85820100	-0.05094800	0.02529200
C	-5.41625700	-1.26381500	0.45529500
C	-5.72481600	0.99833700	-0.31325400
C	-6.79276400	-1.41316100	0.54751400
H	-4.76759500	-2.08869800	0.73194000
C	-7.09981200	0.83632500	-0.22804400
H	-5.31066300	1.93775700	-0.66628000
H	-7.20631000	-2.35989200	0.88780900
H	-7.75471700	1.65846400	-0.50841000
C	-7.66047600	-0.36882500	0.20902300
C	-9.14328900	-0.52831900	0.33542000
H	-9.48619900	-0.24664900	1.33778800
H	-9.45474800	-1.56346200	0.17214100
H	-9.68081800	0.10572700	-0.37453100
Al	-1.07268600	0.58207100	-0.75483900
H	-4.38203700	4.87243500	-2.79156000
H	-4.22208000	5.85816200	-1.34412500
H	-5.45093700	4.59055500	-1.40751600
H	-4.31040900	5.23609400	3.22995300
H	-1.26347600	3.51880500	-1.14754300
H	-1.73872100	5.21555300	-1.05591200
H	-1.85827000	4.34268300	-2.59571800
H	-3.83975100	-0.86030300	3.93724100
H	-4.92502200	0.02565600	2.84980600
H	-4.46709900	0.72468300	4.40575700
H	-1.48144800	-0.00221300	4.36387000
H	-2.09394800	1.59637100	4.79944000
H	-0.87700900	1.43227900	3.51924100
H	-3.74498100	-4.45230400	2.01165900
H	-2.50381400	-3.98117800	3.18431000
H	-2.11542200	-5.13242200	1.91167900
H	-0.43026400	-1.92403000	0.82618600
H	-0.16547200	-3.65905200	1.03346900
H	-0.48324000	-2.63650500	2.44496000
H	-5.25494100	-0.05105700	-4.20626200
H	-5.69867000	-0.99149800	-2.77072100
H	-5.27084500	-1.81651300	-4.27477200
H	-2.84319000	-0.04726900	-5.03648300
H	-2.76561700	-1.81304900	-5.06271200
H	-1.58460000	-0.89257100	-4.12090700
C	2.29942400	-4.36588400	-0.17379300
H	2.15366400	-5.03899300	-1.01703100
C	5.32944700	4.67077000	-0.57548600
N	2.83771500	1.29925600	-0.18931100
C	2.65682700	2.82755800	-2.58257100
H	2.50853400	1.73900800	-2.53339000
N	2.36799500	-0.72723400	0.54673100
C	4.71360600	1.93824800	1.99631800
H	4.14457800	1.01154200	1.87310900
C	4.08291900	2.68047200	3.17394600
C	4.44978200	4.32270200	-1.59484700
H	4.39990300	4.93366300	-2.49286300
C	2.26895200	-2.98633900	-0.39787300
C	2.92978300	-1.70192400	3.16177100
H	3.37887200	-0.78926400	2.74278700
C	2.67988300	-2.64351600	1.99873300
C	5.40334400	3.88309200	0.56466500
H	6.09142900	4.15370800	1.36360300
C	4.60739800	2.74438200	0.71629700
C	2.01148700	-2.46815000	-1.79839100
H	2.04480400	-1.37121900	-1.77518000
C	3.90967200	-2.25515000	4.19076000
C	6.15367900	1.53620900	2.30639200
C	2.68947300	-4.02672400	2.17356100
H	2.86360100	-4.43816000	3.16494900
C	3.08430500	-2.94707300	-2.77524800
C	2.45386000	-2.12752600	0.70158300
C	3.63474800	3.19652900	-1.48842200
C	0.61551300	-2.86847600	-2.27477100
C	2.49876100	-4.88804900	1.09706500
C	3.71070200	2.40857700	-0.32067300
C	3.29276000	0.04139700	-0.03962100
C	1.62477600	-1.28229900	3.83723300
C	3.14425900	3.17560500	-3.98372100
C	1.30143400	3.47232600	-2.30357000
C	4.62552900	-0.40964400	-0.47406500
C	5.37797800	-1.31792800	0.28031600
C	5.15558000	0.06193400	-1.68310000
C	6.62732100	-1.73386900	-0.16252600
H	4.99025900	-1.68533200	1.22614800
C	6.39810100	-0.36847700	-2.12329200
H	4.57236200	0.75464900	-2.28441800
H	7.20310400	-2.43367200	0.43903300
H	6.78879300	-0.00139800	-3.06983200
C	7.15955700	-1.27003100	-1.36935600
C	8.51349600	-1.70618500	-1.83637100
H	9.26330800	-0.92624200	-1.66127800
H	8.85207100	-2.60429800	-1.31402500
H	8.52257100	-1.91478700	-2.91015700
Al	1.10416300	0.85200600	0.77439500
H	2.45659100	2.77795900	-4.73616600
H	3.19695700	4.25852100	-4.14170300
H	4.13939100	2.76485300	-4.18419900
H	5.95731700	5.55224600	-0.67270000
H	0.94461000	3.21459100	-1.30043800
H	1.36916100	4.56584500	-2.35681400
H	0.54370900	3.14015700	-3.02130800
H	6.18636300	0.84831600	3.15931500
H	6.62317900	1.03834300	1.45190400
H	6.76844400	2.40525500	2.56616000
H	4.11267100	2.06532500	4.08121700
H	4.61585900	3.61497600	3.38419600
H	3.03638700	2.93343100	2.97392800
H	4.84301000	-2.59255500	3.72771700
H	4.15808900	-1.48547800	4.92861300
H	3.49000000	-3.10259900	4.74385900

H	0.92553200	-0.83421700	3.12202500
H	1.12707000	-2.14730300	4.29314500
H	1.80775200	-0.54248900	4.62524400
H	2.93344100	-2.50537700	-3.76579700
H	4.08934200	-2.67968700	-2.43334100
H	3.05398100	-4.03623100	-2.89498100
H	0.41760400	-2.48568100	-3.28183100
H	0.49873900	-3.95846000	-2.30409200
H	-0.16501400	-2.47907800	-1.61107800
H	2.51334700	-5.96371100	1.25026900
H	-3.33452100	-5.59574300	-2.55178100

B

N	-1.41090200	-0.39411100	0.41319000
C	-1.23358700	-0.51241000	1.73176500
C	0.03151100	-0.62291800	2.32798700
H	0.04366200	-0.75531700	3.40360600
C	1.27911000	-0.42040800	1.71549700
N	1.42669800	-0.26194400	0.39702800
C	-2.43322300	-0.48456000	2.63059800
H	-2.94578300	0.48101800	2.56273000
H	-2.15828800	-0.65896500	3.67112000
H	-3.16823100	-1.23586700	2.32305700
C	2.48604500	-0.35906300	2.60486700
H	3.11276500	-1.24606300	2.46043700
H	2.20269800	-0.30892700	3.65671300
H	3.11577100	0.50204900	2.36238000
C	-2.70696800	-0.05955400	-0.09822700
C	-3.05393000	1.30049600	-0.22434500
C	-4.29961900	1.61822000	-0.76898100
H	-4.57912400	2.66528100	-0.87194700
C	-5.17832600	0.62607100	-1.18569400
C	-4.81460300	-0.70835700	-1.06470300
H	-5.49771600	-1.48711400	-1.39892800
C	-3.58067500	-1.07796600	-0.52287600
C	-2.11687700	2.41640800	0.19642500
H	-1.20965500	1.96222600	0.61554900
C	-3.21652100	-2.54616400	-0.42682500
H	-2.24713600	-2.62353100	0.08018800
C	2.71262000	0.06679100	-0.14433500
C	3.67430800	-0.93953100	-0.35655500
C	4.90307200	-0.57163400	-0.91224500
H	5.65429700	-1.33946200	-1.08608600
C	5.17324000	0.74509000	-1.25373500
C	4.20117100	1.72093300	-1.06476600
H	4.41217500	2.74613200	-1.35525600
C	2.95517100	1.40635800	-0.51951000
C	3.40459700	-2.40229100	-0.06054700
H	2.46037000	-2.47437100	0.49316900
C	1.89511100	2.47483900	-0.32158400
H	0.92069500	2.00222800	-0.50690000
H	-6.14262000	0.89333100	-1.60897500
H	6.13577000	1.01192500	-1.68139100
C	2.02765800	3.63129300	-1.30468700
H	1.17243700	4.30684800	-1.21212200
H	2.92620800	4.23000900	-1.11816500
H	2.06539100	3.27797700	-2.33964400
C	1.87607000	2.99616600	1.11647400
H	1.62853600	2.20942100	1.83559900
H	2.84919600	3.41693400	1.39579400
H	1.12716900	3.78860100	1.22944700
C	3.23249600	-3.18549900	-1.36283100
H	4.15047300	-3.14862000	-1.96083200
H	3.00896200	-4.23747700	-1.15787000
H	2.41687300	-2.78292600	-1.96943300
C	4.50497900	-3.03491800	0.79038200
H	5.45380600	-3.08300800	0.24496100
H	4.69206900	-2.47652300	1.71353100
H	4.23902600	-4.06044300	1.06438600
C	-4.24146000	-3.33238200	0.38923500
H	-4.37378200	-2.91823200	1.39418900
H	-5.22424900	-3.32969500	-0.09535200
H	-3.93346000	-4.37726600	0.49495600
C	-3.05999300	-3.15827600	-1.81834700
H	-2.75579700	-4.20756200	-1.75004700
H	-4.00541600	-3.11976500	-2.37198000
H	-2.30476900	-2.63049200	-2.40763600
C	-2.73956000	3.29884400	1.27743800
H	-3.63155800	3.81499300	0.90477800
H	-3.04083000	2.72150900	2.15753900
H	-2.03145200	4.06597400	1.60762900
C	-1.69588000	3.26006600	-1.00586700
H	-2.56416100	3.72602000	-1.48565000
H	-1.02243800	4.06618600	-0.69375900
H	-1.17931100	2.65502800	-1.75793600
Al	0.01291500	-0.66037500	-0.87625300
H	0.08349800	-2.19018800	-1.30113800
H	-0.05016100	0.45150900	-2.00501100

5

N	1.42703800	-0.49005600	0.04574600
C	1.25862900	-1.80846500	0.17602800
C	0.00000000	-2.42469300	0.23530000
H	0.00000000	-3.50314800	0.34207600
C	-1.25862900	-1.80846500	0.17602700
N	-1.42703800	-0.49005600	0.04574400
C	2.47214300	-2.68720000	0.26599800
H	3.10254800	-2.57691100	-0.62244700
H	2.19722200	-3.73697700	0.37188900
H	3.10046000	-2.40083600	1.11576800
C	-2.47214300	-2.68720100	0.26599700
H	-3.10045500	-2.40084100	1.11577200
H	-2.19722100	-3.73697800	0.37188200
H	-3.10255200	-2.57690700	-0.62244500
C	2.75452200	0.04796100	-0.00511900
C	3.38740000	0.20677400	-1.24837400
C	4.66274000	0.77238400	-1.27637100
H	5.15993700	0.89403700	-2.23812400
C	5.31329100	1.18590000	-0.11311400
C	4.65585500	1.01388600	1.10589000
H	5.14758900	1.32641400	2.02648700
C	3.38044300	0.45353000	1.18462700
C	-2.75452200	0.04796100	-0.00512000
C	-3.38044300	0.45352800	1.18462700
C	-4.65585500	1.01388400	1.10589000
H	-5.14759000	1.32641100	2.02648700
C	-5.31329100	1.18590000	-0.11311400
C	-4.66274000	0.77238500	-1.27637100
H	-5.15993600	0.89404000	-2.23812400
C	-3.38740000	0.20677500	-1.24837500
Al	0.00000000	0.82102600	-0.09135100
H	-0.00000200	1.78405500	1.17213400
H	0.00000100	1.50120200	-1.52699000
C	2.70601000	-0.23131800	-2.50815200
H	2.46995000	-1.30219600	-2.49643500
H	1.75181600	0.28961300	-2.64694800
H	3.33152800	-0.03735600	-3.38259800
C	6.66802300	1.82386600	-0.17362500
H	7.24411100	1.47056800	-1.03336700

H	6.59209600	2.91350500	-0.26705500
H	7.25119500	1.62291600	0.72946300
C	2.69086500	0.27744300	2.50247700
H	1.73938900	0.82080400	2.52946000
H	2.44778100	-0.77316000	2.70174300
H	3.31354700	0.63777900	3.32464000
C	-2.69086500	0.27743900	2.50247600
H	-2.44777900	-0.77316400	2.70174000
H	-1.73939100	0.82080200	2.52946200
H	-3.31354900	0.63777100	3.32464000
C	-6.66802200	1.82386800	-0.17362300
H	-7.25120000	1.62290400	0.72945800
H	-6.59209400	2.91350800	-0.26703500
H	-7.24410500	1.47058300	-1.03337500
C	-2.70601000	-0.23131600	-2.50815400
H	-1.75181600	0.28961300	-2.64694800
H	-2.46995100	-1.30219500	-2.49643800
H	-3.33152700	-0.03735300	-3.38259900

mesBDIAlH2AlBDIdipp

N	-0.15064900	-2.78712900	-0.58693500
C	0.10409600	-3.95301300	0.01749600
C	1.19995500	-4.16551200	0.86354600
H	1.26838700	-5.14513200	1.32196900
C	2.28988200	-3.30730700	1.06943400
N	2.35458300	-2.06704500	0.58133000
C	-0.78555100	-5.13224600	-0.25811200
H	-1.83900700	-4.90065000	-0.07611900
H	-0.50185100	-5.98952300	0.35332000
H	-0.71985400	-5.42407700	-1.31204500
C	3.45896300	-3.85650100	1.83626300
H	4.36057500	-3.87067700	1.21469100
H	3.26137100	-4.87076700	2.18485500
C	-1.22407400	-2.75021800	-1.53606400
C	-2.52500400	-2.46198200	-1.09542700
C	-3.55618000	-2.42691900	-2.03627000
H	-4.56662400	-2.19811800	-1.69729000
C	-3.32665400	-2.67614800	-3.38981600
C	-2.02487500	-2.97929300	-3.79233200
H	-1.82673300	-3.18844200	-4.84325700
C	-0.96055100	-3.01835200	-2.88998000
C	3.60210100	-1.36651600	0.68574900
C	4.50953000	-1.46525500	-0.38717100
C	5.71293000	-0.76779500	-0.30965300
H	6.41464500	-0.84158600	-1.14018200
C	6.04052900	0.02057300	0.79619500
C	5.13121100	0.08246100	1.84903300
H	5.36700600	0.69025100	2.72226200
C	3.91101000	-0.59783000	1.81874100
N	0.01099500	2.63663100	-0.35420800
C	-0.34796600	3.80290400	0.17264600
C	-0.95768600	3.89623800	1.44113600
H	-1.11114500	4.90022700	1.82206900
C	-1.58283000	2.86807400	2.15388800
N	-1.46911100	1.56346300	1.83435500
C	-0.11827700	5.08305700	-0.57829700
H	0.91550500	5.42078100	-0.44066500
H	-0.77769900	5.87601700	-0.22165100
H	-0.26317000	4.95031500	-1.65359900
C	-2.45289400	3.27490600	3.31064500
H	-3.51501600	3.25543800	3.04862800
H	-2.20950900	4.28804700	3.63485600
H	-2.33245800	2.58749800	4.15329700
C	0.55595700	2.56707000	-1.67455300
C	1.90031800	2.91016400	-1.90742100
C	2.40804100	2.75469000	-3.20046500
H	3.44937800	3.00753200	-3.39078500
C	1.61655800	2.27330800	-4.23261600
C	0.29184300	1.93260100	-3.98371600
H	-0.32269100	1.54824200	-4.79301400
C	-0.25970000	2.06727900	-2.70951200
C	2.82370900	3.37525300	-0.80024100
H	2.22467900	3.56015100	0.09965200
C	-1.71407200	1.73581100	-2.44457400
H	-1.77618600	1.32268000	-1.42497100
C	-2.46359300	0.68365300	2.35463900
C	-3.79651000	0.79167400	1.89192900
C	-4.77655000	-0.01808400	2.46773500
H	-5.80334500	0.06739800	2.11580300
C	-4.46361300	-0.94499500	3.45376300
C	-3.14132100	-1.09280900	3.84626400
H	-2.88215600	-1.83921500	4.59499600
C	-2.12553900	-0.29642900	3.30949900
C	-4.18689100	1.66115900	0.70879900
H	-3.32579300	2.27720300	0.42198700
C	-0.70117500	-0.54806500	3.76166400
H	-0.03786800	0.15422600	3.23802500
H	2.03091300	2.15643700	-5.23036100
H	-5.24094200	-1.56246800	3.89534100
C	-0.28038200	-1.97312300	3.39711600
H	0.73232700	-2.19579800	3.74917000
H	-0.29844600	-2.13296700	2.31217300
C	-0.52005200	-0.28867000	5.25564300
H	-0.80223900	0.73383800	5.52434200
H	-1.13281400	-0.97047900	5.85654400
H	0.52441200	-0.43740200	5.54999100
C	-4.52408500	0.75386200	-0.47663400
H	-5.34765900	0.07455000	-0.22343400
H	-4.83658000	1.33649200	-1.35008800
H	-3.66825300	0.13761600	-0.77028300
C	-5.36222100	2.59236400	0.99761700
H	-6.27799400	2.02949700	1.21070400
H	-5.17790600	3.25044900	1.85207900
H	-5.56755900	3.22744600	0.12951700
C	-2.56061000	3.01126100	-2.47301500
H	-2.26376400	3.71721600	-1.69100300
H	-2.45869100	3.51865700	-3.43960200
H	-3.62257400	2.78571200	-2.32737100
C	-2.27785000	0.69370900	-3.39986300
H	-3.28102200	0.39037600	-3.08363700
H	-2.36684500	1.08117100	-4.42170000
H	-1.65294400	-0.20527400	-3.43143500
C	3.55758300	4.66556300	-1.16124100
H	4.25263800	4.51036800	-1.99396200
H	2.87066000	5.46465300	-1.45954100
H	4.14753500	5.02515500	-0.31217700
C	3.82284100	2.27616000	-0.44928800
H	4.48187600	2.05404300	-1.29785100
H	4.45054600	2.57271600	0.39791600
H	3.31882700	1.34358700	-0.17324700
H	-0.95301700	-2.71221600	3.84732800
H	3.69738200	-3.22846500	2.70025100
Al	0.89718900	-1.12747900	-0.37746400
Al	0.19511300	1.09282500	0.87665900
H	1.44338600	-0.76685300	-1.84218900
H	1.33575800	1.66720800	1.86524300
C	4.19161900	-2.32256400	-1.57334000
H	3.95283900	-3.35266400	-1.28150100
H	3.31189100	-1.94484300	-2.10733300
H	5.03112400	-2.35477600	-2.27203600

C	7.31347100	0.80996000	0.82470000
H	8.14457300	0.25985200	0.37321900
H	7.21063200	1.74538700	0.26068200
H	7.60094700	1.08000800	1.84444900
C	2.98072700	-0.52233200	2.98836800
H	3.02552600	0.45806800	3.46962000
H	1.94753300	-0.69194000	2.68200600
H	3.22514400	-1.27476000	3.75125300
C	0.42722000	-3.34538200	-3.34748000
H	1.08214300	-2.47033900	-3.25915200
H	0.88805500	-4.13142700	-2.73746000
H	0.43235600	-3.67490400	-4.38930900
C	-4.43682200	-2.57907300	-4.39110100
H	-4.46985700	-1.58257800	-4.84892500
H	-4.31350600	-3.29733500	-5.20688000
H	-5.41408700	-2.75246700	-3.93217900
C	-2.78515800	-2.22768600	0.35853400
H	-2.15587600	-1.41911200	0.75007300
H	-3.82757000	-1.96069200	0.54880300
H	-2.54843100	-3.10742500	0.97064100

mesBDIAl(I)

N	1.39818300	-0.46943700	-0.03461400
C	1.25207700	-1.79716400	-0.11370800
C	-0.00000300	-2.42121400	-0.15152600
H	-0.00000700	-3.50352900	-0.21664400
C	-1.25207900	-1.79715500	-0.11371400
N	-1.39817600	-0.46942700	-0.03462100
C	2.47097200	-2.67430100	-0.16528800
H	3.10320600	-2.41755800	-1.02163400
H	2.20149000	-3.72928000	-0.23344700
H	3.09701100	-2.52928200	0.72134300
C	-2.47098000	-2.67428400	-0.16529000
H	-3.09700700	-2.52927300	0.72135100
H	-2.20150600	-3.72926400	-0.23346600
H	-3.10322400	-2.41752700	-1.02162500
C	2.73051500	0.05788500	0.00121500
C	3.36501600	0.41655000	-1.19793700
C	4.64948500	0.95752700	-1.13720600
H	5.14679100	1.23239800	-2.06682400
C	5.30824700	1.16030100	0.07621900
C	4.64514000	0.80517900	1.25171500
H	5.13895900	0.95973200	2.21054600
C	3.36061000	0.26132200	1.23860400
C	-2.73050600	0.05790300	0.00120500
C	-3.36060300	0.26134200	1.23859200
C	-4.64513300	0.80520100	1.25169900
H	-5.13895300	0.95975600	2.21053000
C	-5.30823600	1.16032300	0.07620200
C	-4.64947100	0.95754700	-1.13722200
H	-5.14677400	1.23241800	-2.06684000
C	-3.36500300	0.41656900	-1.19794900
Al	0.00000900	0.99234000	0.04883400
C	2.66503400	0.22427300	-2.50756100
H	2.38786400	-0.82245300	-2.68091300
H	1.72684600	0.79412800	-2.54112500
H	3.28762500	0.55151900	-3.34343000
C	6.67422300	1.77546200	0.11803900
H	7.24911300	1.54731300	-0.78387300
H	6.61812300	2.86781400	0.19188100
H	7.24884400	1.42808000	0.98128600
C	2.65575200	-0.09428100	2.51095300
H	1.72027500	0.47125800	2.61593100
H	2.37313100	-1.15314300	2.54791600
H	3.27746000	0.11982500	3.38333600
C	-2.65574800	-0.09426000	2.51094400
H	-2.37312000	-1.15312000	2.54790500
H	-1.72027600	0.47128600	2.61592700
H	-3.27746200	0.11984000	3.38332500
C	-2.66502000	0.22428500	-2.50757100
H	-1.72679600	0.79408100	-2.54111100
H	-2.38791300	-0.82245500	-2.68094800
H	-3.28758300	0.55158900	-3.34343700
C	-6.67421100	1.77548500	0.11801800
H	-6.61811000	2.86783700	0.19185200
H	-7.24910100	1.54733000	-0.78389300
H	-7.24883300	1.42810900	0.98126700

mesBDIAl(I)-d

N	-1.46439100	-2.50732000	0.30878100
C	-1.31637700	-3.54453500	1.14319800
C	-0.08413700	-3.93803000	1.67114600
H	-0.10059300	-4.79621400	2.33258100
C	1.18100400	-3.43686600	1.34405300
N	1.37836900	-2.37482300	0.55129700
C	-2.50454900	-4.39656500	1.49768100
H	-3.30679100	-3.81090000	1.95547800
H	-2.22091500	-5.19575800	2.18359400
H	-2.93912200	-4.84749100	0.59910100
C	2.35856800	-4.20459000	1.88269700
H	2.97596000	-4.59108200	1.06476200
H	2.02743500	-5.04485700	2.49404500
C	-2.77714800	-2.33021000	-0.24000500
C	-3.75224900	-1.63703000	0.49181000
C	-5.04806500	-1.57979300	-0.02297800
H	-5.81493700	-1.06747800	0.55703200
C	-5.38189400	-2.13435700	-1.25834800
C	-4.37921500	-2.78640900	-1.97873000
H	-4.61670300	-3.22982100	-2.94538100
C	-3.08017900	-2.90843200	-1.48458100
C	2.75399000	-2.08834000	0.25250100
C	3.32142100	-2.62546600	-0.91472200
C	4.69490200	-2.48180000	-1.11559800
H	5.14106800	-2.91188800	-2.01228800
C	5.51190900	-1.83925400	-0.18498100
C	4.91074200	-1.28570600	0.94546600
H	5.52591600	-0.75350200	1.67129500
C	3.53760400	-1.37483000	1.17436800
N	1.37836200	2.37482500	-0.55129300
C	1.18099500	3.43687000	-1.34404700
C	-0.08414600	3.93803300	-1.67113900
H	-0.10060200	4.79621800	-2.33257300
C	-1.31638600	3.54453500	-1.14319400
N	-1.46439900	2.50731900	-0.30877800
C	2.35855900	4.20459600	-1.88269000
H	2.97595400	4.59108100	-1.06475400
H	2.02742600	5.04486700	-2.49403100
H	3.02095100	3.57578500	-2.48403700
C	-2.50455900	4.39656300	-1.49767800
H	-3.30679700	3.81089600	-1.95548100
H	-2.22092500	5.19575900	-2.18358700
H	-2.93913800	4.84748400	-0.59909800
C	2.75398300	2.08834300	-0.25250000
C	3.32141500	2.62546500	0.91472500
C	4.69489600	2.48180200	1.11559700
H	5.14106300	2.91188800	2.01228800
C	5.51190400	1.83926300	0.18497600
C	4.91073600	1.28571600	-0.94547100

H	5.52591000	0.75351600	-1.67130300
C	3.53759700	1.37483800	-1.17437100
C	-2.77715600	2.33020600	0.24000600
C	-3.75225600	1.63702400	-0.49181200
C	-5.04807200	1.57978400	0.02297400
H	-5.81494200	1.06746800	-0.55703800
C	-5.38190500	2.13434800	1.25834300
C	-4.37922800	2.78640200	1.97872700
H	-4.61671900	3.22981300	2.94537800
C	-3.08019100	2.90842600	1.48458100
H	3.02096200	-3.57577600	2.48403800
Al	-0.02874400	-1.29405500	-0.39556000
Al	-0.02875100	1.29405500	0.39556100
C	-2.03084300	3.65968900	2.24405100
H	-1.18430000	3.00638000	2.49931300
H	-1.60838300	4.48277500	1.65451800
H	-2.43175000	4.07561500	3.17156900
C	-6.76574000	1.98942600	1.81532000
H	-6.84329300	1.10987200	2.46737400
H	-7.05711500	2.85434500	2.41828300
H	-7.50925400	1.86338800	1.02292900
C	-3.40886100	0.98396200	-1.79498300
H	-2.56914000	0.28560200	-1.67952300
H	-4.25857600	0.41991300	-2.18855700
H	-3.09499500	1.70648900	-2.55805500
C	2.91995600	0.76540100	-2.39536600
H	3.64261200	0.14243200	-2.92924500
H	2.05512600	0.14071100	-2.13502700
H	2.54424300	1.52126500	-3.09660900
C	6.98561300	1.71189300	0.42158600
H	7.39033700	2.58562300	0.94090900
H	7.21871700	0.83727600	1.04310000
H	7.53802800	1.58912900	-0.51486000
C	2.47562300	3.37574300	1.89692900
H	1.95747500	4.22328200	1.43110800
H	1.68650300	2.73272000	2.31247900
H	3.07521300	3.75760900	2.72699100
C	6.98561800	-1.71188000	-0.42159400
H	7.21871800	-0.83726100	-1.04310500
H	7.53803400	-1.58911900	0.51485200
H	7.39034200	-2.58560800	-0.94092100
C	2.47562900	-3.37575000	-1.89692200
H	1.95748400	-4.22328800	-1.43109600
H	1.68650800	-2.73273000	-2.31247400
H	3.07521900	-3.75761800	-2.72698300
C	2.91996300	-0.76539100	2.39536200
H	3.64261800	-0.14241900	2.92923800
H	2.05513200	-0.14070300	2.13502200
H	2.54425000	-1.52125400	3.09660700
C	-3.40885900	-0.98396700	1.79498200
H	-2.56913600	-0.28560900	1.67952500
H	-4.25857500	-0.41991600	2.18855200
H	-3.09499800	-1.70649300	2.55805600
C	-2.03082900	-3.65969300	-2.24404800
H	-1.18428600	-3.00638300	-2.49931000
H	-1.60836800	-4.48277800	-1.65451400
H	-2.43173300	-4.07562100	-3.17156700
C	-6.76572800	-1.98943600	-1.81532800
H	-6.84327200	-1.10990200	-2.46741000
H	-7.05711400	-2.85437100	-2.41826400
H	-7.50924000	-1.86336400	-1.02294000

7

N	-1.47282600	-2.51305600	0.28141600
C	-1.32865100	-3.52593200	1.14384500
C	-0.10895200	-3.88626200	1.72497500
H	-0.13947700	-4.71787200	2.41861100
C	1.16268800	-3.38752200	1.41893200
N	1.38446200	-2.36335400	0.58624700
C	-2.51424600	-4.38694600	1.48521900
H	-3.33771000	-3.80456200	1.90819300
H	-2.23692500	-5.16597800	2.19601500
H	-2.91736600	-4.86526600	0.58619800
C	2.32679400	-4.11843300	2.03179400
H	2.94724000	-4.58040000	1.25623600
H	1.98072100	-4.90095300	2.70776900
C	-2.78183500	-2.34696300	-0.27675700
C	-3.75286800	-1.63846100	0.44772600
C	-5.05050600	-1.58508400	-0.06118000
H	-5.81406000	-1.06324000	0.51445300
C	-5.39009900	-2.16168900	-1.28536100
C	-4.39489800	-2.83885300	-1.99228100
H	-4.64149100	-3.30904100	-2.94386900
C	-3.09380500	-2.96123800	-1.50132700
C	2.76499400	-2.09943700	0.29624100
C	3.36229600	-2.74363100	-0.80030500
C	4.73941700	-2.60830600	-0.97994700
H	5.21001600	-3.11901400	-1.82003400
C	5.53114400	-1.87706600	-0.09347800
C	4.90241800	-1.23126100	0.97090700
H	5.49840700	-0.63567400	1.66254500
C	3.52406400	-1.30663900	1.17229100
N	1.38892500	2.37105000	-0.56766800
C	1.16888000	3.40404200	-1.39039400
C	-0.10148700	3.90724800	-1.69411100
H	-0.12934800	4.74537900	-2.37994000
C	-1.32428500	3.53906500	-1.12464300
N	-1.47170900	2.51947300	-0.27088300
C	2.33432500	4.13727100	-1.99816800
H	2.96558200	4.57833100	-1.21927000
H	1.98956400	4.93526300	-2.65644500
H	2.98695700	3.46976100	-2.56777100
C	-2.51053600	4.39732600	-1.47064900
H	-3.32654600	3.81363700	-1.90625200
H	-2.23016000	5.18290500	-2.17299400
H	-2.92539200	4.86687700	-0.57238700
C	2.77045600	2.09692700	-0.29049700
C	3.37909000	2.72352400	0.80964700
C	4.75768200	2.58270300	0.97497400
H	5.23678500	3.08029400	1.81815600
C	5.54007300	1.86463800	0.06986300
C	4.90027500	1.23573800	-0.99820600
H	5.48929200	0.65136700	-1.70518600
C	3.52028100	1.31548700	-1.18506500
C	-2.78447300	2.34669600	0.27650600
C	-3.74763300	1.63733900	-0.45746000
C	-5.04954200	1.58041500	0.04015700
H	-5.80699600	1.05807100	-0.54302400
C	-5.40087600	2.15400500	1.26240400
C	-4.41303400	2.83136300	1.97945100
H	-4.66864000	3.29884200	2.92999800
C	-3.10816200	2.95759500	1.49975200
H	2.99008000	-3.44611200	2.58286200
Al	-0.02526300	-1.29000600	-0.31913000
Al	-0.02457800	1.29768000	0.33368400
H	0.13620500	-1.56853900	-1.89694700
H	0.12914400	1.57610300	1.91234700
C	2.86235300	0.63397500	-2.34552000
H	3.57583100	0.00867000	-2.88846200
H	2.03282800	-0.00808700	-2.02660700

H	2.42880800	1.34822400	-3.05665900
C	2.57639000	3.57564600	1.74404500
H	2.05136800	4.37840600	1.21134800
H	1.79714400	2.99288200	2.24862300
H	3.21379900	4.03512500	2.50357400
C	7.02004800	1.74342200	0.26733400
H	7.44455100	2.64515900	0.71836600
H	7.26948300	0.90761200	0.93411300
H	7.54183700	1.55956800	-0.67666600
C	-2.08956400	3.77253800	2.23554200
H	-1.20654100	3.17654900	2.49214500
H	-1.72344300	4.60573500	1.62234800
H	-2.50814300	4.19107000	3.15406100
C	-6.78990500	2.00747500	1.80558400
H	-6.88328800	1.10409700	2.42207000
H	-7.07230800	2.85268400	2.43958600
H	-7.53074900	1.92198800	1.00521200
C	-3.38667000	0.97959200	-1.75398800
H	-2.52570400	0.30995200	-1.63415700
H	-4.21828300	0.38365400	-2.13885700
H	-3.09882700	1.70398800	-2.52565400
C	7.00913100	-1.75945600	-0.30748300
H	7.25307900	-0.92569900	-0.97882400
H	7.54167900	-1.57441200	0.63029800
H	7.42669400	-2.66323500	-0.76084600
C	2.54881900	-3.60679600	-1.71514600
H	2.02663700	-4.40079800	-1.16665600
H	1.76670800	-3.02873100	-2.22079300
H	3.17800500	-4.07858900	-2.47395700
C	2.87939900	-0.61276400	2.33317900
H	3.56982400	0.09604900	2.79814300
H	1.98159400	-0.05991100	2.03450400
H	2.55050900	-1.31816200	3.10729200
C	-3.40424400	-0.97731300	1.74593100
H	-2.54738200	-0.30168100	1.63057800
H	-4.24214400	-0.38682900	2.12541100
H	-3.11587600	-1.69891700	2.51992900
C	-2.06667600	-3.77339800	-2.22828900
H	-1.19332700	-3.16871500	-2.49742100
H	-1.68559400	-4.59140000	-1.60399600
H	-2.48254300	-4.21130000	-3.13897300
C	-6.77461900	-2.01868100	-1.84085700
H	-6.86594600	-1.11315100	-2.45449500
H	-7.04762200	-2.86249200	-2.48078300
H	-7.52329800	-1.93939600	-1.04715200

9

N	-1.43676700	0.33298000	0.07776500
C	-1.26237800	1.65761800	0.07162800
C	-0.00000200	2.26508000	-0.00000200
H	-0.00000500	3.34922900	-0.00000100
C	1.26237400	1.65762500	-0.07163200
N	1.43676700	0.33298400	-0.07776800
C	-2.44990900	2.57391400	0.16599300
H	-2.94418900	2.69582600	-0.80248100
H	-2.14267700	3.56416600	0.50512500
H	-3.20309200	2.17878100	0.85243100
C	2.44990500	2.57392200	-0.16598700
H	2.94417600	2.69583100	0.80249200
H	2.14267700	3.56417600	-0.50511800
H	3.20309400	2.17879200	-0.85242100
C	-2.74281400	-0.23138500	0.06086000
C	-3.61153800	-0.02834000	-1.01749000
C	-4.85861400	-0.64202400	-1.03672900
H	-5.52369200	-0.47811000	-1.88013200
C	-5.24915100	-1.47547200	0.00942800
C	-4.37894600	-1.69727100	1.07318300
H	-4.67141900	-2.35091800	1.89011600
C	-3.13004900	-1.08335800	1.10090300
C	2.74281300	-0.23138200	-0.06086400
C	3.61153800	-0.02834200	1.01748600
C	4.85861400	-0.64202800	1.03672500
H	5.52369100	-0.47811400	1.88012900
C	5.24915000	-1.47547400	-0.00943300
C	4.37894500	-1.69726900	-1.07318900
H	4.67141700	-2.35091400	-1.89012400
C	3.13005000	-1.08335400	-1.10090800
H	-6.22227600	-1.95706700	-0.01037200
H	6.22227500	-1.95707000	0.01036700
Al	0.00000600	-0.99095500	0.00001100
H	0.09620300	-1.79999600	1.36084900
H	-0.09620200	-1.80003800	-1.36080000
H	3.28945300	0.59561400	1.84691900
H	2.44441200	-1.24994200	-1.92761900
H	-3.28945000	0.59561700	-1.84692000
H	-2.44441000	-1.24994800	1.92761300

10

N	-1.51559100	2.42455300	1.06002600
C	-1.80821800	3.62201200	0.54016100
C	-0.86919800	4.42161000	-0.12370900
H	-1.23968900	5.37810200	-0.47419600
C	0.50730900	4.21183700	-0.29949300
N	1.13160500	3.09225900	0.07947200
C	-3.18364000	4.21097300	0.69450700
H	-3.89621800	3.74700500	0.00512800
H	-3.16604000	5.28097500	0.48421900
H	-3.57969200	4.05443300	1.70104600
C	1.26028700	5.37370800	-0.89251000
H	2.26815800	5.46272000	-0.48291600
H	0.71804200	6.30155000	-0.70360900
C	-2.49989900	1.73881800	1.82782100
C	-3.70621400	1.31121500	1.26414100
C	-4.67509800	0.71028600	2.06034000
H	-5.61151700	0.39274300	1.60778500
C	-4.43811400	0.48968900	3.41504300
C	-3.21069700	0.85285100	3.96451200
H	-3.00467800	0.66425600	5.01488200
C	-2.24418300	1.47479900	3.17880500
C	2.53199300	2.94780200	-0.10627100
C	3.31947300	2.54365900	0.97958200
C	4.70019400	2.43505300	0.84208000
H	5.29701000	2.13279100	1.69859800
C	5.31355100	2.71841400	-0.37459900
C	4.52817700	3.05887300	-1.47426300
H	4.99046400	3.23059500	-2.44272300
C	3.14751800	3.15305200	-1.34912300
N	1.45124100	-2.02485300	-0.31864400
C	1.36822300	-3.16727900	-0.99014200
C	0.39434100	-3.38767100	-1.98705700
H	0.48020000	-4.31940300	-2.53620200
C	-0.77189200	-2.64940100	-2.19775300
N	-1.02359900	-1.47409900	-1.59176100
C	2.30355300	-4.30879400	-0.70478800
H	3.04119000	-4.39938100	-1.51000600
H	1.75600300	-5.25387800	-0.66676900
H	2.84820400	-4.17373800	0.23169300
C	-1.80180200	-3.22660200	-3.12862300

H	-2.73481200	-3.46544300	-2.60976700
H	-1.43367600	-4.13272900	-3.61171600
H	-2.06632600	-2.49534300	-3.90048300
C	2.45443500	-1.79306900	0.67242600
C	3.79053400	-1.57103700	0.28423500
C	4.70829100	-1.19256300	1.26871600
H	5.74002700	-0.99996700	0.97830300
C	4.32365300	-1.03877600	2.59243600
C	3.00528600	-1.28515300	2.96272400
H	2.71167000	-1.17274600	4.00234600
C	2.05114800	-1.66977600	2.02098500
C	4.26226300	-1.70412500	-1.15125900
H	3.43107700	-2.07396200	-1.76109200
C	0.63314600	-2.02075700	2.42715800
H	-0.04159900	-1.58352800	1.67291800
C	-2.38983500	-1.06887400	-1.51655200
C	-3.22617700	-1.70743400	-0.57665900
C	-4.58565600	-1.38953900	-0.57394700
H	-5.24360900	-1.89099000	0.13470800
C	-5.10427100	-0.43532700	-1.44132300
C	-4.25419100	0.23332100	-2.31555600
H	-4.65684900	0.99452400	-2.98083300
C	-2.89152600	-0.07315700	-2.37688800
C	-2.68292600	-2.69745600	0.43912600
H	-1.59598400	-2.75761300	0.30551600
C	-1.97757900	0.67034800	-3.32986800
H	-1.04989000	0.09061400	-3.42184900
H	6.39259200	2.64273400	-0.47609100
H	5.05024700	-0.73142800	3.34027500
H	-6.16588500	-0.20127700	-1.42568900
H	-5.19535700	0.01522600	4.03289200
C	-1.60922300	2.03466100	-2.74375500
H	-0.88221200	2.55861800	-3.37463200
H	-1.17188300	1.93981700	-1.74345000
C	-2.57037900	0.82651400	-4.72688200
H	-2.86975400	-0.13598200	-5.15338600
H	-3.45401900	1.47448500	-4.72464600
H	-1.84100700	1.28247000	-5.40321800
C	-2.94118200	-2.19874400	1.85872000
H	-4.01334700	-2.10886300	2.06620600
H	-2.52228800	-2.89150500	2.59720400
H	-2.49659800	-1.21135400	2.02573600
C	-3.25543100	-4.10128000	0.25388600
H	-4.34588200	-4.10445200	0.36813500
H	-3.02452500	-4.51575000	-0.73231400
H	-2.84460100	-4.78619700	1.00350000
C	0.43676700	-3.53979100	2.39480400
H	0.58789100	-3.95538800	1.39416900
H	1.14156400	-4.03348500	3.07401400
H	-0.57566800	-3.80982600	2.71458800
C	0.22305800	-1.47021200	3.78632200
H	-0.84449300	-1.64594900	3.95496100
H	0.76454500	-1.96340800	4.60240100
H	0.40272800	-0.39290200	3.85355400
C	5.41937900	-2.69616400	-1.26879300
H	6.31146300	-2.32618600	-0.75105000
H	5.17460900	-3.67142300	-0.83608200
H	5.69447600	-2.84866500	-2.31759800
C	4.66113800	-0.34915900	-1.72543500
H	5.50845600	0.07596800	-1.17588300
H	4.95609300	-0.44264400	-2.77630000
H	3.83703600	0.36739200	-1.66745500
H	2.53106700	3.37757100	-2.21483400
H	2.83980200	2.34283600	1.93454900
H	-2.49729000	2.67305800	-2.64764900
H	1.36546500	5.27619300	-1.97747200
H	-3.87506200	1.44950100	0.19847200
H	-1.29082300	1.78098300	3.60120100
Al	0.22028900	1.50154700	0.86279900
Al	0.48422700	-0.40463300	-0.93316700
H	0.76074800	1.30533300	2.35673000
H	1.28782500	0.03386200	-2.26437200

phBDIAl

N	1.41864000	0.27297800	0.11151900
C	1.25808700	1.59615200	0.23917400
C	-0.00000100	2.20401000	0.31872000
H	0.00000000	3.27904500	0.46036300
C	-1.25808800	1.59615200	0.23916900
N	-1.41863800	0.27297600	0.11152500
C	2.45644200	2.49961500	0.34309700
H	3.20659400	2.08178700	1.02050000
H	2.16772700	3.48970700	0.69938300
H	2.95316100	2.62351700	-0.62367400
C	-2.45644300	2.49961700	0.34307700
H	-2.95315200	2.62351700	-0.62369900
H	-2.16773000	3.48970800	0.69936400
H	-3.20660200	2.08179100	1.02047300
C	2.73906200	-0.24632500	-0.01836800
C	3.25278000	-1.07776700	0.98168200
C	4.51492500	-1.64802100	0.84128200
H	4.90305700	-2.28912800	1.62771900
C	5.27460400	-1.40208700	-0.29868700
C	4.75995700	-0.58748800	-1.30533300
H	5.33878300	-0.40236400	-2.20609700
C	3.49903100	-0.01723000	-1.17047900
C	-2.73906100	-0.24632700	-0.01836500
C	-3.49902800	-0.01723400	-1.17047700
C	-4.75995500	-0.58748900	-1.30533200
H	-5.33878000	-0.40236400	-2.20609800
C	-5.27460600	-1.40208400	-0.29868500
C	-4.51492700	-1.64801900	0.84128400
H	-4.90306100	-2.28912500	1.62772200
C	-3.25278000	-1.07776900	0.98168500
H	6.25797500	-1.84979200	-0.40768300
H	-6.25797800	-1.84978700	-0.40767900
Al	0.00000000	-1.20786400	0.05281400
H	-2.65472100	-1.26345800	1.87007300
H	-3.08201700	0.59735600	-1.96464100
H	2.65472100	-1.26345500	1.87007100
H	3.08202200	0.59736300	-1.96464100

phBDIAl_d

N	1.44499100	2.20037800	-0.38707600
C	1.67486400	2.85737200	0.75486400
C	0.64991600	3.19040100	1.64973600
H	0.95168700	3.75306600	2.52615900
C	-0.73362900	3.03265200	1.47232200
N	-1.26281800	2.36909200	0.44346600
C	3.05963600	3.32954400	1.10247700
H	3.69070000	2.49681000	1.43166000
H	3.02831100	4.06658000	1.90645500
H	3.55886000	3.77060300	0.23534300
C	-1.62517300	3.69499200	2.48694200
H	-2.45547800	4.21651900	2.00269300
H	-1.06353100	4.40815700	3.09211600
C	2.49852200	1.97175100	-1.31277500
C	3.59731500	1.17125100	-0.99203100

C	4.57524200	0.90673700	-1.94525600
H	5.42093000	0.27745300	-1.67707600
C	4.46697200	1.43083400	-3.23106900
C	3.36347700	2.21483900	-3.56067900
H	3.26341000	2.62156700	-4.56331400
C	2.38077200	2.47941000	-2.61195900
C	-2.67485600	2.24620400	0.34059500
C	-3.34188600	2.84448100	-0.73328600
C	-4.72405400	2.72625300	-0.85248000
H	-5.23430900	3.20927500	-1.68163300
C	-5.44983800	2.00087400	0.08845800
C	-4.78387300	1.37398400	1.13929000
H	-5.34034900	0.78131700	1.86073000
C	-3.40284500	1.48451100	1.26207500
N	-1.32083800	-2.51883700	-0.17072300
C	-0.99756800	-3.78411400	-0.44936000
C	0.26479800	-4.32589300	-0.17659300
H	0.37410000	-5.38631700	-0.37588600
C	1.39744700	-3.68749500	0.34035000
N	1.44046400	-2.37179300	0.60791300
C	-2.01880900	-4.69766400	-1.06489000
H	-2.83532500	-4.91129700	-0.36859700
H	-1.56821600	-5.64349800	-1.36782900
H	-2.48180500	-4.22812700	-1.93789500
C	2.56641500	-4.59368900	0.63209000
H	3.24891400	-4.67510300	-0.21896700
H	2.20362400	-5.60073600	0.84746800
H	3.15605800	-4.24167900	1.48056500
C	-2.67156100	-2.10504100	-0.39977300
C	-3.67607600	-2.45502800	0.50690100
C	-4.99200300	-2.06827600	0.27258700
H	-5.76991100	-2.35078500	0.97726200
C	-5.30893400	-1.31527900	-0.85557100
C	-4.30372800	-0.94068500	-1.74242500
H	-4.54108600	-0.33503500	-2.61288100
C	-2.98611100	-1.33218300	-1.51942600
C	2.66444700	-1.78049900	1.01588900
C	3.85535400	-1.96374500	0.29821800
C	5.04207400	-1.38208300	0.73106100
H	5.95644000	-1.55372900	0.16891800
C	5.05703700	-0.56156700	1.85737900
C	3.86849500	-0.33378700	2.54880200
H	3.86130500	0.30665500	3.42694200
C	2.68450800	-0.94137700	2.13969000
H	-6.52889700	1.91118500	-0.00408300
H	-6.33508800	-1.00509800	-1.03301200
H	5.98258700	-0.09938900	2.18796200
H	5.23125300	1.22231700	-3.97435200
H	-2.87040800	0.97820100	2.06433500
H	-2.76556400	3.42083500	-1.45222900
H	-2.07503400	2.96145000	3.16292100
H	3.66513800	0.74219500	0.00278800
H	1.51519100	3.08810800	-2.86024000
Al	-0.24429700	1.21087200	-0.84804000
Al	-0.15665400	-1.10748300	0.60822200
H	-2.19203100	-1.04410900	-2.20325700
H	-3.41323000	-3.04122200	1.38467500
H	1.76323700	-0.78465200	2.69526000
H	3.84021700	-2.55356500	-0.61334200

phAlHAlHph

N	-1.13024400	-1.99062900	-1.05212600
C	-1.53337700	-3.01818100	-0.30247500
C	-0.71544400	-3.62283200	0.66801100
H	-1.15582100	-4.45702900	1.20291300
C	0.64038500	-3.37237500	0.93427800
N	1.33081300	-2.40188100	0.33618600
C	-2.89733600	-3.61695900	-0.50235700
H	-3.66553900	-3.02409800	0.00645900
H	-2.93550700	-4.63001700	-0.09873400
H	-3.17102300	-3.64331100	-1.56010200
C	1.33808800	-4.27461900	1.91076900
H	2.21509700	-4.73997300	1.44976600
H	0.67387400	-5.06026500	2.27209900
C	-1.99515400	-1.38554700	-2.00381200
C	-3.19606800	-0.78413500	-1.61345700
C	-3.98730000	-0.12679600	-2.54884200
H	-4.91585100	0.33901300	-2.22675500
C	-3.58782600	-0.05371800	-3.88141600
C	-2.38310900	-0.63615800	-4.26970400
H	-2.05912000	-0.57831000	-5.30526500
C	-1.58443400	-1.29269900	-3.33841800
C	2.71321000	-2.22066800	0.62498600
C	3.67622700	-2.68082000	-0.27771400
C	5.02779400	-2.46117200	-0.02962700
H	5.76960200	-2.82928300	-0.73327300
C	5.42949700	-1.76831200	1.11099500
C	4.46983200	-1.28925300	2.00059600
H	4.77335100	-0.73548600	2.88513600
C	3.11590700	-1.51157600	1.76162700
N	1.03842300	2.71710700	0.23827500
C	0.56989600	3.92888800	0.52850600
C	-0.62540100	4.12318500	1.24482200
H	-0.83690900	5.15080800	1.52142200
C	-1.62574200	3.19993700	1.57524100
N	-1.51960300	1.88162200	1.33772900
C	1.32344200	5.16848100	0.13531100
H	2.39984500	4.99263300	0.08354700
H	1.12478300	5.96865100	0.85115400
H	1.00903800	5.53404100	-0.84742600
C	-2.82843300	3.78315700	2.27074800
H	-3.57532900	4.15569300	1.56286500
H	-2.51473300	4.63653600	2.87546400
H	-3.32518000	3.05732500	2.91682200
C	2.18738500	2.51356300	-0.56406000
C	3.16293700	1.62228700	-0.09746500
C	4.25576700	1.29389100	-0.89194300
H	4.99620800	0.59421600	-0.51255000
C	4.39024300	1.85006500	-2.16185100
C	3.42054900	2.73257600	-2.63233700
H	3.50836200	3.15694500	-3.62884900
C	2.32150800	3.05945600	-1.84499000
C	-2.59638900	0.99344800	1.51968100
C	-3.91442200	1.27886700	1.12687200
C	-4.92749600	0.34278800	1.30178200
H	-5.93919000	0.58952000	0.99009000
C	-4.64895600	-0.91289000	1.83990500
C	-3.33674800	-1.22291800	2.19432900
H	-3.09330400	-2.20286300	2.59758000
C	-2.32313300	-0.28423100	2.03811900
H	6.48508200	-1.59594600	1.30179200
H	5.23930100	1.58881300	-2.78655700
H	-5.44133300	-1.64459200	1.96873100
H	-4.20567800	0.46193900	-4.61107500
H	2.35772000	-1.11905300	2.43640900
H	3.34758600	-3.20510000	-1.17090700
H	1.71207800	-3.71003300	2.77054100
H	-3.48974800	-0.82391200	-0.56923400
H	-0.63667400	-1.73772000	-3.62812200
Al	0.51324000	-0.94901400	-0.71688900

Al	0.25009500	1.05261300	0.94969800
H	1.31321700	-0.83475200	-2.09399500
H	0.90295000	0.82261600	2.41131200
H	1.54225300	3.70979200	-2.23231800
H	3.05966000	1.19684200	0.89999300
H	-1.30820000	-0.52979800	2.34884700
H	-4.13536600	2.22745000	0.64860400
